# CAM evolution is associated with gene family expansion in an explosive bromeliad radiation

**DOI:** 10.1093/plcell/koae130

**Published:** 2024-04-30

**Authors:** Clara Groot Crego, Jaqueline Hess, Gil Yardeni, Marylaure de La Harpe, Clara Priemer, Francesca Beclin, Sarah Saadain, Luiz A Cauz-Santos, Eva M Temsch, Hanna Weiss-Schneeweiss, Michael H J Barfuss, Walter Till, Wolfram Weckwerth, Karolina Heyduk, Christian Lexer, Ovidiu Paun, Thibault Leroy

**Affiliations:** Department of Botany and Biodiversity Research, University of Vienna, 1030 Vienna, Austria; Vienna Graduate School of Population Genetics, Vienna, Austria; Department of Botany and Biodiversity Research, University of Vienna, 1030 Vienna, Austria; Cambrium GmbH, Max-Urich-Str. 3, 13055 Berlin, Germany; Department of Botany and Biodiversity Research, University of Vienna, 1030 Vienna, Austria; Department of Biotechnology, Institute of Computational Biology, University of Life Sciences and Natural Resources (BOKU), Muthgasse 18, 1190 Vienna, Austria; Department of Botany and Biodiversity Research, University of Vienna, 1030 Vienna, Austria; Office for Nature and Environment, Department of Education, Culture and Environmental protection, Canton of Grisons, 7001 Chur, Switzerland; Department of Functional and Evolutionary Ecology, Molecular Systems Biology (MOSYS), University of Vienna, 1030 Vienna, Austria; Department of Botany and Biodiversity Research, University of Vienna, 1030 Vienna, Austria; Vienna Graduate School of Population Genetics, Vienna, Austria; Gregor Mendel Institute, Austrian Academy of Sciences, Vienna BioCenter, 1030 Vienna, Austria; Department of Botany and Biodiversity Research, University of Vienna, 1030 Vienna, Austria; Vienna Graduate School of Population Genetics, Vienna, Austria; Department of Botany and Biodiversity Research, University of Vienna, 1030 Vienna, Austria; Department of Botany and Biodiversity Research, University of Vienna, 1030 Vienna, Austria; Department of Botany and Biodiversity Research, University of Vienna, 1030 Vienna, Austria; Department of Botany and Biodiversity Research, University of Vienna, 1030 Vienna, Austria; Department of Botany and Biodiversity Research, University of Vienna, 1030 Vienna, Austria; Department of Functional and Evolutionary Ecology, Molecular Systems Biology (MOSYS), University of Vienna, 1030 Vienna, Austria; Vienna Metabolomics Center (VIME), University of Vienna, 1030 Vienna, Austria; Department of Ecology and Evolutionary Biology, University of Connecticut, Storrs, CT 06269, USA; Department of Botany and Biodiversity Research, University of Vienna, 1030 Vienna, Austria; Department of Botany and Biodiversity Research, University of Vienna, 1030 Vienna, Austria; Department of Botany and Biodiversity Research, University of Vienna, 1030 Vienna, Austria; GenPhySE, Université de Toulouse, INRAE, ENVT, 31326 Castanet Tolosan, France

## Abstract

The subgenus *Tillandsia* (Bromeliaceae) belongs to one of the fastest radiating clades in the plant kingdom and is characterized by the repeated evolution of Crassulacean acid metabolism (CAM). Despite its complex genetic basis, this water-conserving trait has evolved independently across many plant families and is regarded as a key innovation trait and driver of ecological diversification in Bromeliaceae. By producing high-quality genome assemblies of a *Tillandsia* species pair displaying divergent photosynthetic phenotypes, and combining genome-wide investigations of synteny, transposable element (TE) dynamics, sequence evolution, gene family evolution, and temporal differential expression, we were able to pinpoint the genomic drivers of CAM evolution in *Tillandsia*. Several large-scale rearrangements associated with karyotype changes between the 2 genomes and a highly dynamic TE landscape shaped the genomes of *Tillandsia*. However, our analyses show that rewiring of photosynthetic metabolism is mainly obtained through regulatory evolution rather than coding sequence evolution, as CAM-related genes are differentially expressed across a 24-h cycle between the 2 species but are not candidates of positive selection. Gene orthology analyses reveal that CAM-related gene families manifesting differential expression underwent accelerated gene family expansion in the constitutive CAM species, further supporting the view of gene family evolution as a driver of CAM evolution.

## Teaser

The genomes of 2 species belonging to the *Tillandsia* radiation reveal gene family expansion as a potential driver of CAM evolution.

## Introduction

Crassulacean acid metabolism (CAM) is a photosynthetic phenotype playing a major role in plant adaptation to arid environments and the epiphytic lifeform (Cushman 2001; Silvera et al. 2010; Winter and Smith 2012), and has been described as a key innovation trait driving plant diversification and speciation in several plant lineages (Ogburn and Edwards 2009; Silvera et al. 2009; Quezada and Gianoli 2011). CAM functions as a carbon concentrating mechanism by assimilating CO_2_ overnight and storing it as malate in the vacuole, which greatly enhances the efficiency of Rubisco, the first enzyme of the Calvin cycle (Osmond 1978). This also has the secondary effect of improving the plant's overall water use efficiency by reducing evapotranspiration, as stomata can remain closed during the day (Borland et al. 2014). Though often presented as a discrete trait, CAM actually encompasses a large spectrum of photosynthetic phenotypes including intermediate and facultative forms (Edwards 2023). Phenotypes from this CAM continuum have evolved repeatedly in at least 37 plant families (Winter et al. 2021), yet the underlying evolutionary mechanisms allowing this complex and diverse trait to emerge multiple times throughout plant history are not fully understood.

Due to the sparse availability of CAM plant genomes, most studies on CAM evolution have focused on transcription levels and sequence evolution to understand its underlying genetic drivers. However, novel variation can be generated by other mechanisms which have not been investigated thoroughly in the context of CAM evolution. For example, several studies have suggested a potential importance of gene family expansion as a driver of CAM evolution (Silvera et al. 2014; Cai et al. 2015). In C4 plants, duplicated gene copies tend to be more often retained compared to closely related C3 lineages (Hoang et al. 2023). Gene duplication occurs at higher rates than point mutation in many lineages (Katju and Bergthorsson 2013) and can lead to novel functional variation through dosage effects, neofunctionalization, or subfunctionalization (Ohno 1970), as observed in teleost fish (Arnegard et al. 2010; Moriyama et al. 2016) and orchids (Mondragón-Palomino and Theissen 2009). Another form of structural variation that can contribute to the evolution of complex traits is transposable element (TE) insertion in and around genes, which has been shown to play a role in local adaptation, for example in *Arabidopsis thaliana* (Baduel et al. 2019). Finally, large-scale rearrangements such as chromosomal fusions, inversions, or translocations can increase linkage between co-adapted alleles and generate reproductive barriers (Lowry and Willis 2010; Luo et al. 2018).

The adaptive radiation of *Tillandsia* subgenus *Tillandsia* (Bromeliaceae) is part of one of the fastest diversifying clades known in the plant kingdom (Tillandsioideae) (Givnish et al. 2014) and is characterized by a number of key innovation traits such as the epiphytic lifestyle, absorptive trichomes, water-impounding tanks, and photosynthetic metabolism driving extraordinary diversity both on the taxonomic and ecological level (Barfuss et al. 2016). The group displays a broad range of phenotypes of the CAM continuum, resulting from repeated evolution of constitutive CAM (Crayn et al. 2015; De La Harpe et al. 2020). CAM evolution has been described as an ecological driver of diversification in the subgenus *Tillandsia* (Crayn et al. 2004; Barfuss et al. 2016), and across Bromeliaceae in general (Benzing and Bennett 2000; Crayn et al. 2004; Givnish et al. 2014). This renders the radiation a fascinating system both for studies on speciation and rapid adaptation generally, and for studies on CAM evolution specifically, as comparative investigations between recently diverged species with contrasting phenotypes prevent the overestimation of evolutionary changes needed to evolve adaptations such as CAM (Heyduk et al. 2019a).

While Bromeliaceae is generally regarded as a homoploid radiation with conserved chromosome counts and little genome size variation (Gitaí et al. 2014), more recent work has pointed at a high “genomic potential” of the subgenus *Tillandsia*, notably from elevated gene loss and duplication rates (De La Harpe et al. 2020), providing an exemplary system to study the role of genome evolution and structural variation in CAM evolution. Not only are adaptive radiations like *Tillandsia* characterized by repeated evolution of key innovation traits, the short timescales at which novel variation arises in these systems challenge classical views of adaptive evolution, stimulating a range of studies pointing at the potential importance of genome evolution as a genomic driver of diversification (Brawand et al. 2015; McGee et al. 2020; Cicconardi et al. 2021).

**Figure 1.**
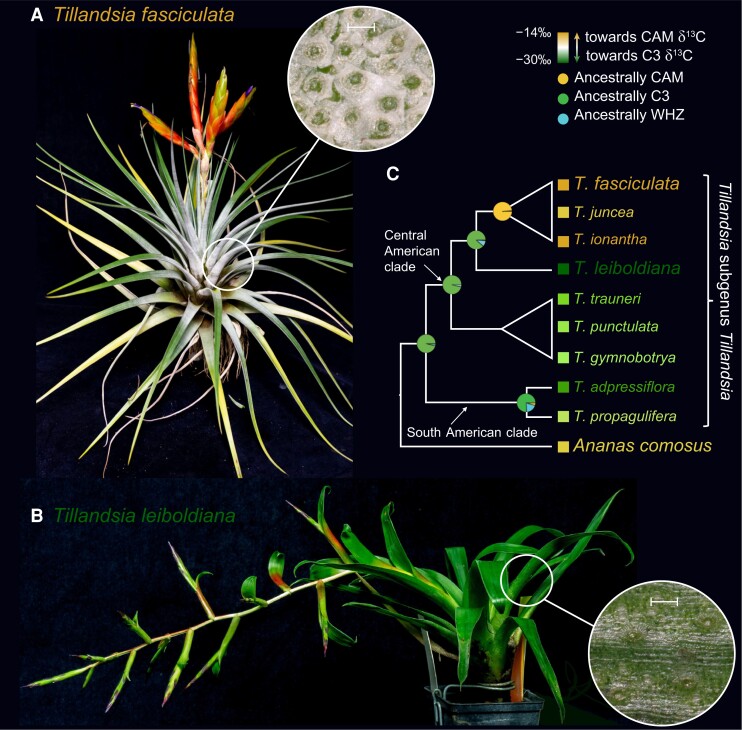
Presentation of the 2 species investigated in this study and overview of known CAM phenotypes and evolution in the subgenus *Tillandsia*. **A)***Tillandsia fasciculata*, a “gray” or “atmospheric” *Tillandsia* with a dense layer of umbrella-shaped trichomes (inset), carbon isotope values within the CAM range, a lack of water-impounding tank, and roots adapted to the epiphytic lifestyle. The leaf close-up is at a 100 *μ*m scale (also in **B**). Photograph by Clara Groot Crego. **B)***Tillandsia leiboldiana*, a green *Tillandsia* with C3-like leaf morphology and carbon isotope values, an impounding tank, and a sparse trichome layer (inset). Photograph by Clara Groot Crego **C)** Schematic representation of the evolutionary relationship between the 2 investigated species of *Tillandsia* within the subgenus (modified from De La Harpe et al. 2020). Colors indicate reported carbon isotope values (Crayn et al. 2015; De La Harpe et al. 2020). The average was taken when multiple values have been reported for the same species. Pie charts at internal nodes show the ancestral state of photosynthetic metabolism as reported in De La Harpe et al. 2020. WHZ stands for Winter-Holtum Zone (Males 2018) and represents intermediate forms of the CAM continuum.

In this study, we comparatively investigated *de novo* assembled genomes of 2 ecologically divergent members of the subgenus *Tillandsia* to further our understanding of genome evolution in this recent radiation and its link to CAM evolution as a key innovation trait. The giant airplant (*Tillandsia fasciculata*) (Fig. 1A) displays a set of phenotypes typically described as “gray” or “atmospheric” *Tillandsia* (Benzing and Bennett 2000): a dense layer of absorptive, umbrella-shaped trichomes, CAM photosynthesis, and occurrence in arid places with high solar incidence and low rainfall. On the other hand, *Tillandsia leiboldiana* (Fig. 1B) represents a typical “green” *Tillandsia* displaying tank formation, C3-like leaf morphology, a sparse layer of absorptive trichomes, and occurrences in cooler, wetter regions. While not sister species, *T. fasciculata* and *T. leiboldiana* belong to sister clades displaying a shift in photosynthetic metabolism (Fig. 1C), and represent phenotypic extremes within subgenus *Tillandsia*. Their photosynthetic metabolisms have been described as strong CAM for *T. fasciculata*, and C3 for *T. leiboldiana* based on carbon isotope ratios (δ^13^C*_T. fasciculata_* = −11.9/−16.1; δ^13^C*_T. leiboldiana_* = −28.0/−31.3) reported by Crayn et al. (2015) and De La Harpe et al. (2020), respectively. These are some of the most distinct values reported for the subgenus.

However, due to the limited ability of carbon isotope measurements in capturing intermediate CAM phenotypes and therefore representing the full CAM continuum (Pierce et al. 2002; Messerschmid et al. 2021), the exact photosynthetic phenotypes of *T. leiboldiana* and *T. fasciculata* need to be corroborated to fully understand what range of the CAM continuum is truly encompassed in the subgenus. By characterizing the photosynthetic metabolisms of *T. leiboldiana* and *T. fasciculata* and investigating genomic variation between these 2 species on multiple levels, from karyotype, chromosomal rearrangements, to molecular evolution, gene family evolution and temporal differential gene expression, we thoroughly explored the degree of genomic divergence found within this radiation and the link between this variation and the evolution of a key innovation trait. We ascertained that the photosynthetic metabolisms of these species are clearly distinct, with *T. fasciculata* at the late stages of CAM evolution (i.e. constitutive, strong CAM), and *T. leiboldiana* likely at the very early stages (i.e. no night-time malate accumulation, but CAM-like expression profiles of certain enzymes). We further documented karyotype differences, multiple chromosomal rearrangements, distinct TE landscapes and gene family evolution rates between the 2 species. Molecular variation underlying the difference in phenotype was largely found at the transcriptomic level, yet we also observed a clear association between CAM-related temporal gene expression differences and both gene family expansion in the constitutive CAM plant and pre-existing gene duplications shared between both species.

## Results

### Photosynthetic phenotypes of *T. fasciculata* and *T. leiboldiana*

**Figure 2.**
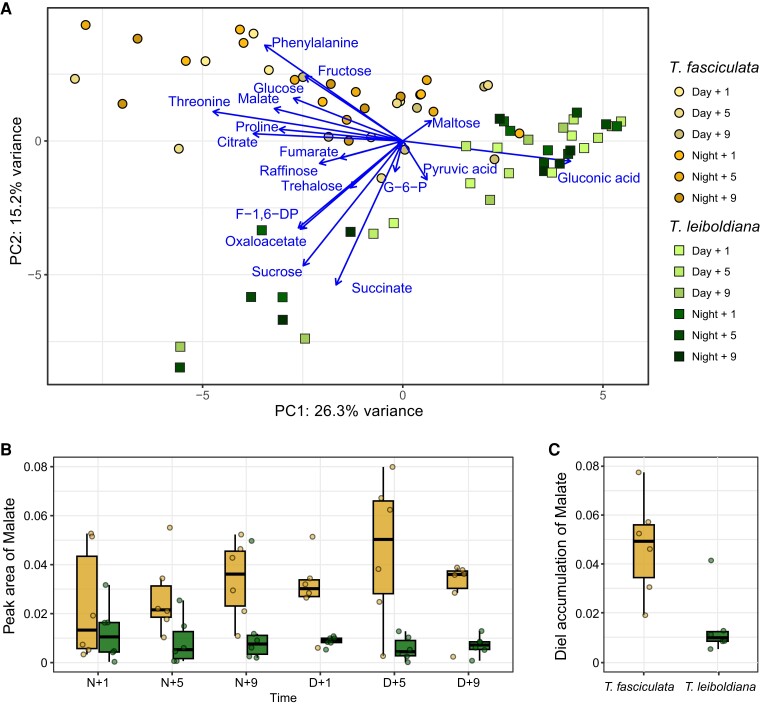
Metabolomic analyses of *T. fasciculata* and *T. leiboldiana* leaf material throughout a 24-h cycle. Six accessions of distinct genotype per species were sampled across 6 time points. Abundances of individual metabolites were measured with GC-MS and normalized against the Main Total Ion Count (MTIC). **A)** Principal component analysis of metabolic composition of 72 leaf samples based on 77 metabolic compounds including soluble sugars, amino acids, and organic acids. The first and second principal components are displayed. Arrows show the loadings of a subset of metabolites relevant for photosynthetic metabolism. **B)** Malate abundance in leaf material of *T. fasciculata* and *T. leiboldiana* at 6 time points across a 24-h cycle. Dots represent individual observations across time points. Time points are noted as hours into the day (D) or into the night (N). Whiskers reach to the minimum and maximum value; lower box border shows the 25th percentile, upper box border the 75th percentile. The thick horizontal line inside the box represents the median. **C)** Distribution of per-accession accumulation of malate per species over a 24-h cycle. Accumulations were obtained by taking the difference in malate abundance between the highest and lowest reported abundances across time for each accession. Description of boxplots is identical to **B)**.

To better understand the difference in photosynthetic metabolism between *T. fasciculata* and *T. leiboldiana*, we measured metabolite abundances with GC-MS for 6 samples per species at 6 time points across a 24-h cycle (Supplementary Data Set 1). The overall composition of 49 metabolite abundances separate samples of both species within the first 2 principal components (Fig. 2A), which combined explain 41.5% of variance. This suggests a pronounced general metabolic differentiation between *T. fasciculata* and *T. leiboldiana*. Besides amino acids, the organic acids malate, citrate, and gluconic acid contribute most to this differentiation along PC1 (Supplementary Fig. S1), which is a common pattern for species with diverging photosynthetic metabolism (Benzing and Bennett 2000; Popp et al. 2003; De La Harpe et al. 2020). Sugars appear large contributors to differentiation along PC2 (Supplementary Fig. S1).

Overnight malate accumulation is a core feature of CAM photosynthesis and therefore an indicator of the respective photosynthetic phenotypes of *T. fasciculata* and *T. leiboldiana*. Malate abundances in the leaf fluctuated strongly in *T. fasciculata* over 24 h, with highest median abundances around midday (D + 5) and lowest abundances in the early night (N + 1), representing a 3.8-fold difference (Fig. 2B). In comparison, malate abundances were overall lower for *T. leiboldiana* and fluctuated less. The highest median abundance in the latter species was found at N + 1, while the lowest was found at D + 5, representing a 2.2-fold difference. Interestingly, the accumulation times of malate seem reversed in the 2 species, with the highest abundances in *T. fasciculata* found at the time of lowest abundances in *T. leiboldiana* and vice versa. The reversed timing of malate accumulation has been described as a key difference between C3 and CAM metabolisms (Winter and Smith 2022, but see also Bräutigam et al. 2017). The median accumulation in malate within 24 h differs significantly between the species, with a 3.2-fold higher value in *T. fasciculata* than in *T. leiboldiana* (Mann–Whitney *U*, *P*-value = 8.6E^−03^, Fig. 2C, Supplementary Data Set 2).

Overall, the malate accumulation curves suggest distinct photosynthetic phenotypes for *T. fasciculata* and *T. leiboldiana*. *T. fasciculata* appears to behave as a constitutive CAM plant in standard conditions, accumulating malate from the early night until the early day, while *T. leiboldiana*'s flux is more C3-like, without a clear accumulation overnight.

### Genome assembly and annotation

We constructed *de novo* haploid genome assemblies for both species (Supplementary Table S1) using a combination of long-read (PacBio), short-read (Illumina), and chromosome conformation capture (Hi-C) data. This resulted in assemblies of 838 Mb and 1,198 Mb with an N50 of 23.6 and 43.3 Mb in *T. fasciculata* and *T. leiboldiana*, respectively. The assembly sizes closely match the estimated genome size of each species based on flow cytometry and k-mer analysis (Supplementary Table S2, Supplementary SI Notes S1 and S2, Supplementary Figs. S2 and S3). The 25 and respectively 26 longest scaffolds (hereafter referred to as “main scaffolds”) contain 72% and 75.5% of the full assembly, after which scaffold sizes steeply decline (Supplementary SI Note S3, Supplementary Fig. S4). The number of main scaffolds corresponds with the species karyotype in *T. fasciculata*, but deviates from the *T. leiboldiana* karyotype (Supplementary Fig. S5, Supplementary SI Note S1), suggesting that a few fragmented chromosome sequences remain in the latter assembly.

Structural gene annotation resulted in a total of 34,886 and 38,180 gene models in *T. fasciculata* and *T. leiboldiana*, respectively, of which 92.6% and 71.9% are considered robust based on additional curation (Materials and methods, Gene model assessment and curation). Annotation completeness was evaluated with BUSCO using the Liliopsida data set resulting in a score of 89.7% complete genes in *T. fasciculata* and 85.3% in *T. leiboldiana* (Supplementary Table S2).

### Genic, repetitive, and GC content

TE annotation performed with EDTA (Ou et al. 2019) revealed a total repetitive content of 65.5% and 77.1% in *T. fasciculata* and *T. leiboldiana*, respectively. This closely matches estimates derived from k-mer analyses (66% and 75%, Supplementary SI Note S2). Compared to *T. fasciculata*, the repetitive content in *T. leiboldiana* is enriched for Gypsy LTR retrotransposon and *Mutator* DNA transposon content, with a 1.7-fold and 4.2-fold increase in total covered genomic length, respectively (Supplementary Table S3).

**Figure 3.**
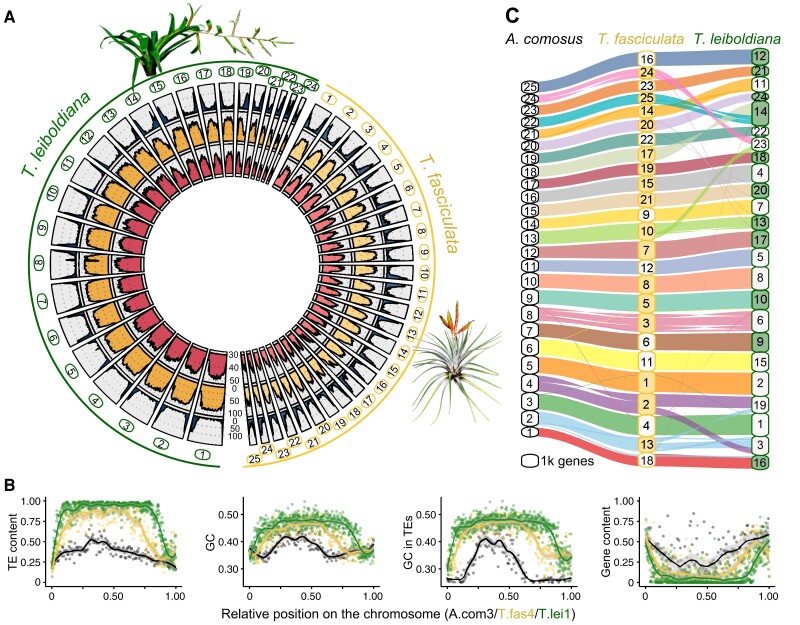
Spatial composition and synteny of the *T. fasciculata* and *T. leiboldiana* genomes. **A)** Circular overview of the main scaffolds of the *T. fasciculata* (right) and *T. leiboldiana* (left) genome assemblies. Scaffolds 25 and 26 of *T. leiboldiana* are not shown due to their reduced size. Going inwards, the tracks show: (1, blue) gene count; (2, yellow) proportion of transposable element (TE) content; (3, red), and GC content per 1-Mb windows. **B)** TE and GC contents, GC content exclusively in TEs, and genic content in a triplet of syntenic scaffolds between *Ananas comosus* (LG3, black), *T. fasciculata* (scaffold 4, gray), and *T. leiboldiana* (scaffold 1, green; see Supplementary Fig. S6 for other syntenic chromosomes). Species names are abbreviated as A.com, T.fas, and T.lei, respectively. Note that the TE content was directly estimated on the soft-masked positions of the 3 reference genomes. Given that TE annotation approach used on the *Tillandsia* genomes differs from that on the *A. comosus* (F153) genome, the observed among-species difference in TE content should not be interpreted too strictly. **C)** Syntenic plot linking blocks of orthologous genes between *A. comosus*, *T. fasciculata*, and *T. leiboldiana*. The size of each scaffold on the *y* axis is proportional to genic content and therefore does not represent the true scaffold size. Color-filled boxes indicate scaffolds with reversed coordinates as compared to the sequences in *A. comosus*.

Repetitive content per scaffold is negatively correlated with gene count in both assemblies (Kendall's correlation coefficient: −0.79 in *T. fasciculata*, −0.82 in *T. leiboldiana*, *P*-values < 2.2E^−16^, Supplementary Data Set 2), with gene-rich regions in distal positions (Fig. 3A, green track) and repetitive regions primarily in median positions (Fig. 3A, yellow track). This pattern is accentuated in *T. leiboldiana*: on average, the repetitive-to-exonic content per scaffold is 1.6 times larger compared to that in *T. fasciculata* (Welch's T, *P*-value = 4.3E^−04^, Supplementary Data Set 2). The genome size difference between the 2 assemblies is therefore mostly explained by differential accumulation of TE content, mostly in heterochromatic regions.

GC content was negatively correlated with gene content in both species (Kendall's correlation coefficient: −0.68 in *T. fasciculata*, −0.71 in *T. leiboldiana*, *P*-values < 2.2E^−16^, red track in Fig. 3A, detailed in Fig. 3B, Supplementary Data Set 2). By visualizing GC and TE contents across a syntenic chromosome triplet of pineapple (*Ananas comosus*), *T. fasciculata*, and *T. leiboldiana*, we show that this relationship can be mostly explained by elevated GC content in repetitive regions (Fig. 3B). TE-rich regions indeed exhibit a much higher GC content than TE-poor regions, a pattern which is exacerbated as the overall TE content per species increases (Fig. 3B, Supplementary Fig. S6, Supplementary SI Note S4).

### Synteny and chromosomal evolution

Cytogenetic karyotyping (Supplementary SI Note S1, Supplementary Fig. S5) revealed a difference of 6 chromosome pairs between *T. fasciculata* (2*n* = 50) and *T. leiboldiana* (2*n* = 38), which is atypical in this largely homoploid clade with generally constant karyotype (Brown and Gilmartin 1989; Gitaí et al. 2014). To investigate orthology and synteny, we inferred orthogroups between protein sequences of *A. comosus* (Ming et al. 2015) (pineapple), *T. fasciculata*, and *T. leiboldiana* using Orthofinder (Emms and Kelly 2019). This resulted in 21,045 (78%), 26,325 (87.5%), and 23,584 (75%) gene models assigned to orthogroups, respectively, of which 10,021 were single-copy orthologs between all 3 species (Supplementary Table S4).

Syntenic blocks were then defined across all 3 assemblies using GENESPACE (Lovell et al. 2022) (Fig. 3C). Remarkably, the 3-way synteny analysis between *A. comosus*, *T. fasciculata*, and *T. leiboldiana* showed higher synteny between *T. fasciculata* and *A. comosus* than between the 2 *Tillandsia* genomes, which could be explained by *T. leiboldiana*'s diverged karyotype. While the difference in karyotype could have arisen from chromosomal loss in *T. leiboldiana*, our GENESPACE analysis revealed conserved synteny between the 2 *Tillandsia* assemblies without major orphan regions in *T. leiboldiana*. This is consistent with a scenario of chromosomal fusion, rather than loss. We found clear evidence of such a fusion on scaffold 14 in *T. leiboldiana* (Fig. 3C, Supplementary Fig. S7A), which was confirmed with in-depth analyses of potential breakpoints (Supplementary SI Note S5). However, chromosomal rearrangements are not limited to fusions, since we also detected 2 major reciprocal translocations (Fig. 3C, hereafter referred to as Translocations 1 and 2, Supplementary Fig. S7B and C).

### Gene family evolution

**Figure 4.**
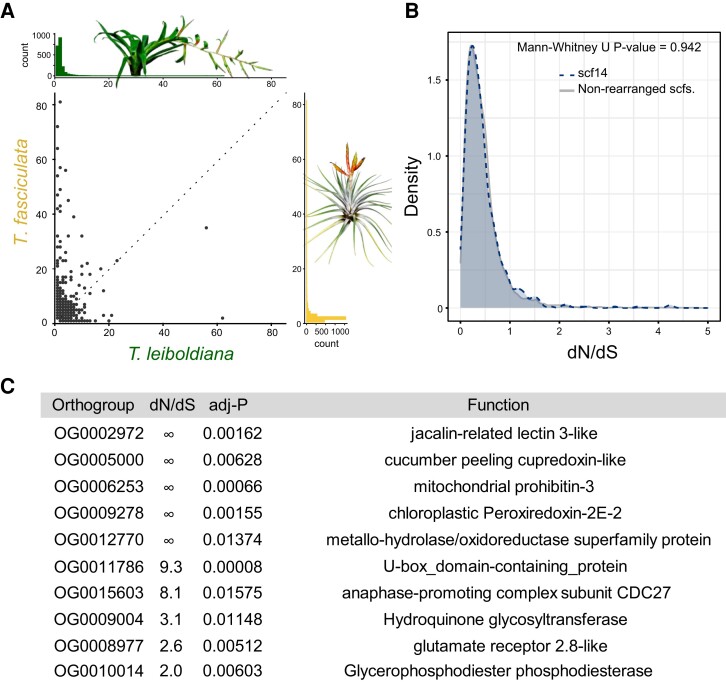
Analyses of gene family evolution and adaptive sequence evolution linked to large-scale rearrangements between *T. fasciculata* and *T. leiboldiana.***A)** Scatterplot: composition of per-species gene counts among orthogroups. Upper histogram: distribution of per-orthogroup gene count in *T. leiboldiana*. Lower histogram: distribution of per-orthogroup gene count in *T. fasciculata*. **B)** Density plot showing the distribution of *d*_N_/*d*_S_ values of one-to-one orthologs across non-rearranged scaffolds (gray profile) and scaffold 14 in *T. leiboldiana* (blue profile), which is the result of a fusion. **C)** Single-copy orthogroups with significant *d*_N_/*d*_S_ values and their functions. Three uncharacterized genes that are excluded here are detailed in Supplementary Table S6. Infinite *d*_N_/*d*_S_ values correspond to genes with *d*_S_ = 0 (no synonymous substitutions), an expected situation considering the low divergence of the 2 species. Further explanation about the biological significance of these functions can be found in Supplementary SI Note S7.

A total of 6,261 genes in *T. fasciculata* and 4,693 genes in *T. leiboldiana* were assigned to nonunique gene families with multiple gene copies in at least 1 species, after correcting gene family sizes (Supplementary Table S4). On average, the multicopy gene family size is 1.3× larger in *T. fasciculata* than in *T. leiboldiana* (Mann–Whitney *U*, *P*-value: 8.8E^−16^, Fig. 4A, Supplementary Data Set 2).

To investigate the role of expanded gene families in CAM evolution, we combined gene ontology (GO) enrichment tests on multicopy orthogroups (Supplementary SI Note S6) with a targeted search of known genes involved in the CAM pathway. This highlighted 25 multicopy gene families encoding proteins with functions putatively related to CAM (Supplementary Table S5), of which 17 have expanded in *T. fasciculata* and 8 in *T. leiboldiana*. The gene families expanded in *T. fasciculata* included 1 encoding a malate dehydrogenase (MDH) and another encoding β-carbonic anhydrase (CA), which are putatively involved in the carbon fixation module of CAM photosynthesis, and subunits of the 2 vacuolar pumps (V-ATPase and V-PPiase) known to energize the night-time transport of malate in pineapple (McRae et al. 2002) (Supplementary Table S5). Additionally, 2 families encoding enolases (members of the glycolysis pathway), a family encoding a vacuolar acid invertase putatively involved in day-time soluble sugar accumulation in the vacuole (McRae et al. 2002; Holtum et al. 2005), and a family encoding a pyrophosphate-dependent phosphofructokinase associated with night-time conversion of soluble sugars through glycolysis to PEP (Carnal and Black 1989) were expanded in *T. fasciculata*. Two families encoding subunits of succinate dehydrogenase, a member of the tricarboxylic acid cycle and the electron transport chain which also plays a role in stomatal opening regulation, a relevant aspect of CAM photosynthesis (Araújo et al. 2011), were also expanded in *T. fasciculata*. The gene family encoding XAP5 CIRCADIAN TIMEKEEPER (XCT), a regulator of circadian rhythm and disease resistance (Liu et al. 2022) which was previously identified as undergoing rapid gene family evolution in *Tillandsia* (De La Harpe et al. 2020) is also expanded in *T. fasciculata*.

Gene families expanded in *T. leiboldiana* contained 3 families encoding glycolysis enzymes, 2 encoding aquaporins, 1 encoding an enzyme of the tricarboxylic acid cycle, and lastly, 1 family encoding a regulator of stomatal opening (Supplementary Table S5).

### Adaptive sequence evolution

Adaptive sequence evolution was evaluated in 9,077 one-to-one orthologous gene pairs using the non-synonymous to synonymous substitution ratio (ω = *d*_N_/*d*_S_). Little among-scaffold variation in *d*_N_/*d*_S_ was observed, with per-scaffold median *d*_N_/*d*_S_ values ranging from 0.32 to 0.39 in *T. fasciculata* and 0.31 to 0.4 in *T. leiboldiana* (Supplementary Fig. S8A). Regions of large chromosomal rearrangement such as the fused scaffold 14 in *T. leiboldiana* do not exhibit strong signatures of fast coding sequence evolution (Fig. 4B), though for Translocation 1, *d*_N_/*d*_S_ values are slightly, yet significantly, lower for scaffold 13 in *T. fasciculata* and scaffold 19 in *T. leiboldiana* (Supplementary Fig. S8B, Supplementary SI Note S5).

Among the 9,077 orthologous gene pairs, 13 candidates (0.21%) exhibit a significant *d*_N_/*d*_S_ > 1 (adjusted *P*-value < 0.05, Fig. 4C, Supplementary Table S6, Supplementary SI Note S7). Notably, we recover a significant signal in a type B glycerophosphodiester phosphodiesterase (GDPDL-7). GDPDL's are involved in cell wall cellulose accumulation and pectin linking, and play a role in trichome development (Hayashi et al. 2008), a main trait differentiating the 2 species and more broadly, green and gray *Tillandsia*. Additionally, GDPDL-7 may be involved in response to drought and salt stress (Cheng et al. 2011).

A glutamate receptor (GLR) 2.8-like also exhibits a significant *d*_N_/*d*_S_ > 1. By mediating Ca^2+^ fluxes, GLRs act as signaling proteins and mediate a number of physiological and developmental processes in plants (Weiland et al. 2015), including stomatal movement (Kong et al. 2016). Although it is associated with drought-stress response in *Medicago truncatula* (Philippe et al. 2019), the specific function of GLR2.8 still remains unclear.

### Gene expression analyses

**Figure 5.**
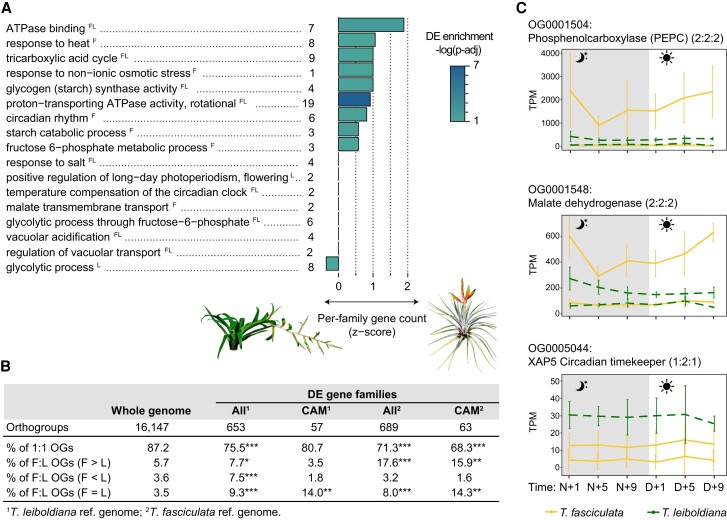
Enrichment and expression curves of multicopy orthogroups among orthogroups with timewise differentially expressed genes between *T. fasciculata* and *T. leiboldiana*. Timewise differentially expressed genes between species were obtained by sampling 6 genotypes per species across 6 time points over a 24-h cycle. RNA-seq reads were mapped to the genomes of both species and 2 separate analyses of differential gene expression were performed in *maSigPro*. For more information, see Materials and methods, Differential gene expression analysis, and Supplementary SI Note S9. **A)** CAM-related enriched GO terms among differentially expressed (DE) genes between *T. fasciculata* and *T. leiboldiana*. The letters F and L indicate whether a GO term was found enriched in the DE analysis using the *T. fasciculata* or *T. leiboldiana* genome assembly as reference genome, respectively. The family size difference for the underlying orthogroups is represented as a *z*-score: a negative score indicates a tendency toward gene families with larger size in *T. leiboldiana* than in *T. fasciculata*, and vice versa. The *P*-value displayed represents the significance of the GO-term enrichment among DE genes in the analysis using the *T. fasciculata* assembly as reference unless the term was only enriched in the analysis with the *T. leiboldiana* assembly as reference. The number of DE genes underlying each function is shown next to the GO-term name. The color gradient of the bars represents the adjusted *P*-value of the enrichment test on a logarithmic scale. **B)** Composition of orthogroups by relative size between *T. fasciculata* and *T. leiboldiana* for 3 orthogroup subsets (whole genome (i.e. all orthogroups), DE orthogroups, and CAM-related DE orthogroups). Species-specific orthogroups are not included in this analysis. F and L stand for the number of genes assigned to a specific orthogroup in *T. fasciculata* and *T. leiboldiana*, respectively, i.e. F > L indicates orthogroups with a higher gene count in *T. fasciculata* than in *T. leiboldiana*. A chi-square test of independence was applied to test the significance of composition changes in 2 × 2 contingency tables for each category when testing the entire DE orthogroup subset against non-DE orthogroups. For CAM-DE orthogroups, the Fisher's exact test was applied. Significant *P*-values of both tests are reported as: *0.05–0.01, **0.01–0.0001, ***0.0001–0. Exact *P*-values and other details on the statistical testing can be found in Supplementary Data Set 2. **C)** Expression profiles in a 24-h period of exemplary CAM-related gene families (phosphoenolpyruvate carboxylase [PEPC], malate dehydrogenase [MDH], and XAP5 CIRCADIAN TIMEKEEPER [XCT]) displayed at the orthogroup level. The number of genes assigned to each orthogroup is displayed in brackets next to the orthogroup name for (*A. comosus*: *T. fasciculata*: *T. leiboldiana*), respectively. For each gene copy and time point, the average read count (in transcripts per million, TPM), and the standard deviation across accessions are displayed. Read counts of each ortholog are obtained by mapping conspecific accessions to their conspecific reference genome. We show 2 families with older duplications preceding the split of *T. fasciculata* and *T. leiboldiana* (PEPC and MDH) and 1 gene family with a recent duplication in *T. fasciculata* (XCT).

To study gene expression differences linked to distinct photosynthetic phenotypes, we performed a time-series RNA-seq experiment using 6 plants of each species (Supplementary Table S1, Supplementary SI Note S8), sampled every 4 h in a 24-h period. We recovered 907 genes with a differential temporal expression (DE) profile between *T. fasciculata* and *T. leiboldiana*. Among them are 46 known CAM-related genes and 22 genes associated with starch metabolism and glycolysis/gluconeogenesis (Supplementary Fig. S9). GO-term enrichment of the 907 DE genes revealed many CAM-related functions such as malate and oxaloacetate transport, circadian rhythm, light response, water and proton pumps, sucrose and maltose transport, and starch metabolism (Supplementary Table S7; Fig. 5A). While none of the candidate genes for adaptive sequence evolution recovered in this study were differentially expressed, 9 of 22 genes reported by De La Harpe et al. 2020 as candidates for adaptive sequence evolution during transitions to constitutive CAM in the wider context of the genus *Tillandsia* were also differentially expressed in this study (Supplementary Table S7).

**Figure 6.**
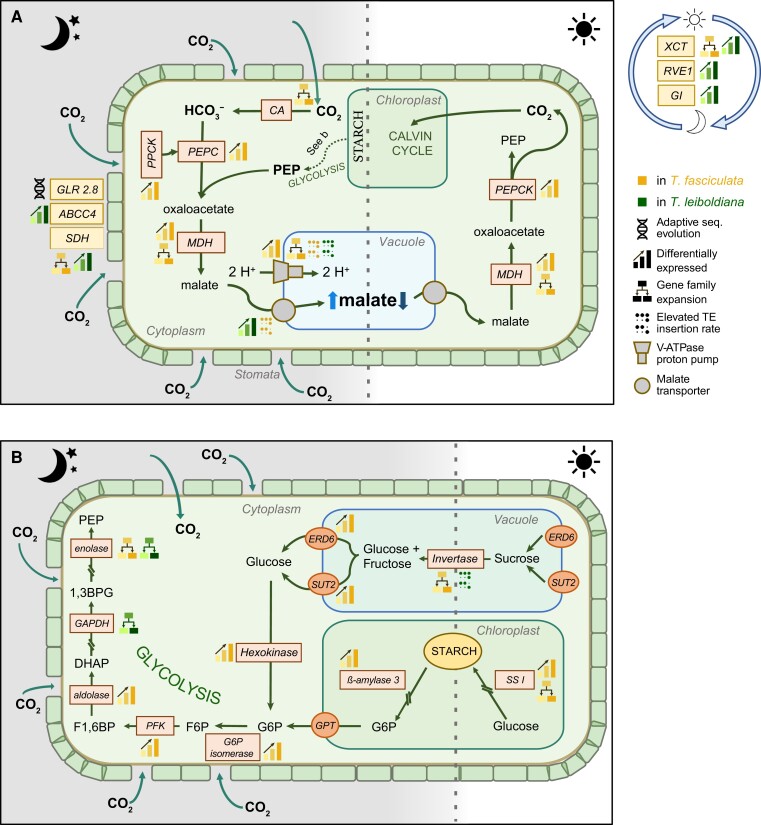
Pathway of Crassulacean acid metabolism (CAM), highlighting underlying genes detected in this study as differentially expressed, with gene family expansion, with signature of adaptive sequence evolution or elevated TE insertion counts. The color of the process symbols indicates the species showing diel or elevated expression, increased gene family size, or increased TE insertion rate. Enzymes are shown in boxes, while pathway products are shown in bold, outside boxes. Enzymatic members of CAM metabolism pathways are shown in orange boxes, while stomatal and circadian regulators are highlighted in yellow. Stomatal regulators are shown on the left outside the cell and circadian regulators on the right. **A)** CO_2_ is absorbed at night and first converted to HCO_3_^−^ by carbonic anhydrase (CA). Then, it is converted to malate by carboxylating phosphoenolpyruvate (PEP), a key component of the glycolysis. In a first step, PEP carboxylase (PEPC) converts PEP to oxaloacetate, after being activated by PEPC kinase (PPCK). In a second step, malate dehydrogenase (MDH) converts oxaloacetate to malate. Malate is then transported into the vacuole by 2 possible transporters, either a tonoplast dicarboxylate transporter or an aluminum-activated malate transporter, which are assisted by V-ATPase proton pumps. During the day, the accumulated malate becomes the main source of CO_2_ for photosynthesis. This allows the stomata to remain closed, which greatly enhances the water use efficiency of the plant. Malate is again transported out of the vacuole and reconverted to oxaloacetate by MDH, and then decarboxylated to PEP and CO_2_ by PEP carboxykinase (PEPCK). The CO_2_ will cycle through the Calvin cycle and generate sugars. GLR2.8, glutamate receptor 2.8; ABCC4, ABC transporter C 4; SDH, succinate dehydrogenase; XCT, XAP5 CIRCADIAN TIMEKEEPER; GI, protein GIGANTEA; RVE1, REVEILLE 1. **B)** Glycolysis, transitory starch, and sugar metabolism are tightly linked with the core CAM pathway as providers of starting materials such as PEP. During the day, CAM plants can store starch in the chloroplast and hexoses in the vacuole. In Bromeliaceae, the relative importance of soluble sugars versus starch as a source for PEP is variable across species (Christopher and Holtum 1998). At night, the stored starch and/or sugars are transported into the cytoplasm, converted to glucose or fructose, and broken down via the glycolysis to PEP. G6P, glucose-6-phosphate; GPT, glucose-6-phosphate/phosphate translocator; F6P, fructose-6-phosphate; PFK, phosphofructokinase; F1,6BP, fructose-1,6-biphosphate; DHAP, dihydroxyacetone phosphate; GAPDH, glyceraldehyde-3-phosphate dehydrogenase; 1,3BPG, 1,3-bisphosphoglyceric acid; PEP, phosphoenolpyruvate; SSI, starch synthase I; SUT2, sucrose transporter 2; ERD6, EARLY RESPONSE TO DEHYDRATION 6. For a detailed description and accompanying per-gene expression profiles, see Supplementary Fig. S9 and Supplementary SI Note S10.

Genes encoding core CAM enzymes phosphoenolpyruvate carboxylase (PEPC) and phosphoenolpyruvate carboxylase kinase (PEPC kinase, PPCK) displayed clear temporal expression cycling in *T. fasciculata* (Fig. 5C, Supplementary Fig. S9). *PPCK* also showed a night-time increase in expression in *T. leiboldiana* (Supplementary Fig. S10), albeit with a milder temporal effect, a phenomenon that has been documented before in C3-assigned *Tillandsia* (De La Harpe et al. 2020) and also in other C3-like species belonging to CAM- and C4-evolving lineages (Heyduk et al. 2019a; 2019b). Clustering analysis distributed DE genes across 7 clusters with sizes ranging from 209 to 38 genes (Supplementary Table S7). CAM-related genes were distributed across 6 of 7 clusters, highlighting the diversity of expression profiles associated with CAM (Supplementary Fig. S11). While core CAM genes (see Fig. 6) were mainly present in cluster 5, we found genes encoding malate transporters in cluster 1, circadian regulators in clusters 2 and 3, sugar transporters in clusters 3 and 6, and vacuolar transport regulators in clusters 2, 4, and 6. Cluster 7, though not containing any core CAM candidate genes, was enriched for salt and heat stress response and contained a gene encoding mitochondrial isocitrate dehydrogenase, which has been proposed as an alternative carbon fixator in CAM plants (Töpfer et al. 2020; Tay et al. 2021).

The expression curves of the respective clusters (Supplementary Fig. S11) demonstrate a complex web of expression changes between photosynthetic phenotypes. The most common expression change pattern among CAM-related genes is an overall increase in expression in the strong CAM plant (*T. fasciculata*), paired with increased diel cycling peaking in the early night (clusters 2, 5, and 6). This involved genes encoding enzymes of the night-time carbon fixating module of CAM such as PEPCK, PPCK, and MDH, enzymes involved in malate transport as V-ATPase and several glycolysis enzymes such as glucose-6-phosphate isomerase, aldolase, Ppi-dependent phosphofructokinase (PFK), and enolase (Fig. 6, Supplementary Fig. S9). Genes encoding enzymes of both soluble sugar transport (SUT2, ERD6, cluster 6) and starch metabolism (starch synthase I, α- and β-amylase and glucose-6-phosphate/phosphate translocator [GPT], clusters 5 and 6) showed overall upregulated and increased cycling expression curves in *T. fasciculata* compared to in *T. leiboldiana*, with highest activity in the late day. While the increased night-time expression of Ppi-dependent *PFK* suggests a primary role of soluble sugars as a night-time source for PEP (Carnal and Black 1989) in CAM *Tillandsia*, the simultaneously cycling expression patterns in starch metabolic enzyme genes point also at transitory starch as a potential source. Some CAM-related genes show increased expression in *T. leiboldiana*: namely genes encoding an aluminum-activated malate transporter (ALMT), the secondary vacuolar proton pump AVP1, which also displays a phase shift peaking later in the night in *T. fasciculata*, and 3 circadian clock regulators (LHY, GI and RVE1, cluster 1), which all show similar but reduced cycling patterns in *T. fasciculata* compared to *T. leiboldiana*. We also see a phase shift in the succinate dehydrogenase gene, peaking earlier in the night in *T fasciculata* versus *T. leiboldiana*'s early morning peak.

Overall, most CAM-related DE gene expression profiles align with the view that *T. fasciculata* is a constitutive CAM plant while *T. leiboldiana* performs a C3-like metabolism in normal conditions, though showing signs of very early CAM evolution. The difference in metabolism between both plants seems to be largely attained through regulatory rewiring of functional enzymes.

### Circadian clock-related motif enrichment in promoter sequences

We calculated the per-kb frequency of 4 known circadian clock-related motifs in the 2-kb upstream regions of identified DE genes, to further understand the role of circadian clock regulation in this set. We contrasted the frequencies of each motif in the set of DE genes against their frequencies in upstream regions of non-DE genes, and found that the Evening Element (EE) and CCA1-binding site (CBS) were the most enriched in this set with a frequency increase of 19% and 18%, respectively (Supplementary Table S8). The difference in median per-promotor count of these motifs was however not statistically significant (Supplementary Table S8, Supplementary Data Set 2). Among co-expression clusters, the changes in motif frequency compared to non-DE genes varied greatly. We find a significant increase in motif frequency in cluster 1 for the G-Box motif (82% increase), in cluster 3 for EE (207%) and in cluster 7 for CBS (43%).

We performed the same analysis on a set of *T. leiboldiana* genes that were temporally differentially expressed (see Supplementary SI Note S9). The upstream regions of these genes also showed a small but not statistically significant increase of 16% and 10% in EE and CBS frequency, respectively. The enrichment of circadian clock-related motifs in promotor regions of DE genes shows similarities between the 2 species, with comparable rates of frequency change for each specific element, though they are slightly larger in *T. fasciculata*.

When comparing the composition of circadian motifs in the upstream regions of core CAM genes and their homologs between species, we find a large diversity of motif presence among genes (Supplementary Table S9), yet homologs between species tend to share the same motifs. No circadian motif appears to be present in any homolog of *PEPC* except for a copy in *T. leiboldiana* which was not differentially expressed between species. On the other hand, the DE *PPCK* gene (*PPCK2)*, which encodes an important regulator of PEPC, contains several circadian motifs, and shows marked differences in its composition compared to the non-DE *PPCK gene* (*PPCK1*). *PPCK2* misses 2 G-Box sites compared to its homolog in *T. leiboldiana*.

### Genomic features of DE and CAM-related genes

#### Genomic distribution of DE genes is not associated with rearranged regions

Differentially expressed genes are present on all major scaffolds of both genome assemblies and the total number of DE genes per scaffold is positively correlated with the scaffold size (Kendall's correlation coefficient: 0.365 in *T. fasciculata* and 0.453 in *T. leiboldiana*, *P*-values < 0.011, Supplementary Data Set 2). Rearranged scaffolds in *T. leiboldiana* do not show a deviation in DE counts from other scaffolds relative to their size (Supplementary Fig. S12). The density of DE genes is slightly higher in *T. fasciculata* than in *T. leiboldiana* (1.47 vs. 0.93 DE genes per 1-kb window on average). On the other hand, the average proportion of genes that are DE per 1-kb window is higher in *T. leiboldiana* (3.3%) than in *T. fasciculata* (2.9%), indicating that DE genes are more often located in gene-sparse regions in *T. leiboldiana* than in *T. fasciculata*(Supplementary Fig. S13).

#### Differentially expressed genes belong more often to multicopy orthogroups

To investigate the consequences of gene family evolution on gene expression, we tested whether the proportion of multicopy orthogroups underlying DE genes was significantly elevated to that of the whole-genome set of orthogroups in both species (Fig. 5B, Supplementary SI Note S9). The 907 DE genes in *T. fasciculata* are found in 738 orthogroups (hereafter called DE orthogroups) containing a total of 2,141 and 910 genes in *T. fasciculata* and *T. leiboldiana*, respectively. Genes from multicopy orthogroups are more likely to be differentially expressed: while multicopy orthogroups account for 24% of all orthogroups in the genome, they represent 31% of DE orthogroups. This difference is primarily explained by a 3.2-fold increase in proportion of multicopy orthogroups with a larger family size in *T. fasciculata* than in *T. leiboldiana* in the subset of DE orthogroups compared to non-DE orthogroups (chi-square *P*-value < 2.2E^−16^, Supplementary Data Set 2).

Reciprocally, the DE analysis on the *T. leiboldiana* genome (Supplementary SI Note S9) resulted in 836 DE genes belonging to 714 orthogroups, of which 489 overlap with the DE orthogroups resulting from the analysis on the *T. fasciculata* genome. As in the analysis on the *T. fasciculata* genome, we find that orthogroups with a larger family size in *T. fasciculata*, but also in *T. leiboldiana* are enriched among DE orthogroups. Additionally, both analyses point at a significant enrichment for multicopy orthogroups with equal family sizes in both species, suggesting that older duplications preceding the split of *T. fasciculata* and *T. leiboldiana* also play a role in day-night regulatory evolution. This highlights the importance not only of novel, but also ancient variation in fueling trait evolution in *Tillandsia*.

Multicopy gene families are also enriched in a restricted subset of DE orthogroups related to CAM, starch metabolism, and gluconeogenesis (68 genes in 67 orthogroups), especially gene families with equal copy number and with copy number expansion in *T. fasciculata* (Fig. 5B, Supplementary Table S5). Importantly, expanded gene families in *T. leiboldiana* are not significantly enriched in this functional subset, showing that while the full set of DE orthogroups exhibit increased gene family dynamics in both lineages, CAM-related gene family expansion is only associated with *T. fasciculata* (constitutive CAM). This pattern is also reflected on the GO-term level, where enriched CAM-related biological functions appear disproportionally associated with gene family expansion in *T. fasciculata* (9 functions) than in *T. leiboldiana* (1 function). Functions associated with V-ATPase proton pumps especially tend to have larger gene family size in *T. fasciculata* than in *T. leiboldiana* (ATPase binding, proton-transporting ATPase activity).

CAM-related expanded gene families often show 1 highly expressed copy that performs diel cycling, while the other copies are lowly or not expressed (e.g. genes encoding SDH, glyceraldehyde-3-phosphate dehydrogenase (GAPDH), V-ATPase subunit H, Supplementary SI Note S10), however, several gene families show diel and/or elevated expression in *T. fasciculata* in 2 or more gene copies (genes encoding starch synthase, Ppi-dependent PFK and enolase, Supplementary SI Note S10). Both copies of *XCT* are expressed in *T. fasciculata*, though showing no diel cycling or increased expression compared to in *T. leiboldiana* (Fig. 5C), making the role of XCT in CAM photosynthesis unclear. The gene encoding V-ATPase subunit H has 8 copies in *T. fasciculata* and 3 in *T. leiboldiana*. While in both species, only 1 copy is highly expressed (with diel cycling peaking at night in *T. fasciculata*), the copies implemented for elevated expression in either species are not each other's orthologs (Supplementary SI Note S10), suggesting that different copies are recruited for the distinct photosynthetic phenotypes of these species. The other copies are lowly or not expressed. A gene family putatively encoding aquaporin PIP2-6 (OG0005047, Supplementary Fig. S14), which is involved in water regulation and follows a diel pattern in pineapple (*A. comosus*) (Zhu and Ming 2019), has an expanded gene family size in *T. leiboldiana*. While lowly expressed in *T. fasciculata*, 1 of the 2 gene copies shows strong diel expression in *T. leiboldiana*, with highest expression in the early night. This is another indication that an early, latent CAM cycle may be present in *T. leiboldiana*.

CAM-related gene families with duplications preceding the divergence of *T. leiboldiana* and *T. fasciculata* include genes encoding a malate dehydrogenase (MDH) with 2 copies in both species, where only 1 copy is highly expressed and cycling in *T. fasciculata*, and the core CAM enzyme phosphoenolpyruvate carboxylase (PEPC), which shares an ancient duplication among monocots (Deng et al. 2016) (Fig. 5C). The widely varying expression patterns of multicopy DE CAM-related families suggest a variety of mechanisms possibly contributing to CAM regulatory evolution: dosage changes (“more of the same”), subfunctionalization, and neofunctionalization.

#### Transcription factor gene family evolution and differential expression

Given the prominent role of gene expression regulation in modulating photosynthetic metabolism and the association of gene family expansion in CAM-related DE genes, we investigated whether transcription factor (TF) gene families have also undergone gene family evolution in *Tillandsia*. We identified 1,359 gene families containing either a known *A. comosus* TF gene or genes annotated with an InterPro domain characteristic of the largest known TF gene families. Compared to non-TF gene families, one-to-one single-copy families are significantly overrepresented in TF gene families (85.43%, chi-square *P*-value = 4.24E^−19^, Supplementary Data Set 2). Multicopy families and families unique to 1 species were all underrepresented among TF gene families (Supplementary Table S10).

Of the orthogroups belonging to identified TF gene families, 37 are differentially expressed in *T. fasciculata* and *T. leiboldiana*, and are found distributed across all 7 co-expression clusters. The proportion of one-to-one orthogroups in this subset of DE TF genes is slightly lower than that of the whole set of orthogroups in both species (Supplementary Table S10), while that of multicopy gene families is elevated, though not significantly enriched. Five DE TF gene orthogroups are expanded in *T. fasciculata*, including 1 *MYB-like*, 1 *FAR1-related*, 1 *C2H2*, and 2 *C3H* genes. Three DE TF gene orthogroups are expanded in *T. leiboldiana*: 1 *bZIP*, 1 *C2H2*, and 1 *ARF* gene. Lastly, 2 orthogroups are multicopy but equal in size between species, 1 *NF-YB* and 1 *TALE* gene. While we do witness an increase in gene family expansion in DE TF gene orthogroups, it has a reduced effect on this group of gene families compared to the overall set of DE genes and CAM-related DE genes.

#### Differentially expressed genes have more TE insertions

To investigate whether TE activity and differential gene expression are associated in *Tillandsia*, we tested whether TE insertions in introns and the 3-kb upstream regions of genes are significantly enriched in DE genes in both species. Both the presence of 1 or more TE insertions in a gene, as well as the average number of TE insertions per gene is higher across all genes of *T. leiboldiana* compared to the *T. fasciculata* gene set, which was expected given its larger proportion of repetitive content (see results, Genic, repetitive and GC content).

While in both genomes, the proportion of DE genes with 1 or more TE insertions is not significantly different to that of the full gene set, the average number of TE insertions per gene is significantly higher in DE compared to non-DE genes (Table 1).

**Table 1.** Statistical test results on TE insertions in DE versus non-DE genes, and in DE genes previously described as underlying CAM, glycolysis, or starch metabolism, versus all other genes

Total number of genes with 1 or more TE insertions						
	*T. fasciculata*			*T. leiboldiana*		
	All genes	DE genes	CAM-DE genes	All genes	DE genes	CAM-DE genes
All TEs (intronic only)	15,844 (50%)	473 (52%)	28 (41.2%)	10,348 (51%)	387 (54%)	21 (36.2%)^a^
All TEs	22,969 (90%)	725 (91%)	58 (85%)	18,512 (91%)	665 (92%)	50 (86%)
DNA transposons	16,857 (66%)	556 (70%)	45 (66%)	14,463 (71%)	547 (76%)^b^	42 (72%)
Helitrons	17,388 (69%)	561 (70%)	47 (69%)	14,701 (72%)	530 (74%)	38 (65%)
LTR-Copia	11,147 (44%)	353 (44%)	28 (41%)	10,487 (51%)	394 (55%)	31 (53%)
LTR-Gypsy	10,365 (41%)	341 (43%)	22 (32%)	5,399 (26%)	199 (28%)	10 (17%)
**Average and (median) TE insertion counts per gene**						
	***T. fasciculata***			***T. leiboldiana***		
	**Non-DE genes**	**DE genes**	**CAM genes**	**Non-DE genes**	**DE genes**	**CAM genes**
All TEs (intronic only)	2.90 (1)	3.71 (1)^a^	2.618 (0)	3.24 (1)	3.96 (1)^b^	2.862 (0)
All TEs	6.79 (5)	7.83 (6)^b^	6.32 (5)	7.62 (5)	8.75 (6)^c^	7.36 (5)
DNA transposons	2.15 (1)	2.56 (2)^c^	2.03 (1)	2.49 (2)	2.97 (2)^c^	2.77 (2)
Helitrons	1.97 (1)	2.27 (2)^c^	2.12 (1)	2.43 (2)	2.72 (2)^a^	2.12 (1)
LTR-Copia	1.00 (0)	0.95 (0)	0.87 (0)	1.22 (1)	1.45 (1)^b^	1.25 (1)
LTR-Gypsy	0.92 (0)	1.09 (0)	0.76 (0)	0.61 (0)	0.65 (0)	0.39 (0)

TE insertions were counted in intronic + 3-kb upstream regions, but insertions in introns only are also shown. ^a^*P* > 0.05, ^b^*P* > 0.01, ^c^*P* > 10–3.

On the other hand, TE insertion rates in DE genes related to CAM, starch metabolism, and glycolysis/gluconeogenesis do not significantly differ from background rates in both genomes, though they are slightly reduced (Table 1). However, the proportion of CAM-related DE genes with an intronic TE insertion is larger in *T. fasciculata* (41.2%) than in *T. leiboldiana* (36.2%), despite *T. leiboldiana*'s generally elevated intronic TE insertion rate. This pattern is not discernible when including TE insertions in the 3-kb upstream region of genes.

When studying genic TE insertions across 4 separate TE classes, we recover a similar trend among all categories as observed across all TEs. Insertion rates around genes are the highest for DNA transposons in both species, but the TE class that is most often present around a gene is Helitrons, which occur in 69% and 72% of genes in *T. fasciculata* and *T. leiboldiana*, respectively (Table 1). This contrasts with the small proportion of the whole genome that is covered by Helitrons—only 5.86% and 3.7% in *T. fasciculata* and *T. leiboldiana*, respectively (Supplementary Table S3). LTRs, while covering the largest proportion of the genome in both species, are the least present and show the lowest insertion rates around genes in both species.

Nine DE genes related to CAM, starch, and gluconeogenesis display more than twice the number of TE insertions as the genome-wide average in *T. fasciculata*. This includes genes encoding a V-type proton ATPase subunit H (vacuolar transport and acidification), an aluminum-activated malate transporter, an ABC transporter C family member 4 (stomatal opening and circadian rhythm) and a mitochondrial isocitrate dehydrogenase subunit (Supplementary Table S11), which all had more TE insertions than their orthologs in *T. leiboldiana*. On the other hand, 6 DE genes of interest had elevated TE insertion rates in *T. leiboldiana*, including genes encoding a vacuolar acid invertase (sugar metabolism) and a sugar transporter ERD6-like. Four of these genes showed high amounts of TE insertions in both genomes, such as a gene encoding a glucose-1-phosphate adenylyltransferase subunit (starch synthesis), a V-type proton ATPase subunit C, and circadian clock regulator GIGANTEA (Dalchau et al. 2011), which also plays a role in stomatal opening (Ando et al. 2013).

## Discussion

The sources of variation fueling trait evolution in rapid radiations have been a long-standing topic in evolutionary biology (Simpson 1953), and our understanding of how complex traits such as CAM evolve repeatedly is still incomplete. By showcasing a broad range of photosynthetic phenotypes and repeated evolution of constitutive CAM, the subgenus *Tillandsia* provides an excellent opportunity to study CAM evolution. The recent divergence between members of *Tillandsia* allows us to pinpoint the necessary evolutionary changes to evolve a constitutive CAM phenotype. By integrating comparative genomics using de novo assemblies and in-depth gene expression analyses of 2 closely related *Tillandsia* species representing one of the most distinct photosynthetic phenotypes within the clade, we found support for regulatory evolution and gene family expansion as major features of CAM evolution (Fig. 6).

Our metabolic analyses of night-time malate accumulation and gene expression analyses provide a much more detailed understanding of the photosynthetic phenotypes present in the subgenus than the previously reported carbon isotope measurements, which do not accurately reflect all stages of the CAM continuum. For example, weak CAM phenotypes have been reported in Bromeliaceae for species with carbon isotope ratios falling in the C3 range (−26.5) (Pierce et al. 2002), indicating that the photosynthetic metabolism of *T. leiboldiana* could be different from C3 *sensu stricto* as its carbon isotope measurements suggest. Our timewise malate measurements show distinct fluxes for *T. fasciculata* and *T. leiboldiana*, with constitutive accumulation of night-time malate in *T. fasciculata* which is absent in *T. leiboldiana*. On the other hand, *T. leiboldiana* displays CAM-like temporal expression profiles for certain enzymes, such as PEPC kinase and Aquaporin PIP-6, and shares circadian clock-related cis-elements in the promotor regions of CAM homologs with *T. fasciculata*. It has been suggested that repeated evolution of CAM (similar to C4) may be facilitated in lineages where C3 species already display increased or CAM-like expression of core genes (Kajala et al. 2012; Heyduk et al. 2019a, 2019b). However, while a CAM cycle is seemingly not being expressed in *T. leiboldiana* under normal circumstances, we cannot exclude that a latent CAM cycle could become activated under certain conditions, for example under drought stress. In that case, *T. leiboldiana* would rather be at the very early stages of CAM evolution than a “pre-adapted” C3 plant (De La Harpe et al. 2020). We hope that future studies will investigate the drought response of *T. leiboldiana* to better understand its exact position in the CAM continuum.

On the other hand, even if *T. leiboldiana* and potentially all subgenus *Tillandsia* species previously labeled as C3 represent in fact the early stages of the CAM continuum, our analyses show widely distinct photosynthetic phenotypes within the radiation which required divergent evolution. Therefore, while this study may underestimate the total number of evolutionary changes needed to establish constitutive CAM from C3 *sensu stricto*, it highlights the evolutionary drivers underlying the least understood section of the CAM continuum: from early CAM to constitutive CAM.

Differences between the 2 genomes related to CAM evolution can be primarily found on the regulatory level, with CAM-related genes showing temporal differential expression between species across a 24-h period. These reveal a complex web of underlying expression changes, as they are distributed over all inferred co-expression clusters (Supplementary Fig. S11). Together with the diversity of circadian clock-related motif composition in promoter sequences (Supplementary Table S8), this finding emphasizes the lack of a master regulator and a clear overall direction of expression changes underlying CAM (Wickell et al. 2021; Heyduk et al. 2022).

Gene family expansion has been previously observed in CAM lineages (Silvera et al. 2010; Cai et al. 2015) and suggested as a driver of CAM evolution (Silvera et al. 2014). We witnessed an increased number of genes belonging to multicopy families in *T. fasciculata* than in *T. leiboldiana*, consistent with a net higher rate of gene duplication in this species than in *T. leiboldiana*, as previously reported by De La Harpe et al. (2020). Strikingly, both the total subset of differentially expressed genes and a more stringent group of CAM-related DE genes was significantly enriched for gene families that have expanded in *T. fasciculata* (constitutive CAM). CAM-related functions that were enriched in DE genes show a disproportionate bias toward gene family expansion in *T. fasciculata* (circadian rhythm, vacuolar ATPase activity, tricarboxylic acid cycle and starch metabolism) compared to *T. leiboldiana* (glycolysis) (Fig. 5A). Gene duplications preceding the split of *T. fasciculata* and *T. leiboldiana* are also significantly associated with day-night expression differences in CAM-related genes (Fig. 5B), suggesting that older, already existing gene duplications may also be recruited in CAM evolution, alongside novel duplications.

The expression curves of DE multicopy gene families with a potential link to CAM reveal a multitude of expression behaviors (e.g. Fig. 5C), which supports that complex regulatory evolution on the transcriptional level underlies CAM evolution. Our findings suggest that gene family evolution played a substantial role in modulating regulatory changes underlying the evolution toward constitutive CAM in *Tillandsia*. As gene family expansion leads to increased redundancy, selection on individual gene copies and their expression relaxes, facilitating the assimilation of a constitutively expressed CAM expression profile (Ohno 1970).

Another potential driver of trait evolution is TE insertion, though its role in CAM evolution in *Tillandsia* remains unclear. TE insertions are overall less common in CAM-related DE genes compared to all genes in both genomes, suggesting a selection pressure against TE insertions around these genes. However, the proportion of CAM-related DE genes with intronic TE insertions is greater in *T. fasciculata* than in *T. leiboldiana*, despite the overall higher genic TE insertion rate in *T. leiboldiana*. This suggests that the pressure to maintain CAM-related genes TE-free is reduced in the constitutive CAM lineage relative to *T. leiboldiana*. We detect 9 and 6 CAM-related DE genes with more than twice the whole-genome average TE insertion count in *T. fasciculata* and *T. leiboldiana*, respectively, of which 4 are single-copy orthologs shared between species. The high degree of sharedness of TE-rich DE genes between species rather suggests that TE insertions are not a major driver of CAM-related gene expression changes. In fact, genes with exceptionally high insertion rates in *T. fasciculata* tend to show reduced expression (ALMT, V-ATPase subunit H copy Tfasc_v1.24696, ABCC4, Supplementary SI Note S10). Instead, the larger proportion of CAM-related DE genes with 1 or more TE insertions in *T. fasciculata* may be a consequence of higher rates of gene family expansion and eventual pseudogenization of redundant copies.

Candidate genes under positive selection underlie a broad array of functions, but had no immediate link to CAM photosynthesis. While the study of adaptive sequence evolution would greatly benefit from a broader sampling across *Tillandsia*, the lack of overlap between regulatory and adaptive sequence evolution is in line with previously proposed mechanisms of CAM evolution largely relying on regulatory changes in other systems (Deng et al. 2016). A small number of cases of convergent and adaptive sequence evolution between distantly related CAM and C3 species have been described (Yang et al. 2017), though no overlap was found between convergence in expression and sequence evolution. Our study suggests that while on larger evolutionary scales adaptive sequence evolution may play an important role, distinct photosynthetic phenotypes between closely related species may be achieved primarily with gene expression changes, or may be especially relevant between the transition of C3 sensu stricto to the CAM continuum.

Though we observe a karyotype difference of 6 chromosome pairs between *T. fasciculata* and *T. leiboldiana* and we identified 1 fusion in the *T. leiboldiana* assembly, along with 2 reciprocal translocations, we did not find detectable consequences of large-scale rearrangements for either functional diversification or adaptation in *Tillandsia*, unlike other studies (Davey et al. 2016; Cicconardi et al. 2021) (Fig. 4B, Supplementary Figs. S7 and S8, but see Supplementary SI Note S5). However, due to the remaining fragmentation of the *T. leiboldiana* genome, it is likely that we were not able to describe all rearrangements, and we hope that future endeavors will improve the genome assembly and make a more in-depth study of the role of large-scale rearrangements in the evolution of species barriers and/or the evolution of other key innovation traits in *Tillandsia* possible.

Our analyses reveal genomic changes of all scales between 2 members of an adaptive radiation representing a recent shift to constitutive CAM. However, in this recent shift between closely related species, differences in photosynthetic metabolism are brought about largely by temporal expression changes enabled by both existing and *de novo* gene duplication, rather than adaptive sequence evolution of existing gene copies, which may play a role at later stages of divergence. Large-scale rearrangements observed so far seem unlinked from functional divergence, more likely affecting reproductive isolation (Faria and Navarro 2010; de Vos et al. 2020), and need further study. Our findings support an important role for gene family expansion in generating novel variation that fuels the evolution of the CAM continuum.

The 2 *de novo* assemblies presented in this study are, to our knowledge, the first tillandsioid and fourth bromeliad genomes published so far. Despite both genomes exhibiting one of the highest TE contents reported to date for a non-polyploid plant species (Pedro et al. 2021), the joint use of long-read sequencing and chromatin conformation capture successfully led to highly contiguous assemblies with high-quality gene sets (Supplementary SI Note S11). Along with other recently developed resources for Bromeliaceae (Liu et al. 2021; Yardeni et al. 2021), these genomes will be crucial in future investigations of this highly diverse and species-rich plant family, and in further studies of CAM evolution.

## Materials and methods

### Plant material collection

This study performed genomic and transcriptomic analyses on accessions of the giant air plant (*T. fasciculata*) and of *T. leiboldiana*. A single genotype per species was sampled from the collection at the Botanical Garden of the University of Vienna for the purpose of *de novo* genome assembly. Leaf material was obtained from an adult plant mounted on a plastic tube (not potted or planted in soil) for both species. For transcriptomic analyses, leaf material was obtained from 5 additional genotypes per species. These were separate, adult plants that were either mounted on a plastic tube or on a metal bracket lodged on top of soil (the plants remained unpotted). All plants at the Botanical Garden of the University of Vienna are maintained in glasshouses under natural light conditions. Details on the origin, sampling locality, and collector of each plant can be found in Supplementary Table S1.

### Flow cytometry and cytogenetic experiments

#### Genome size measurements

Approximately 25 mg of fresh leaf material was co-chopped according to the chopping method of Galbraith et al. (1983) together with an appropriate reference standard (*Solanum pseudocapsicum*, 1.295 pg/1C) (Temsch 2010; Temsch et al. 2022) in Otto´s I buffer (Otto et al. 1981). After filtration through a 30 *µ*m nylon mesh (Saatilene Hitech, Sericol GmbH, Germany) and incubation with RNase A (0.15 mg/mL, Sigma-Aldrich, USA) at 37 °C, Otto´s II buffer (Otto et al. 1981) including propidium iodide (PI, 50 mg/L, AppliChem, Germany) was added. Staining took place in the refrigerator for between 1 h and overnight. Measurement was conducted on a CyFlow ML or a CyFlow Space flow cytometer (Partec/Sysmex, Germany) both equipped with a green laser (532 nm, 100 mW, Cobolt AB, Sweden). The fluorescence intensity (FI) of 10,000 particles was measured per preparation and the 1C-value calculation for each sample followed the equation: 1C_Obj_ = (FI peak mean_G1 Obj_/FI peak mean_G1 Std_) × 1C_Std_.

#### Karyotyping

Actively growing root meristems of genome assembly accessions (see Supplementary Table S1) were harvested and pretreated with 8-hydroxyquinoline for 2 h at room temperature and 2 h at 4 °C. The roots were then fixed in Carnoy's fixative (3:1 ethanol:glacial acetic acid) for 24 h at room temperature and stored −20 °C until use. Chromosome preparations were made after enzymatic digestion of fixed root meristems as described in Jang and Weiss-Schneeweiss (2015). Chromosomes and nuclei were stained with 2 ng/*µ*L DAPI (4′,6-diamidino-2-2phenylindole) in Vectashield antifade medium (Vector Laboratories, Burlingame, CA, USA). Preparations were analyzed with an Axiolmager M2 epifluorescent microscope (Carl Zeiss) and images were captured with a CCD camera using AxioVision 4.8 software (Carl Zeiss). Chromosome number was established based on analyses of several preparations and at least 5 intact chromosome spreads. Selected images were contrasted using Corel PhotoPaint X8 with only those functions that applied equally to all pixels of the image and were then used to prepare karyotypes.

### Genome assembly

#### Plant material selection and sequencing

Genome assemblies were constructed from the plant material of 1 accession per species (see Supplementary Table S1). The accessions were placed in a dark room for a week to minimize chloroplast activity and recruitment, after which the youngest leaves were collected and flash frozen with liquid nitrogen. High molecular weight extraction for ultra-long reads, SMRTbell library preparation, and PacBio Sequel sequencing was performed by Dovetail Genomics (now Cantata Bio). Dovetail Genomics also prepared Chicago (Putnam et al. 2016) and Hi-C (Lieberman-Aiden et al. 2009) libraries which were sequenced as paired-end 150-bp reads on an Illumina HiSeq X instrument. Additional DNA libraries were prepared for polishing purposes using Illumina's TruSeq PCR-free kit, which were sequenced on a HiSeq2500 as paired-end 125-bp reads at the Vienna BioCenter Core Facilities (VBCF), Austria.

RNA-seq data of *T. fasciculata* used for gene annotation were sampled, sequenced, and analyzed in De La Harpe et al. 2020 under SRA BioProject PRJNA649109. For gene annotation of *T. leiboldiana*, we made use of RNA-seq data obtained during a similar experiment, where plants were kept under greenhouse conditions and sampled every 12 h in a 24-h cycle. Importantly, while the *T. fasciculata* RNA-seq data set contained 3 different genotypes, only clonal accessions were used in the *T. leiboldiana* experiment. For *T. leiboldiana*, total RNA was extracted using a QIAGEN RNeasy Mini Kit, and poly-A capture was performed at the Vienna Biocenter Core Facilities (VBCF) using a NEBNext kit to produce a stranded mRNA library. This library was sequenced on a NovaSeq SP as 150-bp paired-end reads.

For both species, sequencing data from different time points and accessions were merged into 1 file for the purpose of gene annotation. Before mapping, the data were quality-trimmed using AdapterRemoval (Schubert et al. 2016) with default options (--trimns, –trimqualities). We allowed for overlapping pairs to be collapsed into longer reads.

#### First draft assembly and polishing

We constructed a draft assembly using long-read PacBio data with CANU v1.8 (Koren et al. 2017) for both species. To mitigate the effects of a relatively low average PacBio coverage (33×), we ran 2 rounds of read error correction with high sensitivity settings (corMhapSensitivity=high corMinCoverage=0 corOutCoverage=200) for *T. fasciculata*. Additionally, we applied high heterozygosity (correctedErrorRate=0.105) settings, since k-mer and window-based analyses pointed at an elevated heterozygosity in this species (see Supplementary SI Note S2, Supplementary Figs. S3 and S15), and memory optimization settings (corMhapFilterThreshold=0.0000000002 corMhapOptions=“ --repeat-idf-scale 50” mhapMemory=60g mhapBlockSize=500).

Given that the coverage of *T. leiboldiana* PacBio averaged 40×, we limited error correction for this species to only 1 round. CANU was run with additional settings accommodating for high frequency repeats (ovlMerThreshold=500) and high sensitivity settings as mentioned above.

To minimize the retention of heterozygous sequences as haplotigs in *T. fasciculata* (see Supplementary SI Note S2), we reassigned allelic contigs using the pipeline Purge Haplotigs (Roach et al. 2018). Raw PacBio data were mapped to the draft assembly produced in the previous step with minimap2 (Li 2018), before using the Purge Haplotigs pipeline.

Since the size of the *T. leiboldiana* draft assembly indicates, together with previous analyses, that this species is largely homozygous (Supplementary SI Note S2), we did not include a PurgeHaplotigs step. However, we did make use of the higher average coverage of the *T. leiboldiana* PacBio data to polish the assembly with 2 rounds of PBMM v.1.0 and Arrow v2.3.3 (Pacific Biosciences).

#### Scaffolding and final polishing

Scaffolding of both assemblies was performed in-house by Dovetail Genomics using Chicago and Hi-C data and the HiRise scaffolding pipeline (Putnam et al. 2016). To increase base quality and correct indel errors, we ran additional rounds of polishing with high-coverage Illumina data (see above, Photosynthetic phenotypes of *T. fasciculata* and *T. leiboldiana*) using Pilon v1.22 (Walker et al. 2014). The Illumina data were aligned to the scaffolded assembly using BWA-MEM (Li 2013), and then Pilon was run on these alignments. We evaluated the result of each round using BUSCO v.3 (Waterhouse et al. 2018) with the Liliopsida odb9 library and proceeded with the best version. For *T. fasciculata*, polishing was performed twice, fixing SNPs, and indels. We did not fix small structural variation in this genome due to the relatively low coverage (35×) of Illumina data. For *T. leiboldiana*, 1 round of polishing on all fixes (SNPs, indels, and small structural variants) resulted in the highest BUSCO scores.

### Annotation

#### TE annotation and repeat masking

De novo TE annotation of both genome assemblies was performed with EDTA v.1.8.5 (Ou et al. 2019) with option –sensitive. To filter out genes that have been wrongly assigned as TEs, pineapple (*A. comosus*) coding sequences (Ming et al. 2015) were used in the final steps of EDTA.

Using the species-specific TE library obtained from EDTA, we masked both genomes using RepeatMasker v.4.0.7 (Smit et al. 2013-2015). Importantly, we excluded all TE annotations marked as “unknown” for masking to prevent potentially genic regions flagged as TEs to be masked during annotation. The search engine was set to NCBI (-e ncbi) and simple and low-complexity repeats were left unmasked (-nolow). We produced both hard-masked and soft-masked (--xsmall) genomes.

#### Transcriptome assembly

We constructed transcriptome assemblies for both species using Trinity de novo assembler v.2.4.8. (Grabherr et al. 2011) using default parameters starting from the raw mRNA-seq data. These were evaluated with BUSCO. Additionally, before feeding the transcriptome assemblies to the gene annotation pipeline, we ran a round of masking of interspersed repeats to avoid an overestimation of gene models due to the presence of active transposases in the RNA-seq data.

#### Gene prediction and functional annotation

Gene models were constructed using a combination of BRAKER v.2.1.5 (Hoff et al. 2019) and MAKER2 v.2.31.11 (Campbell et al. 2014). Starting with BRAKER, we obtained splicing information from RNA-seq alignments to the masked genome as extrinsic evidence using the *bam2hints* script of AUGUSTUS v.3.3.3 (Stanke et al. 2008). A second source of extrinsic evidence for BRAKER was single-copy protein sequences predicted by BUSCO when run on the masked genomes in genome mode with option --long. Predictions made by BRAKER were evaluated with BUSCO and with RNA-seq alignments.

Subsequently, we built our final gene predictions using MAKER2. As evidence, we used (i) the gene models predicted by BRAKER, (ii) a transcriptome assembly of each respective species (see above, First draft assembly and polishing), (iii) a protein sequence database containing proteins of 2 pineapple varieties—*A. comosus comosus* (F153) (Ming et al. 2015) and *A. comosus bracteatus* (CB5) (Chen et al. 2019)—and manually curated SwissProt proteins from monocot species (64,748 sequences in total), and (iv) a GFF file of complex repeats obtained from the masked genome (see above, Plant material selection and sequencing) and an extended repeat library containing both the EDTA-produced *Tillandsia*-specific repeats and the monocot repeat library from RepBase (7,857 sequences in total). By only providing masking information of complex repeats and setting the model organism to “simple” in the repeat masking options, hard-masking in MAKER2 was limited to complex repeats while simple repeats were soft-masked, which makes these available for gene prediction. MAKER2 predicts genes both ab initio and based on the given evidence using AUGUSTUS.

We evaluated the resulting set of predicted gene models by mapping the RNA-seq data (Photosynthetic phenotypes of *T. fasciculata* and *T. leiboldiana*) back to both the transcript and full gene model sequences and running BUSCO in transcriptome mode. We also calculated the proportion of masked content in these gene models to ascertain that MAKER2 had not predicted TEs as genes. A second run of MAKER, which included training AUGUSTUS based on the predicted models from the first round, resulted in lower BUSCO scores and was not further used. We functionally annotated the final set of gene models in Blast2Go v.5.2.5 (Götz et al. 2008) using the Viridiplantae database.

### Inferring gene orthology

Orthology between gene models of *T. fasciculata*, *T. leiboldiana*, and *A. comosus* was inferred using Orthofinder v.2.4.0 (Emms and Kelly 2019). Protein sequences produced by MAKER2 of inferred gene models were used for *T. fasciculata* and *T. leiboldiana*. For *A. comosus*, the publicly available gene models of F153 were used. The full Orthofinder pipeline was run without additional settings. Counts per orthogroup and the individual genes belonging to each orthogroup were extracted from the output file Phylogenetic_Hierarchical_Orthogroups/N0.tsv.

Orthofinder was run a second time on gene models present only on main contigs (see Results). For each gene model, the longest isoform was selected, and gene models with protein sequences shorter than 40 amino acids were removed. This resulted in 27,024, 30,091, and 31,194 input sequences for *A. comosus*, *T. fasciculata*, and *T. leiboldiana*, respectively. Then, the steps mentioned above were repeated.

### Gene model assessment and curation

Gene model sets were assessed and curated using several criteria. Gene models with annotations indicating a repetitive nature (transposons and viral sequences) together with all their orthologs were marked with “NO_ORTHOLOGY” in the GFF file and excluded from downstream analyses. Using the per-exon expression data obtained in our mRNA-seq experiment (see below, *RNA-seq experiment*) and information gathered on the length of the CDS and the presence/absence of a start and stop codon, we further classified our gene models into ROBUST and NOT-ROBUST categories. A gene model was considered ROBUST (i) if all exons are expressed or, (ii) if both start and stop codons are present and the CDS has a minimum length of 50 amino acids.

### Analyzing TE class abundances

By rerunning EDTA with step --anno, we obtained TE abundances and detailed annotation of repetitive content for the whole assembly. Per-contig abundances of each class were calculated with a custom Python script (available at https://github.com/cgrootcrego/Tillandsia_Genomes). Using this curated TE library, the assemblies were masked again with RepeatMasker for downstream analyses. The resulting TE class abundances reported by RepeatMasker were then compared between species and reported.

### Spatial distribution of repetitive, genic, and GC contents

The spatial distribution of genes, TEs, and GC content as shown in Fig. 3A, was analyzed on a per-window basis, using windows of 1 Mb. Gene counts were quantified as the number of genes starting in every window, based on genes with assigned orthology, including both single and multicopy gene models. Repetitive content was measured as the proportion of masked bases in each window, stemming from the hard-masked assembly using the curated TE library. Per-window gene counts and proportion of repetitive bases was then visualized using the R package circlize (Gu et al. 2014). GC content was calculated as the proportion of G and C bases per 1-Mb windows. Correlation between genic, repetitive, and GC contents was calculated and tested for significance using the Kendall Rank Correlation Coefficient, after testing for normality using the Shapiro–Wilk test.

Repetitive, GC, and gene contents as shown in Fig. 3B was estimated directly from the soft-masked reference genomes using 100 kb nonoverlapping sliding windows as described in Leroy et al. (2021). TE content corresponds to the proportion of soft-masked positions per window. For the *Tillandsia* genomes, the curated TE library (see above, TE annotation and Repeat Masking) was used as a basis for soft-masking in RepeatMasker. For *A*. *comosus*, a soft-masked version of the genome was obtained from NCBI (https://www.ncbi.nlm.nih.gov/datasets/genome/GCA_902162155.2/). As compared to the version of Leroy et al. 2021, this script was modified to estimate GC content in repetitive regions (soft-masked regions only). In addition to this, we estimated the genic fraction by considering the total number of genomic positions falling in genes based on the GFF files (feature = “gene”) divided by the size of the window (100 kb). This estimate was derived for the same window boundaries as used for GC and TE contents to be able to compare all statistics. The relative per-window proportion of genic bases corresponding to non-robust genes (see above, Gene model assessment and curation) was also estimated by dividing the number of non-robust gene positions with the total number of gene positions.

### Synteny between *T. fasciculata* and *T. leiboldiana*

Synteny was inferred with GENESPACE v.0.8.5 (Lovell et al. 2022), using orthology information obtained with Orthofinder of the gene models from *A. comosus*, *T. fasciculata*, and *T. leiboldiana*. This provided a first visual graphical to detect large-scale rearrangements. We used GENESPACE with default parameters, except that we generated the syntenic map (riparian plot) using minGenes2plot=200. Other methods have also been used to confirm the chromosomal rearrangements and to identify the genomic breakpoints more precisely (see Supplementary SI Note S5).

### Gene family evolution

#### Family size correction

Gene counts per orthogroup were evaluated using per-gene mean coverage to detect co-assembled heterozygous gene sequences that may have escaped Purge Haplotigs in the assembly step. To do this, whole-genome Illumina reads of both species (see Materials and methods, Plant material selection and sequencing) were aligned to their respective assemblies using Bowtie2 v.2.4.4. (Langmead and Salzberg 2012) with the very-sensitive-local option. Bowtie2 specifically assigns multi-mapping reads randomly, allowing the detection of artificial gene models thanks to a decreased overall coverage across the orthogroup, as reads from 1 biological copy would be randomly distributed over 2 or more locations in the genome. Per-base coverage in genic regions was calculated using *samtools depth* and a bed-file specifying all locations of orthologous genes. We then calculated the average coverage per orthologous gene.

The distribution of per-gene mean coverage in each species’ gene model set was then visualized using ggplot2 (Wickham 2016) for different categories of genes: single-copy (only 1 gene model assigned to the orthogroup in the species investigated), multicopy (more than 1 gene assigned to the orthogroup in the species investigated), ancestral single-copy (only 1 gene model assigned to the orthogroup in all species used in the orthology analysis), ancestral multicopy (multiple gene model assigned to the orthogroup in all species used in the orthology analysis and the number of gene models assigned is equal across species), and unique multicopy (more than 1 gene assigned to the orthogroup in the species investigated and no genes assigned to the orthogroup in other species). This revealed that, while most categories of genes had a unimodal distribution centered around the average coverage across the genome, multicopy and unique multicopy families showed a bimodal or expanded distribution, especially in *T. fasciculata* (Supplementary Fig. S16). This points at the presence of genes with multiple alleles per gene in the annotation. Hence, gene count sizes per orthogroup and species were corrected by the ratio of the total coverage across all genes of 1 species in the orthogroup and the expected coverage, which was calculated as the product of the total number of genes in the orthogroup and the average coverage of single-copy genes in that species.

Size corrections were only applied on orthogroups containing multicopy genes. Plastid and mitochondrial genes were excluded from this analysis. We detected plastid genes with BLASTn against the *A. comosus* chloroplast sequence and the *Oryza* IRSGP-1 mitochondrial sequence. Additionally, all genes annotated as “ribosomal” were also excluded from the downstream gene family evolution analyses.

Originally, 9,210 genes in *T. fasciculata* and 6,257 genes in *T. leiboldiana* were assigned to orthogroups with multiple gene copies in at least 1 species. After correcting orthogroup sizes by coverage, we retained 6,261 and 4,693 gene models, respectively (Supplementary Table S4).

#### Analysis of multicopy orthogroups

The distribution of gene counts per multicopy orthogroup was compared between *T. fasciculata* and *T. leiboldiana* with a nonparametric test (Mann–Whitney *U*). Using the log-ratio of per-species gene count, we investigated which gene families experienced large changes in gene count compared to the background (Supplementary SI Note S6).

Functional characterization of multicopy families was done with a GO-term enrichment analysis of the underlying genes using the Fisher's exact test in TopGo (Alexa and Rahnenführer 2006). Enrichment analyses were done on all genes belonging to multicopy orthogroups, on a subset of genes belonging to families that are larger in *T. fasciculata* and on a subset of genes belonging to families that are larger in *T. leiboldiana*. The top 100 significantly enriched GO terms were then evaluated. GO terms putatively associated with key innovation traits were used to list multicopy gene families of interest.

Additionally, we searched for specific genes that are known to underlie CAM evolution in these multicopy gene families. The IDs of candidate pineapple genes for CAM were obtained from Yardeni et al. (2021) who compiled extensive lists of genes from a diverse set of studies. For CAM, we considered all genes listed in Supplementary Table S1 in this study under the categories “Differentially expressed in CAM/C3 experiment” (186 genes) (De La Harpe et al. 2020), “Positive selection in CAM/C3 shifts” (22) (De La Harpe et al. 2020), gene families associated with “CAM/C3” (79) (De La Harpe et al. 2020), “CAM-related *A. comosus*” (29) (Ming et al. 2015), “stomatal function” (48) (Christin et al. 2014), “aquaporin regulation” (24) (Vera-Estrella et al. 2012), “drought resistance” (61) (Xiao et al. 2007), “circadian metabolism” (47) (Wai et al. 2017), “malate transferase” (28) (Cosentino et al. 2013), and “circadian clock” (3) (McClung 2006), resulting in a total of 527 genes. A separate list was made for gluconeogenesis and starch metabolism genes (288 genes) (Cushman et al. 2008). After obtaining these lists of pineapple gene IDs, we searched for their orthologs in *T. fasciculata* and *T. leiboldiana*, and investigated their presence in multicopy gene families.

### *d*_N_/*d*_S_ analysis

#### On single-copy orthologous pairs

One-to-one orthologous genes were subjected to a test of positive selection using the non-synonymous to synonymous substitution ratio (*ω* = *d*_N_/*d*_S_). Gene pairs where both genes were incomplete (missing start and/or stop codon) or where the difference in total length was more than 20% of the length of either gene were removed. We performed codon-aware alignments using the alignSequences program from MACSE v.2.05 (Ranwez et al. 2018) with options -local_realign_init 1 -local_realign_dec 1 for optimization. Pairwise *d*_N_/*d*_S_ ratios were estimated with the codeML function of PAML v.4.9. (Yang 2007). Using a single-ratio model across sites and branches (Nssites = 0, model = 0), we tested for a fixed *ω* = 1 as null hypothesis, against an unfixed *ω* as the alternative hypothesis. Automatization of codeML was achieved with a modified script from AlignmentProcessor (https://github.com/WilsonSayresLab/AlignmentProcessor/). The results of codeML under both the null and alternative model were compiled and significance of the result was calculated with the likelihood-ratio test (Wong et al. 2004). Multiple-testing correction was applied with the Benjamini–Hochberg method and an FDR threshold of 0.05. Orthologous gene pairs with a *d*_N_/*d*_S_ ratio larger than 1 and an adjusted *P*-value under 0.05 were considered candidate genes under divergent selection.

The *d*_N_/*d*_S_ values of all orthologous gene pairs with 5 or more variant sites in the MACSE alignment were used to obtain per-scaffold distributions of *d*_N_/*d*_S_ values in both genomes. We visualized *d*_N_/*d*_S_ distributions of all main scaffolds in both assemblies with boxplots and used density plots to visualize the *d*_N_/*d*_S_ distribution in rearranged chromosomes compared to all non-rearranged chromosomes. To test whether these distributions were significantly different, we ran a nonparametric test (Mann–Whitney *U*) between the distribution of each single rearranged chromosome and that of all non-rearranged chromosomes in each assembly.

#### On duplicated orthogroups

We also performed tests of selection using *d*_N_/*d*_S_ on all orthogroups that consisted of a single gene in *A. comosus* and a duplicated gene in either *T. leiboldiana* (1:1:2) or *T. fasciculata* (1:2:1). Only orthogroups that maintained this conformation after size correction were used in this analysis. Pairwise alignments were performed between the ortholog of 1 species and either paralog of the other species using MACSE. Then, *ω* was estimated in the same way as mentioned above.

### RNA-seq experiment capturing photosynthetic phenotypes and expression

#### Experiment setup and sampling

To capture gene expression patterns related to CAM, we designed an RNA-seq experiment where individuals of *T. fasciculata* and *T. leiboldiana* were sampled at 6 time points throughout a 24-h cycle. Six plants of each species were placed in a PERCIVAL climatic cabinet at 22 °C and a relative humidity (rH) of 68% for 4 wk, with a 12-h light cycle. Light was provided by fluorescent lamps with a spectrum ranging from 400 to 700 nm. The light intensity was set at 124 *µ*mol/m^2^s. The plants were acclimated to these conditions for 4 wk prior to sampling; during these 4 wk they were watered every second day.

Leaf material from each plant was sampled every 4 h in a 24-h cycle starting 1 h after lights went off. One leaf was pulled out of the base at each time point without cutting. The base and tip of the leaf were then removed, and the middle of the leaf immediately placed in liquid nitrogen, then stored at −80 °C.

#### Targeted metabolite analyses

To corroborate the photosynthetic phenotypes of *T. fasciculata* and *T. leiboldiana*, we measured malate abundances in the leaf throughout a 24-h cycle. An approximate amount of 20 mg of frozen leaf material collected at 6 time points during the above-mentioned experiment was collected and ground to a powder with a TissueLyser and metal beads. Subsequent steps were performed at the Vienna Metabolomics Center (VIME, Department of Ecogenomics and Systems Biology, Vienna, Austria).

Polar metabolites were extracted in 3 randomized batches by modifying the procedure of Weckwerth et al. (2004). A weighed amount of deep frozen and ground plant tissues was combined with 750 *µ*L of ice-cold extraction solvent, consisting of methanol (LC-MS grade, Merck), chloroform (anhydrous > 99%, Sigma Aldrich), and water (MilliQ) in a ratio of 2.5:1:0.5 (v/v). Additionally, 7 *µ*L of a solution of 10 mmol of pentaerythritol (PE) and 10 mm phenyl-β-d-glucopyranoside (PGP), respectively, in water (MilliQ) was added as an internal standard mix. After ultrasonication at 4 °C for 20 min and centrifugation (4 min, 4 °C, 14,000 × *g*), the supernatant was transferred to a new 1.5 mL tube (polypropylene). Another 250 *µ*L of extraction solvent was added to the remaining pellet and after another cycle of ultrasonication and centrifugation as described before, the supernatant was combined with the previous supernatant. To induce phase separation, 350 *µ*L of water (MilliQ) was added. After thorough mixing and consecutive centrifugation (4 min, 4 °C, 14,000 × *g*), 900 *µ*L of the upper phase was transferred to a new 1.5 mL tube. Approximately 100 *µ*L of the remaining polar phase of all samples was combined. The 900 *µ*L aliquots of this mixed sample were used as quality control during measurements. The polar phases and the aliquots of the sample mix were dried in a vacuum centrifuge for 5 h at 30 °C and 0.1 mbar.

The dried extracts were derivatized as described earlier (Doerfler et al. 2013) by dissolving the metabolite pellet carefully in 20 *µ*L of 40 mg of methoxyamine hydrochloride (Sigma Aldrich) in 1 mL pyridine (anhydrous > 99,8%, Sigma Aldrich). After incubation at 30 °C and 700 rpm for 1.5 h on a thermoshaker, 80 *µ*L of *N*-methyl-*N*-trimethylsilyl-trifluoroacetamid (Macherey-Nagel) was added. The samples were incubated for 30 min at 37 °C and 750 rpm and consecutively centrifuged for 4 min at room temperature and 14,000 × *g*.

Metabolite analysis was performed on an Agilent 7890B gas chromatograph equipped with a LECO Pegasus BT-TOF mass spectrometer (LECO Corporation). Derivatized metabolites were injected through a Split/Splitless inlet equipped with an ultra-inert single tapered glass liner with deactivated glass wool (5910-2293, Agilent Technologies), a split ratio of 1:25 was used and the temperature was set to 230 °C. Components were separated with helium as carrier gas on a Restek Rxi-5Sil MS column (length: 30 m, diameter: 0.25 mm, thickness of film: 0.25 *µ*m). The initial oven temperature was set to 70 °C held for 1 min and ramped with a rate of 9 °C per minute until reaching 340 °C held for 10 min. Collection of spectra started after an acquisition delay of 280 s with a detector voltage of 1692.5 V, a rate of 15 spectra per second, and a mass range of 50 to 500 *m*/*z*. Retention indices were calculated based on the retention times of the alkane mixture C_10_-C_40_ run within each of the 2 batches. Samples were measured in randomized order and randomly distributed across the batches. Within each batch, a mixture of standard metabolites was measured for MSI level I identification of metabolites. Deconvolution, annotation, and processing of chromatograms were performed according to Zhang et al. (2023) using ChromaTOF (Version 5.55.29.0.1187, LECO Cooperation) and MS-DIAL, version 4.7 (Tsugawa et al. 2015). Areas of derivatization products of single metabolites were summed and normalized by the main targeted ion content of each sample.

#### RNA extraction and sequencing

Using the same sampled leaf material as for targeted metabolite analyses, total RNA was extracted for each sample and time point in randomized batches of 4 to 6 samples, using the QIAGEN RNeasy Mini Kit in an RNAse-free laboratory. Samples were digested using the kit's RLT buffer with 1 *µ*L/mL beta-mercaptoethanol. Elution was done in 2 steps. The purity and concentration of the extractions were measured using Nanodrop, and RIN and fragmentation profiles were obtained with a Fragment Analyzer system. RNA libraries were prepared by the Vienna Biocenter Core Facilities (VBCF) using a NEBNext stranded mRNA kit before sequencing 150-bp paired-end reads on 1 lane of Illumina NovaSeq S4.

#### RNA-seq data processing

The raw RNA-seq data were evaluated with FastQC (https://www.bioinformatics.babraham.ac.uk/projects/fastqc/) and MultiQC (Ewels et al. 2016), then quality-trimmed using AdapterRemoval v.2.3.1 (Schubert et al. 2016) with settings --trimns --trimqualities --minquality 20 --trimwindows 12 --minlength 36. The trimmed data were then aligned to both the *T. fasciculata* and *T. leiboldiana* genomes using STAR v.2.7.9 (Dobin et al. 2013) using GFF files to specify exonic regions. Because mapping bias was lowest when mapping to *T. fasciculata* (see Supplementary SI Note S8, Supplementary Figs. S17 and S18), our main analyses have been performed on the reads mapped to this genome. However, the alignments to *T. leiboldiana* were used for verification or expansion of the main analysis (Supplementary SI Note S9).

#### Differential gene expression analysis

We quantified read counts per exon using FeatureCounts from the Subread package v.2.0.3. (Liao et al. 2014) for paired-end and reversely stranded reads (-p -s 2). The counts were then summed up across exons per gene to obtain gene-level counts. The composition of the count data was investigated with PCA in EdgeR (Robinson et al. 2009). Then, counts were normalized using the TMM method in EdgeR, and every gene with a mean cpm < 1 was removed. We ran a differential gene expression (DE) analysis in maSigPro (Conesa et al. 2006), which detects genes with differential diurnal expression profiles between species using a regression approach. *T. leiboldiana* was used as the baseline in this analysis. Significant DE genes were then clustered using the hclust algorithm into modules, with the number of modules being determined with the K-means algorithm. Expression curves were plotted by taking the average expression in TPM (transcripts per million) across all replicates per species at each time. We calculated TPM by dividing the raw read count by the exonic length of the gene (RPK), which we then divided by the total sum of RPK values. Expression curves for entire clusters (Supplementary Fig. S11) were plotted by median-centering the log(TPM) of each gene and time point against the median of all genes at each time point, while expression curves for individual genes or gene families (Fig. 5C, Supplementary Figs. S10 and S14) report average TPM with standard deviation.

GO-term enrichments were performed for each cluster using the R package TopGO (Alexa and Rahnenführer 2006). Separately, known candidate genes underlying CAM and starch metabolism (see Materials and methods, Family size correction and latter section) were searched among differentially expressed genes.

#### Annotation and enrichment of circadian clock-related motifs in promoter sequences

We counted the occurrences of 4 known circadian clock-related motifs in the 2-kb upstream regions of DE genes: the Morning Element (MOE: CCACAC) (Michael et al. 2008), the Evening Element (EE: AAAATATC) (Hudson and Quail 2003), the CCA1-binding site (CBS: AAAAATCT) (Franco-Zorrilla et al. 2014), and the G-box element (G-box: CACGTG) (Michael and McClung 2002). The same was done for all other curated genes that were not DE, which we considered as background sequences. We calculated the per-kb frequency of each motif based on the counts and total promoter length (2,000 × number of genes) for both sets of genes. The percentage of change in frequency was calculated between both sets for each motif. Significance of frequency changes of circadian motifs in promotor regions of DE genes compared to non-DE genes was calculated with the Mann–Whitney *U* test. We randomly subsampled the list of non-DE genes to 5,000 observations to ensure accurate *P*-values.

For a small set of genes known to underlie key CAM enzymes, we counted the occurrence of each motif in both the 2-kb upstream region of every homolog of that gene (including non-DE paralogs) in both species, to annotate and describe circadian motifs in promoter sequences in detail. The detection of motifs was extended to 3-kb regions to allow for a more distant presence of motifs.

### Intersecting findings of gene family evolution, TE insertion, and differential gene expression

#### Spatial distribution of DE genes

The previously calculated per 1-kb window counts of robust genes were used to obtain the per-window proportion of DE genes. This was then visualized with circlize as described above. Correlations of total DE gene count per scaffold and scaffold size were calculated with Kendall's rank correlation test after testing for normality with the Shapiro–Wilk test.

#### Gene family evolution and differential gene expression

Orthogroups were split based on relative family size in *T. fasciculata* (F) versus *T. leiboldiana* (L) in the following categories: single-copy orthogroups (F = 1:L = 1), orthogroups with family size larger in *T. fasciculata* (F > L), orthogroups with family size smaller in *T. fasciculata* (F < L), and orthogroups with equal family sizes that are larger than 1 (F = L). Orthogroups unique to 1 species (F:0 or 0:L) were not considered in this analysis. We counted the number of orthogroups belonging to each category for the non-DE orthogroup set, for the subset of orthogroups containing DE genes (DE orthogroups), and for the subset of orthogroups containing DE genes that have been previously described as CAM-related (CAM-DE orthogroups). We then tested whether counts in each orthogroup category were enriched in DE orthogroups and CAM-DE orthogroups compared to non-DE orthogroups. For comparisons of non-DE orthogroups versus DE orthogroups, we used the chi-square test of independence in R. For the comparison of CAM-DE orthogroups versus non-CAM DE orthogroups, we used Fisher's exact test due to small sample sizes with 2 × 2 contingency tables of the count of orthogroups in each orthogroup category versus all other categories, in DE orthogoups versus non-DE genes. To study the effect of the reference genome used on our findings on gene family evolution in DE genes, we performed the same analysis on read counts obtained from mapping to *T. leiboldiana* (Supplementary SI Note S9).

#### Transcription factor families and gene family evolution

We identified transcription factor (TF) families by searching for genes with InterPro domains characteristic of the largest transcription factor families annotated during functional annotation with Blast2Go, which performs an InterProScan step (Paysan-Lafosse et al. 2023). The specific domains and their corresponding TF families used to identify genes are listed in Supplementary Table S12. For verification, a more conservative set of TF families was identified by finding the homologs of known *A. comosus* TFs listed on the Plant TF Database (https://planttfdb.gao-lab.org/). The majority of TFs identified using InterPro domains overlapped with homologs of known *A. comosus* TFs (81%). We then applied the same methodology as for DE orthogroups (section 5.12.2) to assess if gene families that have undergone recent evolution in gene copy number were overrepresented in the set of TF gene families. This analysis was performed on the 2 lists separately for verification and on the merger of both lists.

Using the full list of identified TF genes, we identified DE genes in both species that belonged to a TF gene family. We then obtained gene family evolution statistics as described in section 5.12.2, performing Fischer's Exact Test to compare the proportion of multicopy gene families in DE TF gene orthogroups with non-DE gene families.

#### TE insertions and differential gene expression

Intronic TE insertions were obtained using *bedtools intersect* on the GFF files of the TE and gene annotations of both species. We used the full transcript length of a gene (feature = “mRNA” in GFF file) for this analysis, and only applied “known” TE annotations and the set of curated genes. This resulted in a data set reporting the number of TE insertions per gene. We also obtained TE counts for genic regions including the 3-kb upstream region, by using *bedtools slop* with options *-l 3000 -r 0 -s*. For analyses on specific TE classes, we calculated TE insertion counts for the following 4 TE categories: LTR-Copia, LTR-Gypsy, Helitron, and DNA transposon.

We then performed 2 tests on the resulting TE counts per gene: (i) whether the proportion of genes with 1 or more TE insertions is elevated in DE genes compared to the full gene set (chi-square test), and (ii) whether the rate of TE insertions per gene measured, as the total count of intersections for each gene annotation with a TE annotation, is elevated in DE genes compared to non-DE genes (Mann–Whitney *U* test).

The same test was also applied to a restricted set of DE genes previously described as CAM-related, or involved in starch metabolism and gluconeogenesis. Then, genes of interest with a TE insertion rate higher than twice the genome-wide average were selected and the difference in number of TE insertions between orthologs of *T. leiboldiana* and *T. fasciculata* was taken in case of a one-to-one relationship.

### Accession numbers

The genome assemblies and raw data used in this study are available at NCBI-SRA under BioProject PRJNA927306. Specifically, the *T. fasciculata* genome assembly TFas_v1 can be downloaded here: https://ncbi.nlm.nih.gov/datasets/genome/GCA_029168755.2/. The *T. leiboldiana* genome assembly TLei_v1 can be found at: https://www.ncbi.nlm.nih.gov/datasets/genome/GCA_029204045.2/. The annotation of both genomes is available on the GitHub repository at: https://github.com/cgrootcrego/Tillandsia_Genomes, together with the list of orthogroups, counts table used for RNA-seq analyses, full GO-term enrichment results, and all scripts written for this manuscript. The *A. comosus* sequences used in this study stem from BioProject PRJNA371634 (F153): https://www.ncbi.nlm.nih.gov/bioproject/PRJNA371634/ and BioProject PRJNA747096 (CB5): https://www.ncbi.nlm.nih.gov/bioproject/PRJNA747096. Information on all accessions used in this study can be found in Supplementary Table S1.

## Supplementary Material

koae130_Supplementary_Data

## Supplementary data

The following materials are available in the online version of this article.

**Supplementary Figure S1.** Loadings of 45 individual metabolites on PC1 and PC2.

**Supplementary Figure S2.** Genome size measurement histograms.

**Supplementary Figure S3.** Heterozygosity and genome size estimation with a k-mer based approach implemented in findGSE for *Tillandsia fasciculata* and *T. leiboldiana*.

**Supplementary Figure S4.** Scaffolds sizes and per-scaffold count of orthologous genes in the de novo assemblies of *T. fasciculata* and *T. leiboldiana*.

**Supplementary Figure S5.** Mitotic metaphase chromosomes and karyotypes of *Tillandsia fasciculata* and *Tillandsia leiboldiana*.

**Supplementary Figure S6.** TE, GC, and gene contents at 3 examples of syntenic chromosome triplets.

**Supplementary Figure S7.** In-depth visualization of large-scale rearrangements between *T. fasciculata* and *T. leiboldiana* based on local alignments with less than a 90% overlap with any other alignment.

**Supplementary Figure S8.** Genome-wide distribution of *d*_N_/*d*_S_ values between single-copy orthologous genes.

**Supplementary Figure S9.** Heatmap of z-score normalized expression values of CAM-related DE genes featured in Fig. 6.

**Supplementary Figure S10.** Average expression curve of PEPC kinase (PPCK) in *T. fasciculata* and *T. leiboldiana* with standard deviation.

**Supplementary Figure S11.** Per-gene expression curves of all differentially expressed genes, spread over 7 co-expression clusters inferred with MaSigPro.

**Supplementary Figure S12.** Relationship between differentially expressed (DE) gene count per scaffold and scaffold size.

**Supplementary Figure S13.** Distribution of DE genes across the genome.

**Supplementary Figure S14.** Average expression curve of Aquaporin 2 to 6 in *T. fasciculata* and *T. leiboldiana*.

**Supplementary Figure S15.** Distribution of heterozygous sites per 1,000 mappable variants on a logarithmic scale.

**Supplementary Figure S16.** Distribution of mean per-gene coverage across different gene family categories.

**Supplementary Figure S17**. Percentage of uniquely mapping RNA-seq reads for 24 samples to the genome assemblies of *T. fasciculata*, *T. leiboldiana*, and *A. comosus*.

**Supplementary Figure S18.** Proportion of RNA-seq reads mapping in multiple genomic locations for 24 samples mapped to the genome assemblies of *T. fasciculata*, *T. leiboldiana*, and *A. comosus*.

**Supplementary Table S1.** List of accessions used in this study.

**Supplementary Table S2.** Assembly statistics.

**Supplementary Table S3.** TE abundances.

**Supplementary Table S4.** Orthology statistics.

**Supplementary Table S5.** List of CAM-related expanded orthogroups.

**Supplementary Table S6.** Full list of candidate genes under positive selection.

**Supplementary Table S7.** Full list of co-expression modules.

**Supplementary Table S8.** Per-kb frequencies of 4 promoter motifs associated with circadian clock transcription factors in DE and non-DE genes.

**Supplementary Table S9.** Annotation of circadian promoter motifs in the upstream regions of CAM genes.

**Supplementary Table S10.** Overview of gene family evolution in transcription factor families.

**Supplementary Table S11.** List of DE genes related to CAM, starch metabolism, and gluconeogenesis with a high TE insertion rate.

**Supplementary Table S12.** List of InterPro codes used to identify transcription factor families in the *T. leiboldiana* and *T. fasciculata* gene annotations.

**Supplementary Notes.**

**Supplementary Data Set 1.** Metabolomic Compound Abundances reported as raw area read-outs from MS-DIAL, including the level of identification according to the Metabolomics Standards Initiative.

**Supplementary Data Set 2.** Description and detailed results of statistical tests.

## Dive Curated Terms

The following phenotypic, genotypic, and functional terms are of significance to the work described in this paper:

LHY Gramene: Aco016649.1
LHY Araport: Aco016649.1

TMM Gramene: Method in EdgeR to normalize read counts (trimmed mean of M values)
TMM Araport: Method in EdgeR to normalize read counts (trimmed mean of M values)

PPCK1 Gramene: Aco013938.1
PPCK1 Araport: Aco013938.1

BUSCO Gramene: Benchmarking Universal Single-Copy Orthologs
BUSCO Araport: Benchmarking Universal Single-Copy Orthologs

AUGUSTUS Gramene: AUGUSTUS is a program that predicts genes in eukaryotic genomic sequences
AUGUSTUS Araport: AUGUSTUS is a program that predicts genes in eukaryotic genomic sequences

SUT2 Gramene: Aco004135.1
SUT2 Araport: Aco004135.1

metabolite CHEBI: CHEBI:25212
