# Supplementary material for: CAM evolution is associated with gene family expansion in an explosive bromeliad radiation
Source: Plant Cell. 2024 Apr 30;36(10):4109–31. doi: 10.1093/plcell/koae130 (PMC11449062; doi:10.1093/plcell/koae130)
Supplement: koae130_Supplementary_Data [file koae130_supplementary_data.zip › Supplementary_material.pdf]

Supplementary Information

"CAM evolution is associated with gene family expansion in an explosive bromeliad radiation"

C. Groot Crego, J. Hess, G. Yardeni, M.de La Harpe, C. Priemer, F. Beclin, S. Saadain, L. A. Cauz-Santos, E. M. Temsch, H. Weiss-Schneeweiss, M.H.J. Barfuss, W. Till, W. Weckwerth, K. Heyduk, C. Lexer, O. Paun, T. Leroy

1. SUPPLEMENTARY FIGURES 2
2. SUPPLEMENTARY TABLES 20
3. SUPPLEMENTARY NOTES 32
NOTE 1: GENOME SIZE AND KARYOTYPE OF T. FASCICULATA AND T. LEIBOLDIANA 32
NOTE 2: PRE-ASSEMBLY ESTIMATION OF PER-ACCESSION HETEROZYGOSITY 32
NOTE 3: IDENTIFYING MAIN SCAFFOLDS IN DE NOVO ASSEMBLY 33
NOTE 4: ON THE SPATIAL DISTRIBUTION OF GC AND TE CONTENT IN BROMELIAD GENOMES 34
NOTE 5: IDENTIFYING LARGE-SCALE REARRANGEMENTS BETWEEN T. FASCICULATA AND T. LEIBOLDIANA 35
NOTE 6: SELECTING RAPIDLY EVOLVING GENE FAMILIES 37
NOTE 7: DETAILED DESCRIPTION OF CANDIDATE GENES FOR POSITIVE SELECTION 37
NOTE 8: CHOICE OF REFERENCE GENOME, MAPPING BIAS AND MAPPING STATISTICS FOR RNA-SEQ ANALYSIS 39
NOTE 9: DIFFERENTIAL GENE EXPRESSION USING THE T. LEIBOLDIANA ASSEMBLY 40
NOTE 10: ON THE DIFFERENTIAL EXPRESSION AND GENE FAMILY EXPANSION OF CAM-RELATED GENES 41
NOTE 11: ON THE SUCCESS OF DE NOVO ASSEMBLY OF HIGHLY REPETITIVE GENOMES 45
4. REFERENCES 47

1. Supplementary Figures

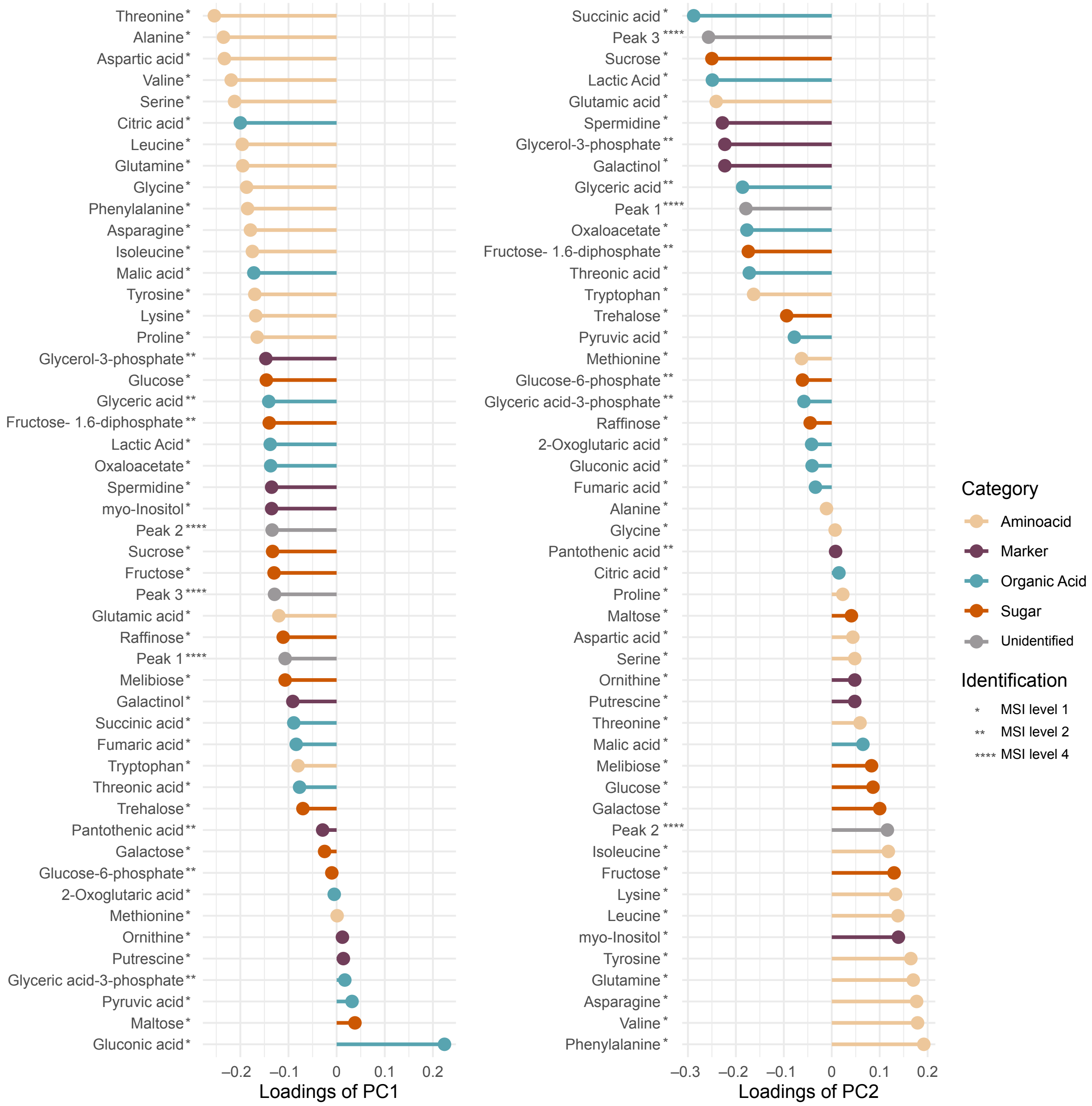

Supplementary Figure S1: Loadings of 45 individual metabolites on PC1 and PC2 (Supports Figure 2). Besides amino acids, the organic acids malate, citrate and gluconic acid contribute most to differentiation along PC1. Sugars appear as important contributors to differentiation along PC2. MSI Level 1: identified compound (mass spectra and retention index compared to reference compounds analyzed under same conditions). MSI level 2: Putatively annotated compound (EI mass spectra and retention index compared to in-house library). MSI level 4: Unidentified compound.

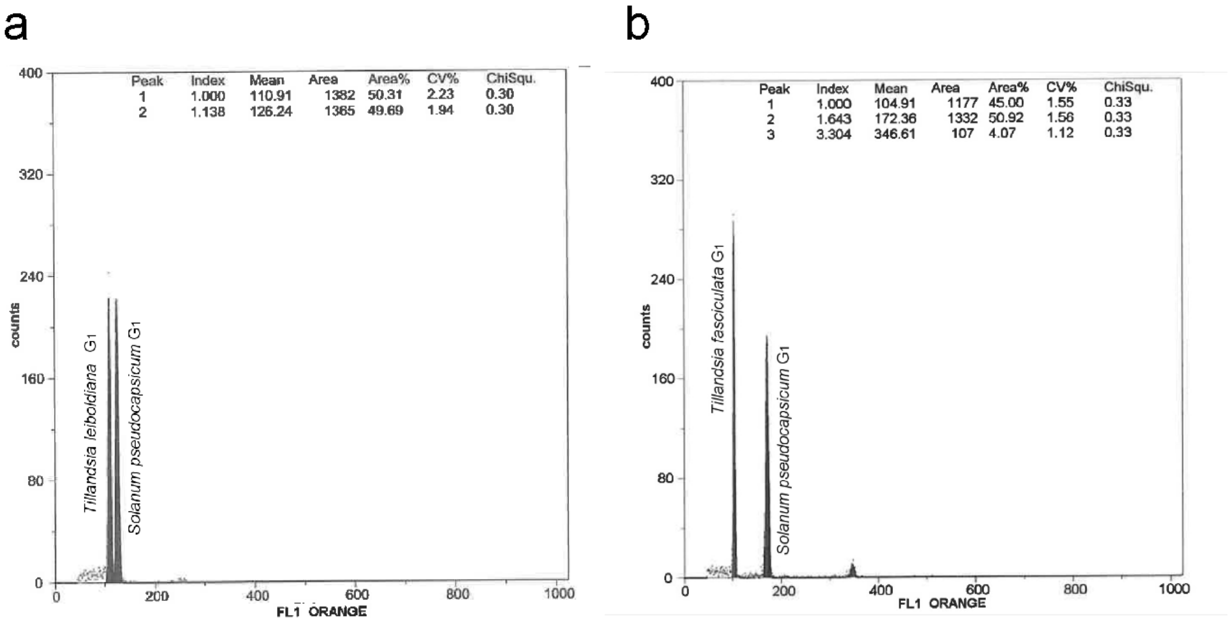

Supplementary Figure S2: Genome size measurement histograms (Supports Figure 3). A) Histogram of one exemplary run of *Tillandsia fasciculata* and **B)** of *T. leiboldiana* showing the mean G_1 nuclei peak positions on the x-axis (fluorescence intensity) of the samples and the standard organism (*Solanum pseudocapsicum*, 1.295pg/1C).

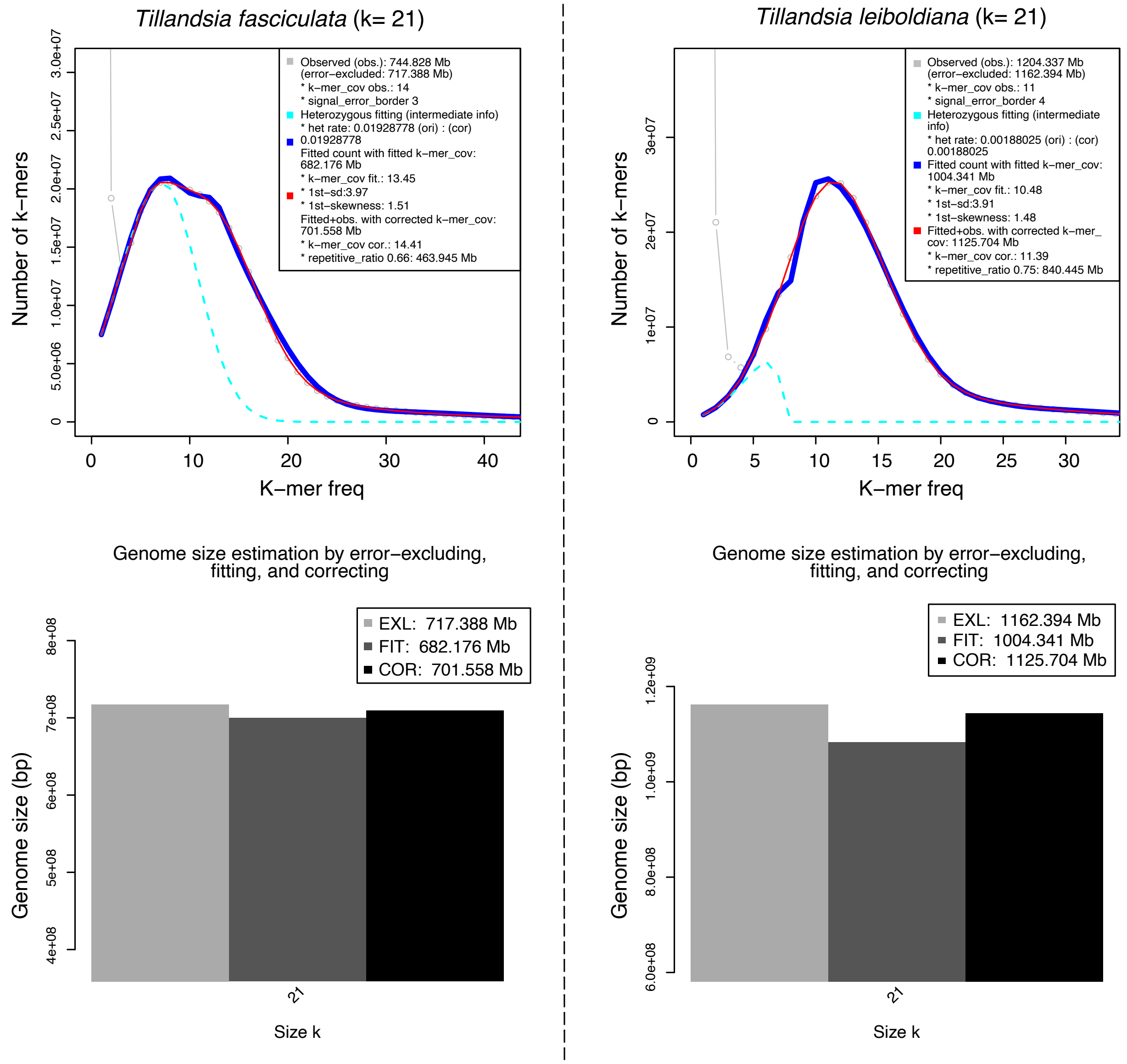

Supplementary Figure S3: Heterozygosity and genome size estimation with a k-mer based approach implemented in findGSE for *Tillandsia fasciculata* and *T. leiboldiana* (Supports Figure 3).

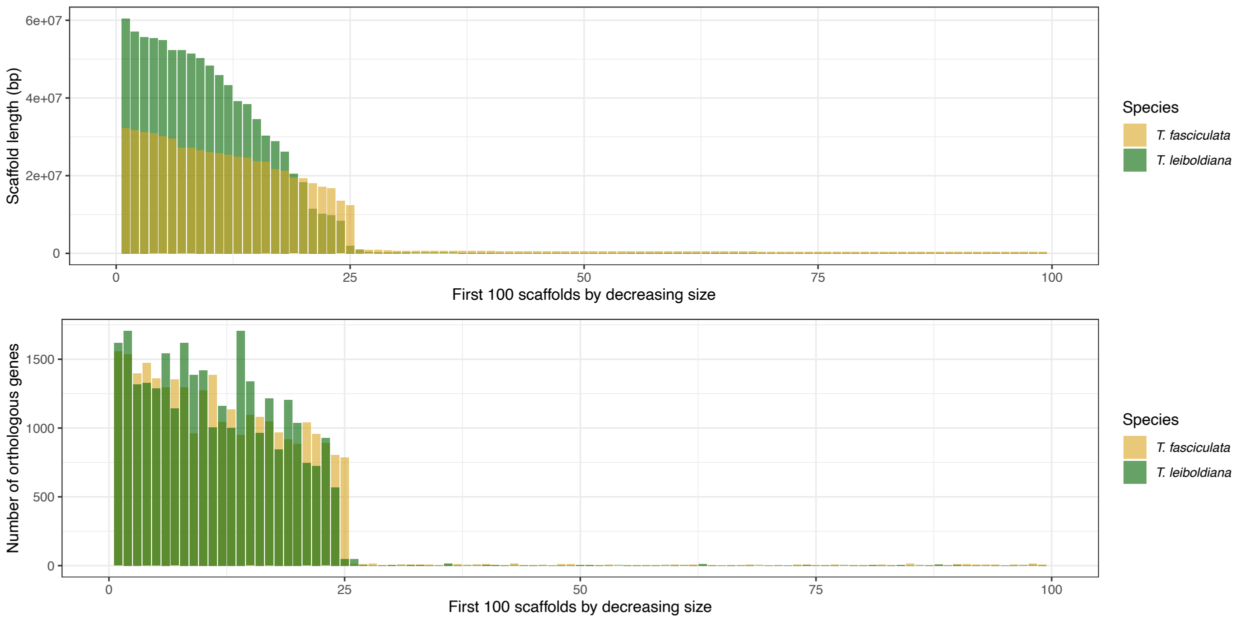

Supplementary Figure S4: Scaffolds sizes and per-scaffold count of orthologous genes in the de novo assemblies of *T. fasciculata* and *T. leiboldiana* (Supports Figure 3). Histogram of scaffold sizes (top) and count of orthologous genes per scaffold (bottom) for the top 100 largest scaffolds of both assemblies.

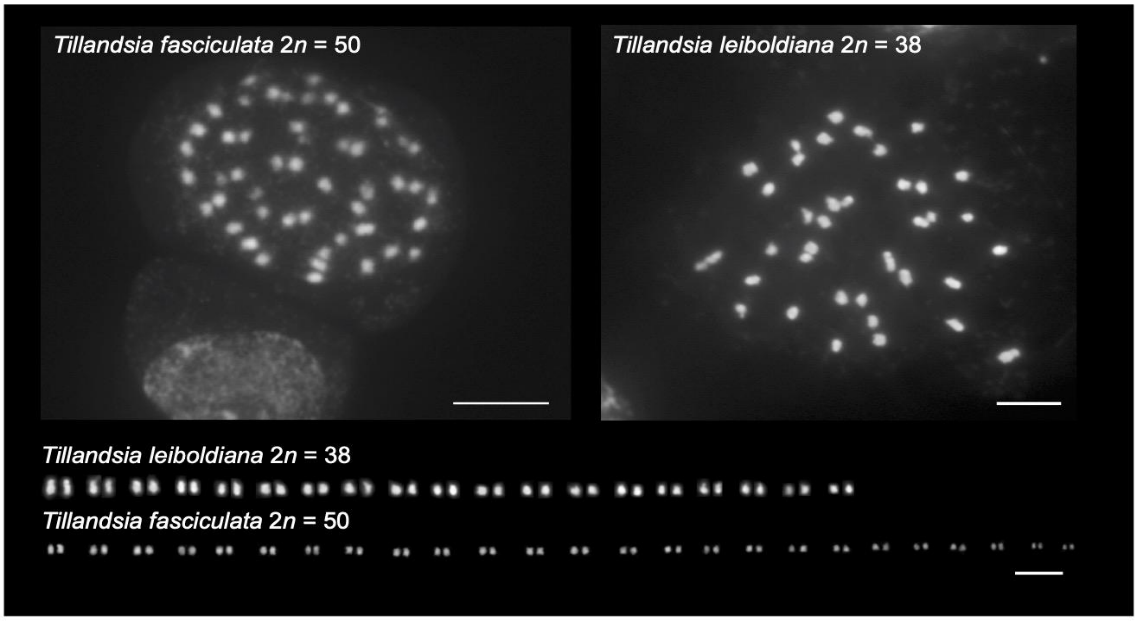

Supplementary Figure S5: Mitotic metaphase chromosomes and karyotypes of *Tillandsia fasciculata* and *Tillandsia leiboldiana* (Supports Figure 3). Scale bar, 5µm.

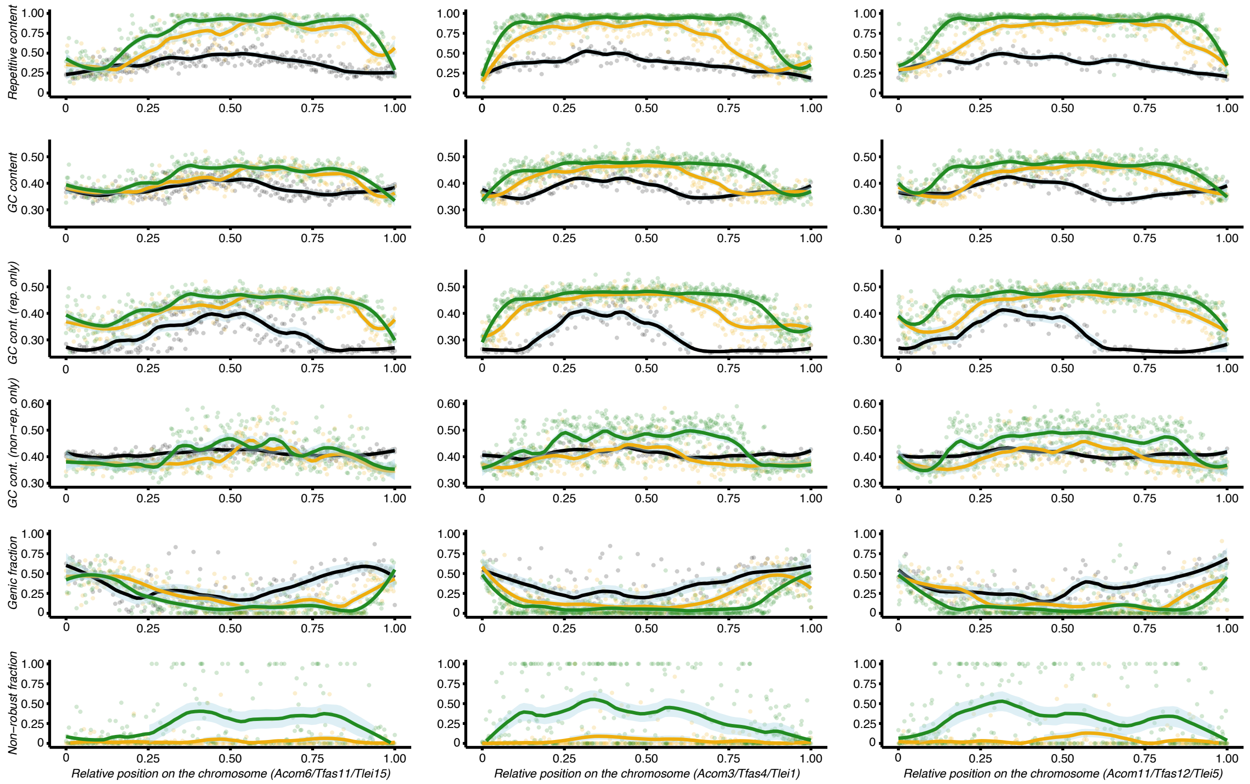

Supplementary Figure S6: TE, GC, and gene content at three examples of syntenic chromosome triplets (Supports Figure 3). Each triplet contains a scaffold of *A. comosus* (black), *T. fasciculata* (yellow) and *T. leiboldiana* (green). Each column represents a separate chromosome triplet. Each dot corresponds to an estimate in a non-overlapping 100 kb window. The line corresponds to the local regression (loess). Row-wise, from top to bottom, the plots show: (1) per-window proportion of soft-masked position in the assemblies (repetitive content), (2) GC content at all non-N positions (soft-masked or not), (3) GC content at soft-masked positions only, (4) GC content at positions that were not soft-masked,, (5) per-window proportion of bases falling in genes (genic fraction) and (6) the proportion of the genic fraction corresponding to non-robust genes (i.e. 1 minus this fraction corresponds to “robust” gene regions). This latter information is only provided for our two reference assemblies.

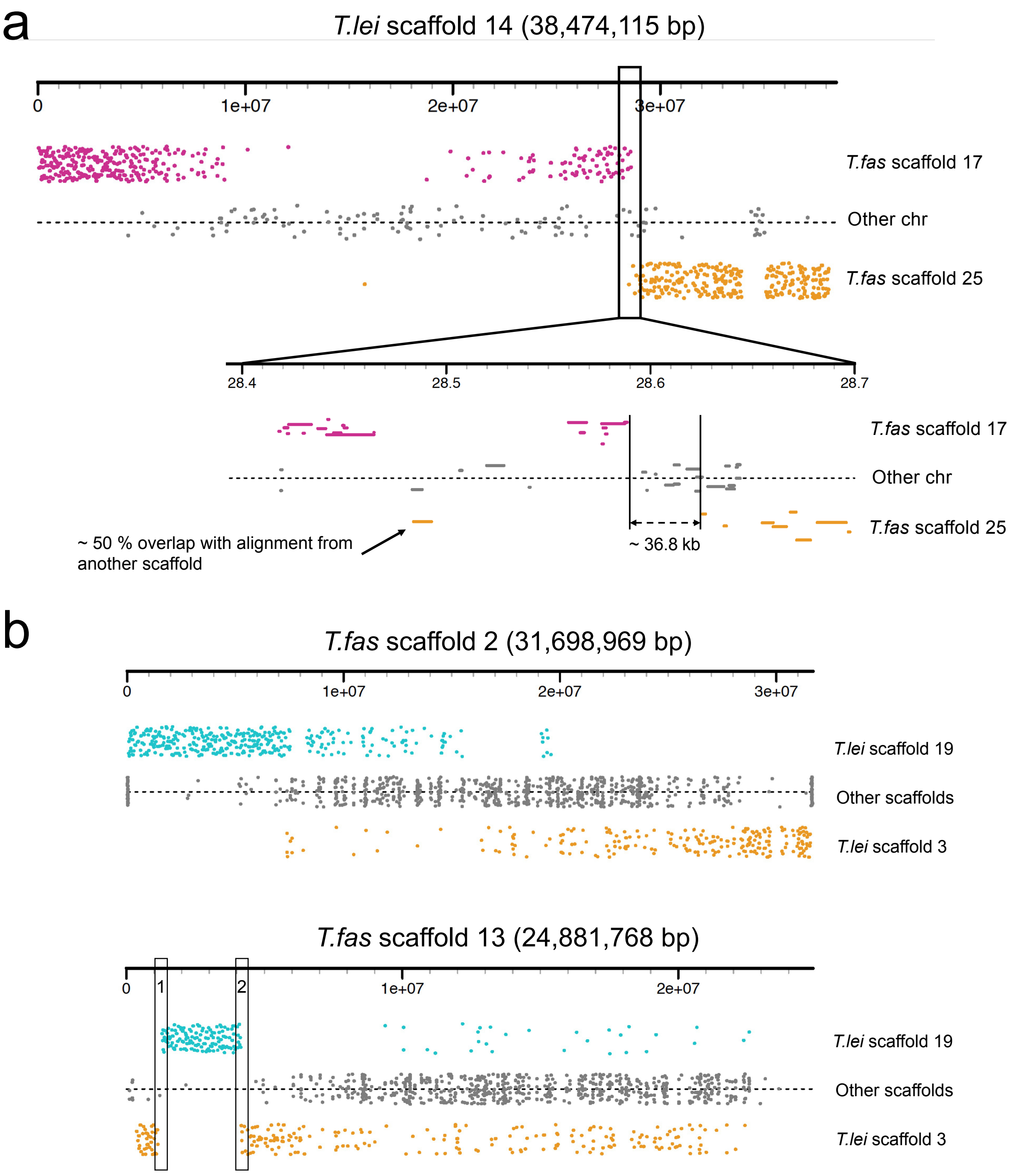
a
T.lei scaffold 14 (38,474,115 bp)
0
1e+07
2e+07
3e+07
T.fas scaffold 17
Other chr
T.fas scaffold 25
28.4
28.5
28.6
28.7
T.fas scaffold 17
Other chr
T.fas scaffold 25
~ 50 % overlap with alignment from another scaffold
~ 36.8 kb
b
T.fas scaffold 2 (31,698,969 bp)
0
1e+07
2e+07
3e+07
T.lei scaffold 19
Other scaffolds
T.lei scaffold 3
T.fas scaffold 13 (24,881,768 bp)
0
1
2
1e+07
2e+07
T.lei scaffold 19
Other scaffolds
T.lei scaffold 3

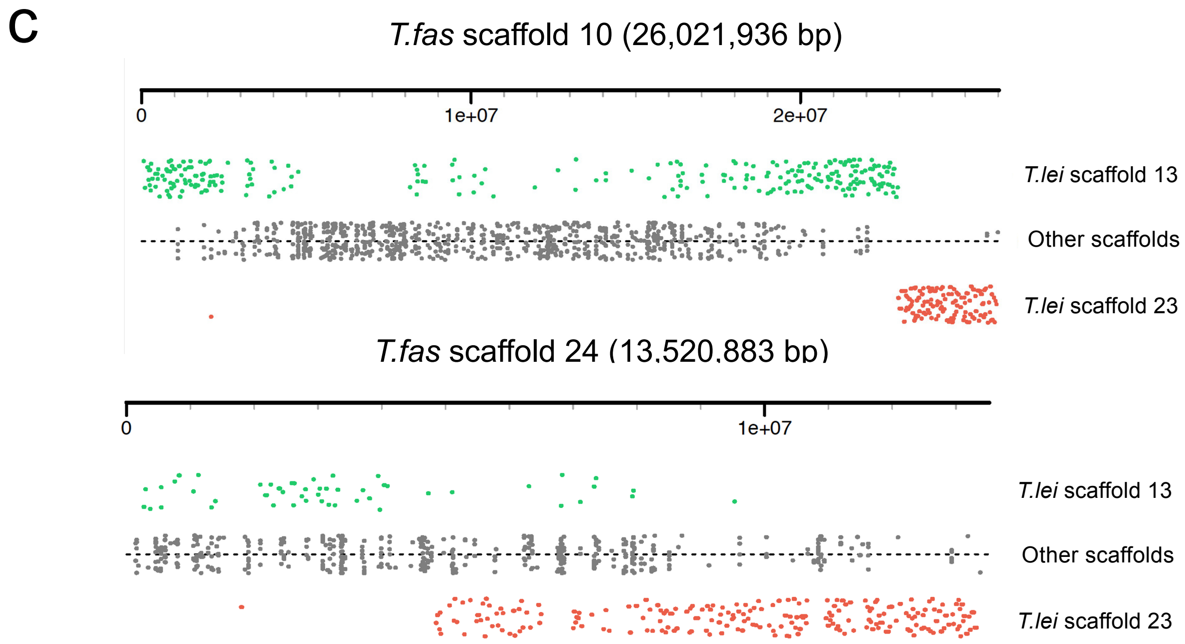

Supplementary Figure S7: In-depth visualisation of large-scale rearrangements between *T. fasciculata* and *T. leiboldiana* based on local alignments with less than a 90 % overlap with any other alignment (Supports Figure 3). A) Potential fusion of scaffold 14 in *T. leiboldiana*, with enlargement of the breakpoint area. **B)** Translocation 1 – alignments were too sparse to determine a breakpoint on scaffold 2. Breakpoint 1 on scaffold 13 in *T. fasciculata* was not supported by raw PacBio alignments, however breakpoint 2 was. **C)** Translocation 2 – we find PacBio alignment support for the breakpoint on scaffold 10, but alignments were too sparse on scaffold 24 to determine a breakpoint. For more in-depth analysis and visualization of PacBio alignments, see the document 'Tfas_Tlei_rearrangements.pdf' on our github repository. Species names are abbreviated as T. lei for *T. leiboldiana* and T. fas for *T. fasciculata.*

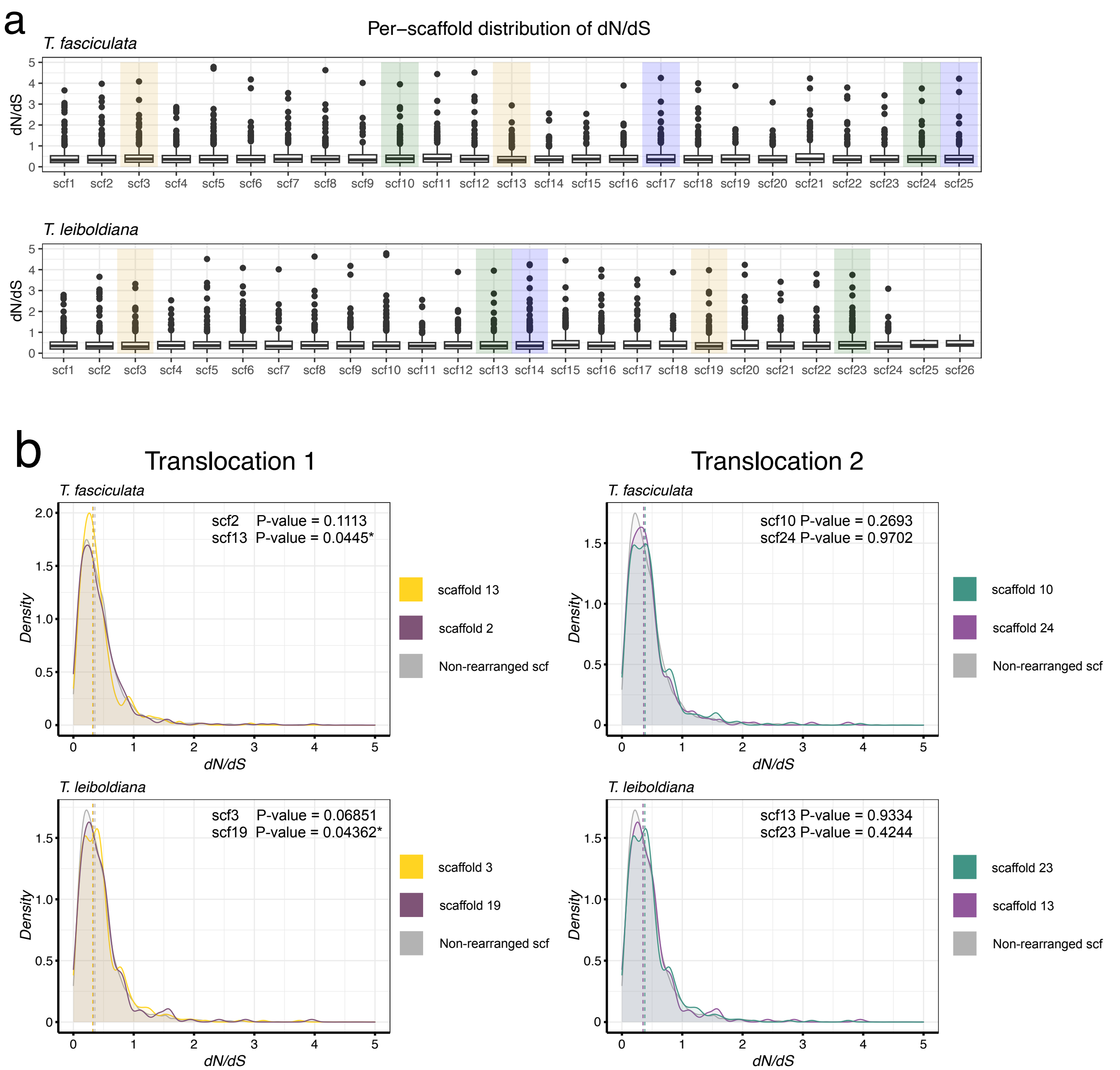

Supplementary Figure S8: Genome-wide distribution of d_N/d_S values between single-copy orthologous genes (Supports Figure 4). A) Boxplot of d_N/d_S values in each scaffold (scf) of both assemblies. For ease of reading, the y-axis is cut-off at a d_N/d_S value of five. Therefore, candidate genes with high values are not shown here. Scaffolds highlighted in colours are involved in the three reported large-scale rearrangements: (1) chromosomal fusion in *T. leiboldiana* (blue), (2) translocation 1 (yellow), and (3) translocation 2 (green). Dots represent outliers, whiskers reach to the minimum and maximum value. Lower box border shows the 25th percentile, the upper box border the 75th percentile. The thick horizontal line inside the box represents the median. **B)**

Distribution of d_N/d_S values of all non-rearranged chromosomes versus chromosomes involved in translocations. P-values were obtained through the Mann Whitney U test, comparing dN/dS values of the respective scaffold against the background of non-rearranged scaffolds.

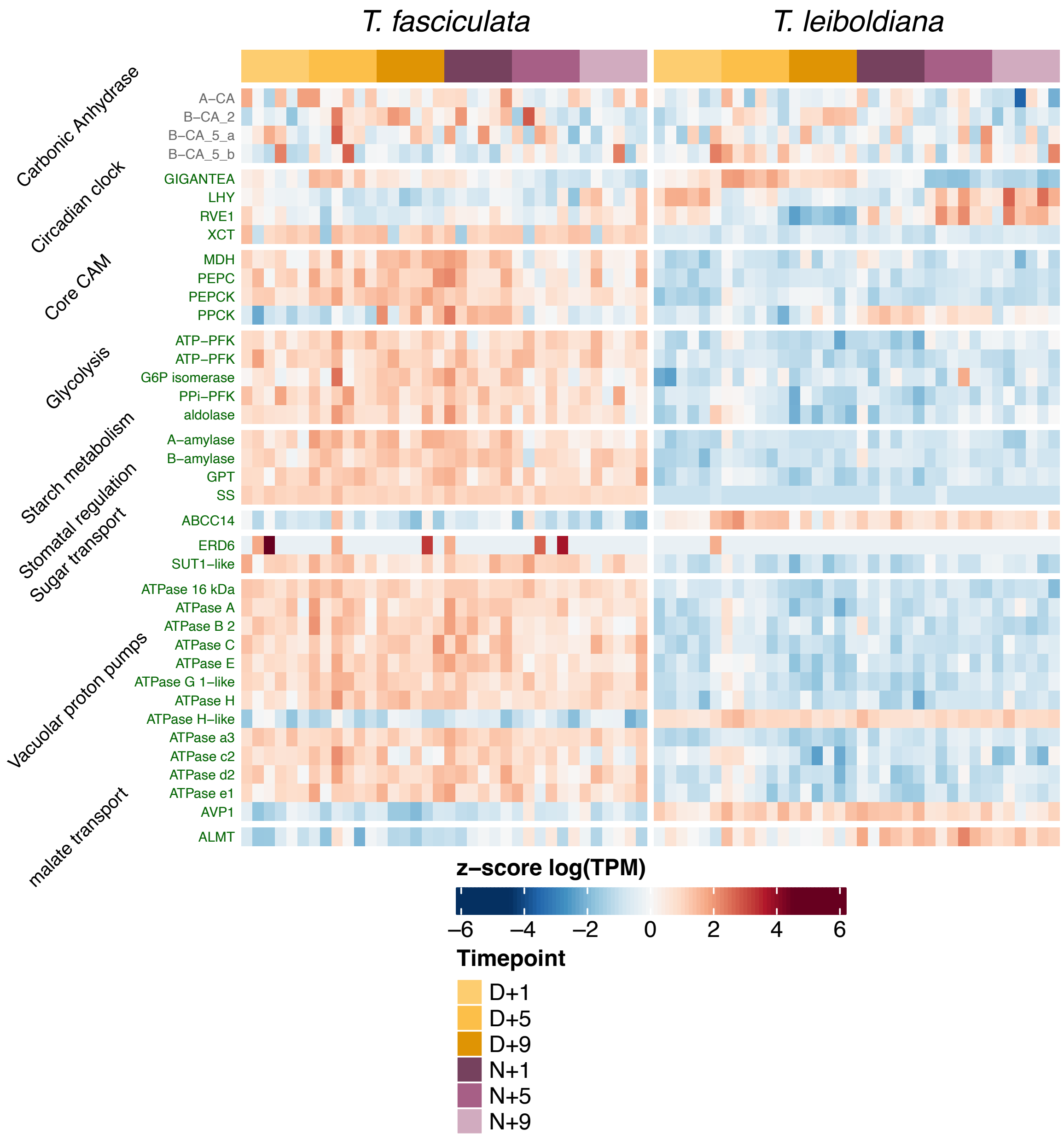

Supplementary Figure S9: Heatmap of z-score normalised expression values of CAM-related DE genes featured in Figure 6 (Supports Figure 6). Normalizing was performed on log(TPM) values (Transcripts Per Million). Genes in grey were not considered differentially expressed. Time is indicated in hours after the lights go off

(N=Night) and after they go on (D=Day), e.g. D+1 is a sampling time point one hour after lights turned on. Abbreviations: A-CA - alpha carbonic anhydrase; B-CA - beta carbonic anhydrase; LHY - Late Elongated Hypocotyl; RVE1 – REVEILLE 1; XCT - XAP5 CIRCADIAN TIMEKEEPER; MDH - malate dehydrogenase; PEPC - phosphoenolpyruvate carboxylase; PEPCK – PEP carboxykinase; PPCK - PEPC kinase; ATP-PFK – ATP-dependent Phosphofructokinase; PPi-PFK; PPi-dependent Phosphofructokinase; G6P – glucose-6-phosphate; GPT – glucose-6-phosphate/phosphate translocator; SS – starch synthase; ABCC14 – ABC transporter C14; ERD6 – sugar transporter ERD6; SUT1 – SUT1 sugar transporter; AVP1 - PPI-energised vacuolar membrane proton pump; ALMT - Aluminium dependent malate transporter;. SI Note 10 contains a detailed explanation and interpretation of the expression curves of these genes.

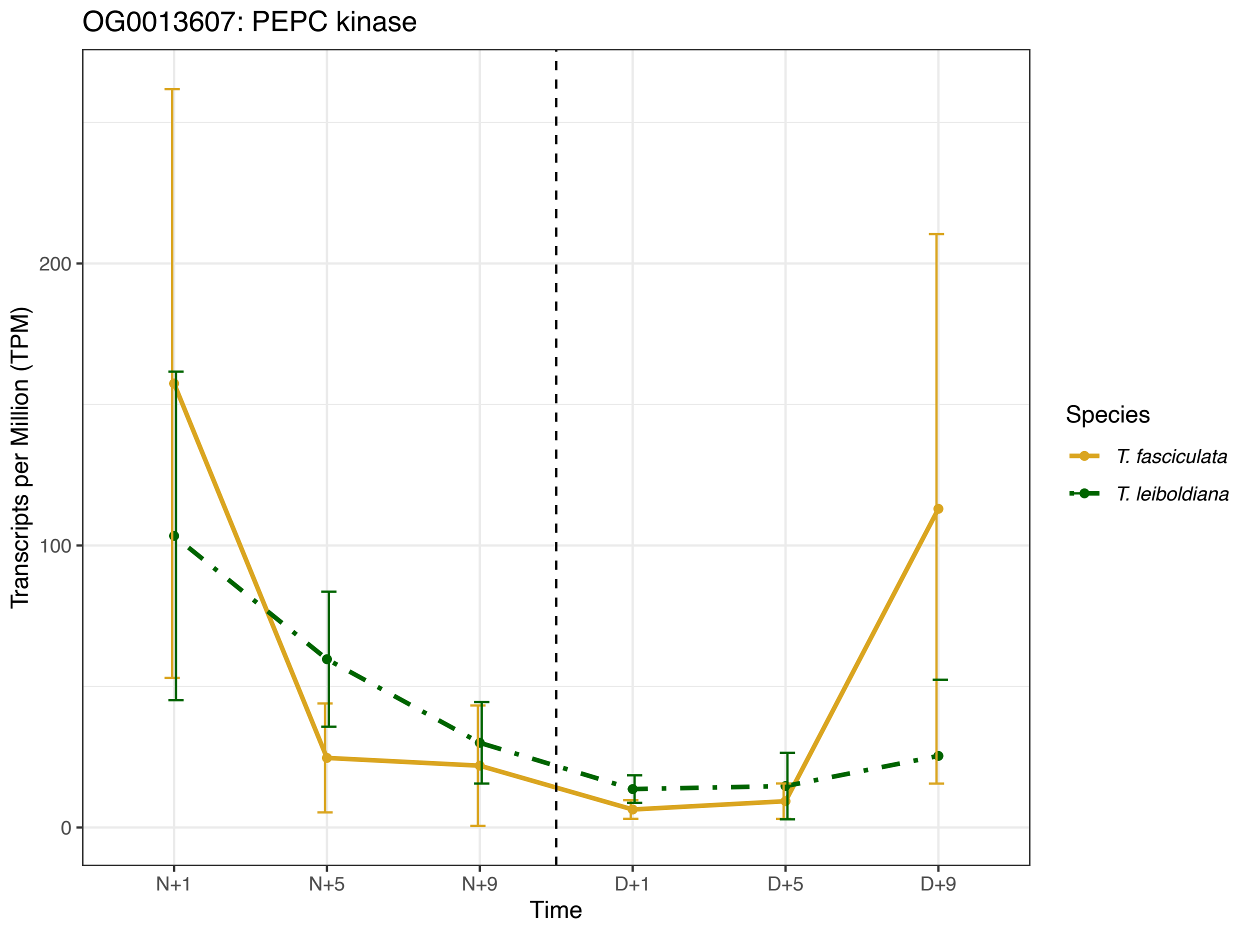

Supplementary Figure S10: Average expression curve of PEPC kinase (PPCK) in *T. fasciculata* and *T. leiboldiana* with standard deviation (Supports Figures 5 and 6). The dashed vertical line marks the point where the light was switched on. Time is indicated in hours after the lights go off (N=Night) and after they go on (D=Day).

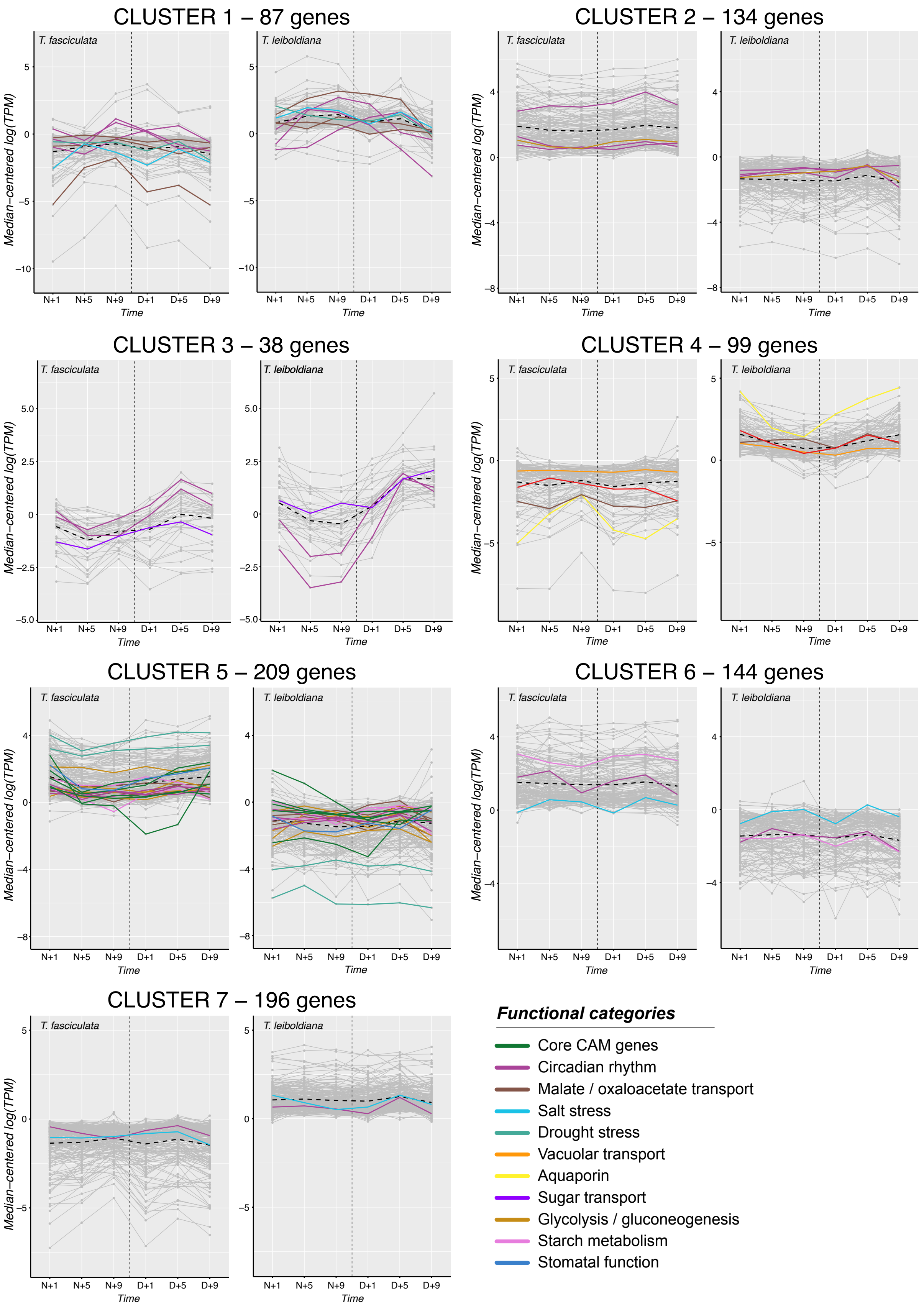
CLUSTER 1 – 87 genes
CLUSTER 2 – 134 genes
CLUSTER 3 – 38 genes
CLUSTER 4 – 99 genes
CLUSTER 5 – 209 genes
CLUSTER 6 – 144 genes
CLUSTER 7 – 196 genes
T. fasciculata
T. leiboldiana
Median−centered log(TPM)
Time
N+1
N+5
N+9
D+1
D+5
D+9
Functional categories
Core CAM genes
Circadian rhythm
Malate / oxaloacetate transport
Salt stress
Drought stress
Vacuolar transport
Aquaporin
Sugar transport
Glycolysis / gluconeogenesis
Starch metabolism
Stomatal function

Supplementary Figure S11: Per-gene expression curves of all differentially expressed genes, spread over 7 co-expression clusters inferred with MaSigPro (Supports Figures 5 and 6). *T. fasciculata* was used as reference genome. The dashed vertical line marks the point where the light was switched on. Time is indicated in hours after the lights go off (N=Night) and after they go on (D=Day). Highlighted expression curves represent candidate genes underlying CAM-related functions. The colours correspond to specific subfunctions, laid out in the legend below.

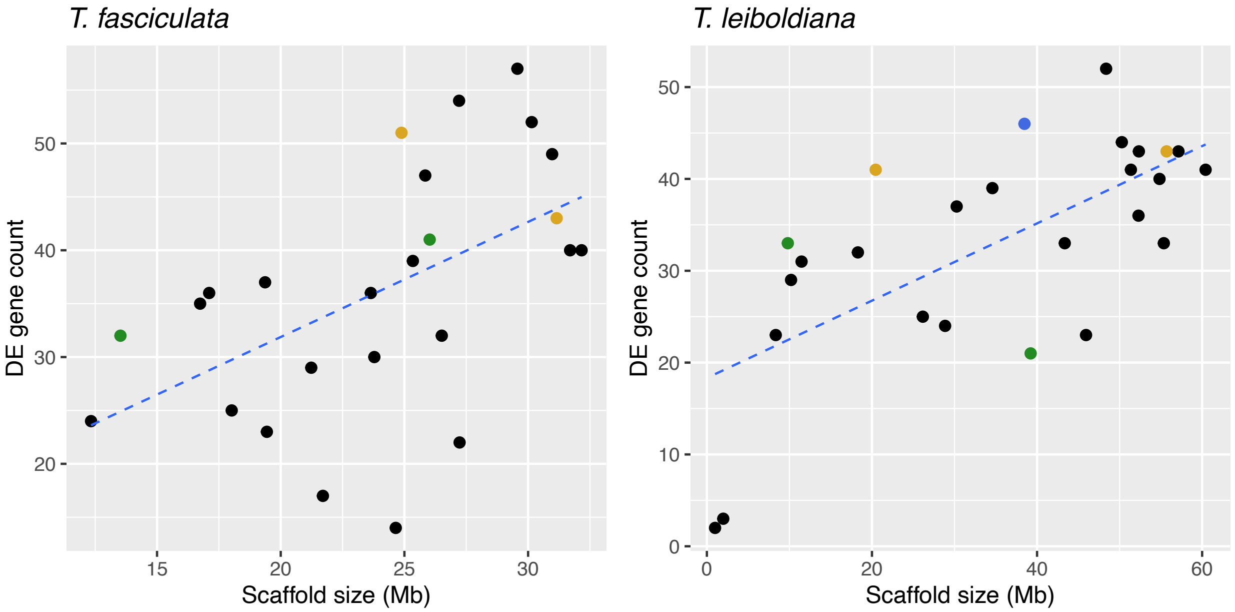

Supplementary Figure S12: Relationship between differentially expressed (DE) gene count per scaffold and scaffold size (Supports Results section 2.9.1). Scaffolds involved in large-scale rearrangements are highlighted in colours; translocation 1 (yellow), translocation 2 (green), chromosomal fusion (blue). Dashed line computed with geom_smooth using a linear model.

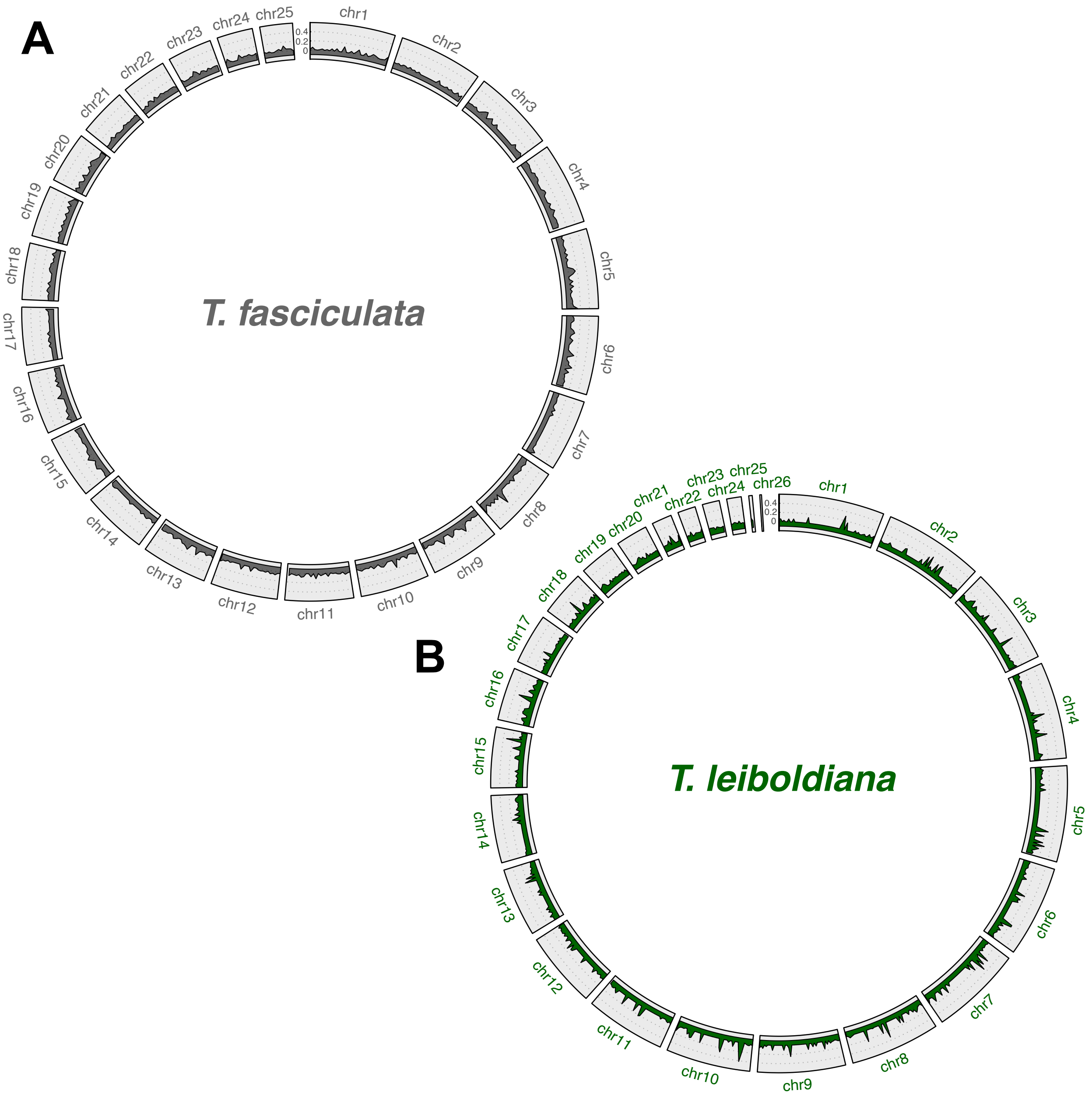

Supplementary Figure S13: Distribution of DE genes across the genome (Supports Results section 2.9.1). Proportion of genes per 1 Mb window that are differentially expressed across the *T. fasciculata* **(A)** and *T. leiboldiana* **(B)** assembly. The dotted lines mark the 0, 0.2 and 0.4 density mark.

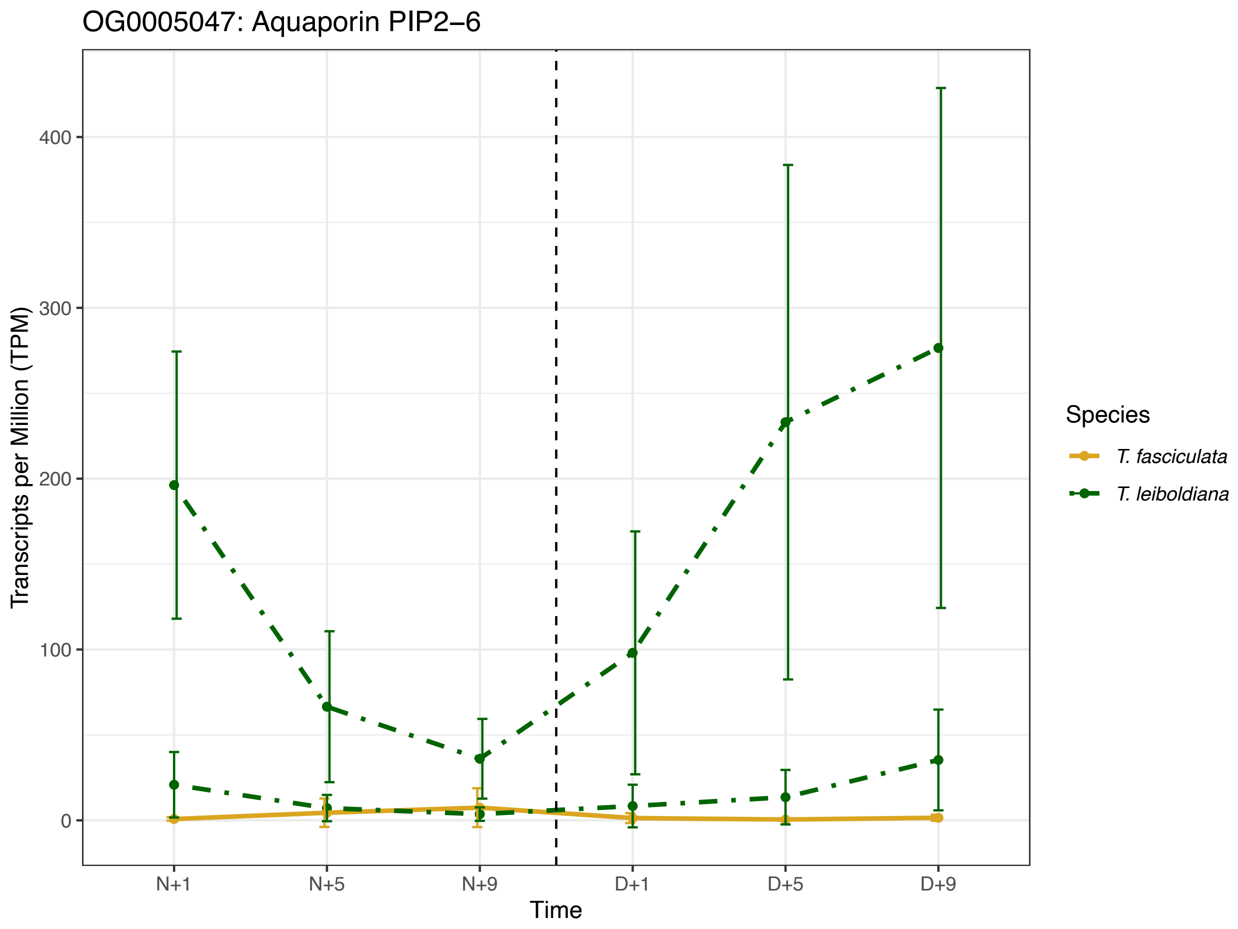

Supplementary Figure S14: Average expression curve of Aquaporin PIP2-6 in *T. fasciculata* and *T. leiboldiana* with standard deviation (Supports Figures 5 and 6). The dashed vertical line marks the point where the light was switched on. Time is indicated in hours after the lights go off (N=Night) and after they go on (D=Day).

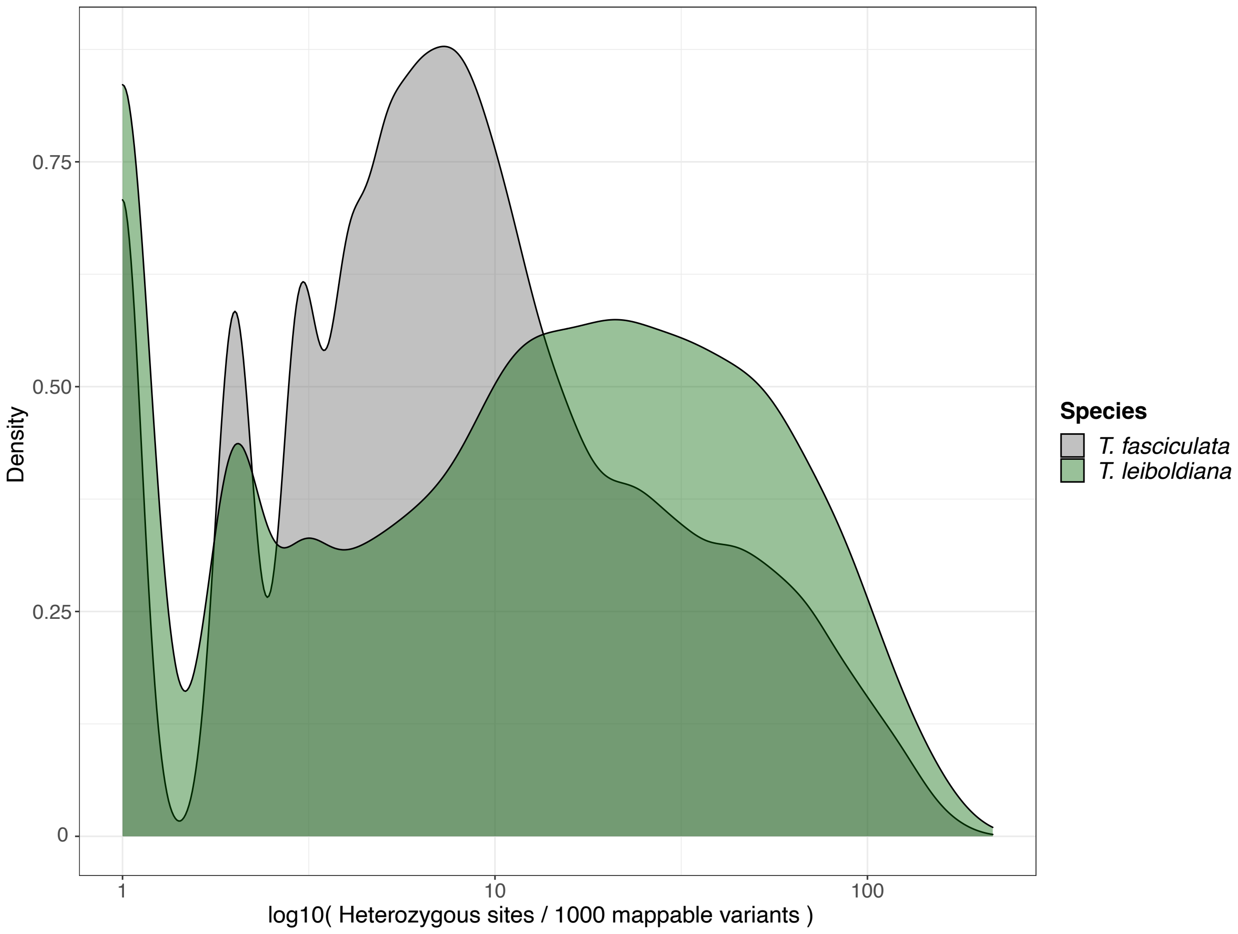

Suppementary Figure S15: Distribution of heterozygous sites per 1000 mappable variants on a logarithmic scale (Supports Figure 3).

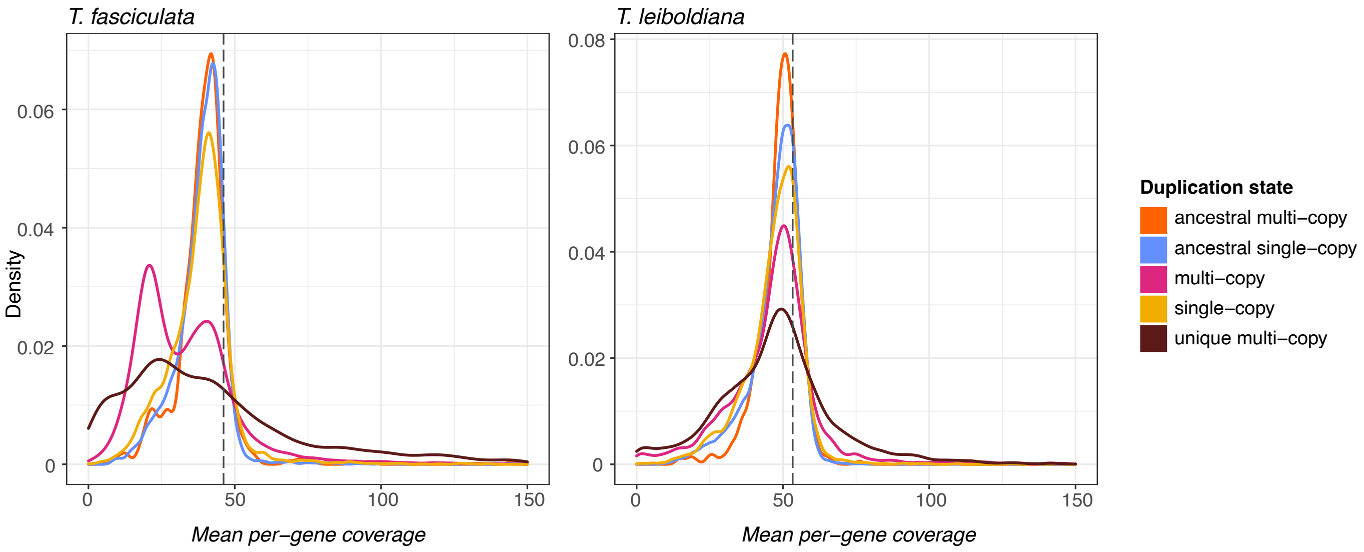

Supplementary Figure S16: Distribution of mean per-gene coverage across different gene family categories (Supports Figure 5). For further explanation of the different categories, see Material & Methods. Grey dashed lines indicate the whole-genome mean coverage.

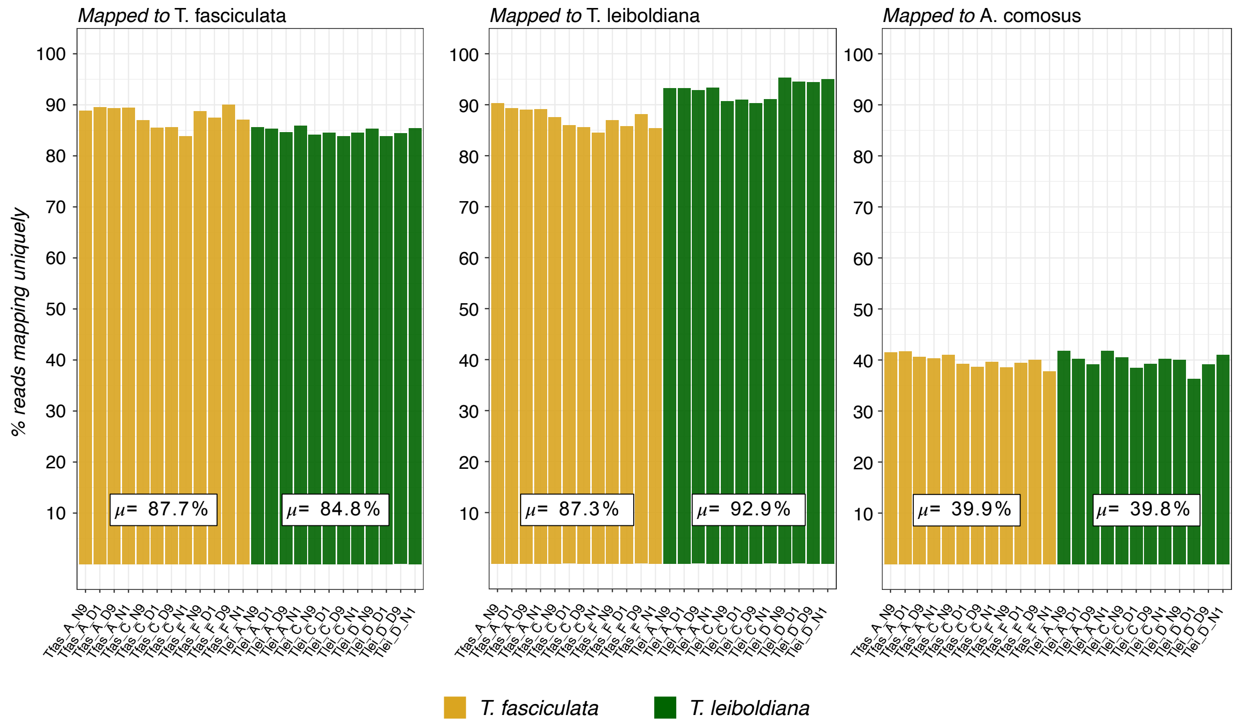

Supplementary Figure S17: Percentage of uniquely mapping RNA-seq reads for 24 samples to the genome assemblies of *T. fasciculata*, *T. leiboldiana* and *A. comosus* (Supports Figure 5). The mean unique mapping rate per species and genome is reported inside the white squares.

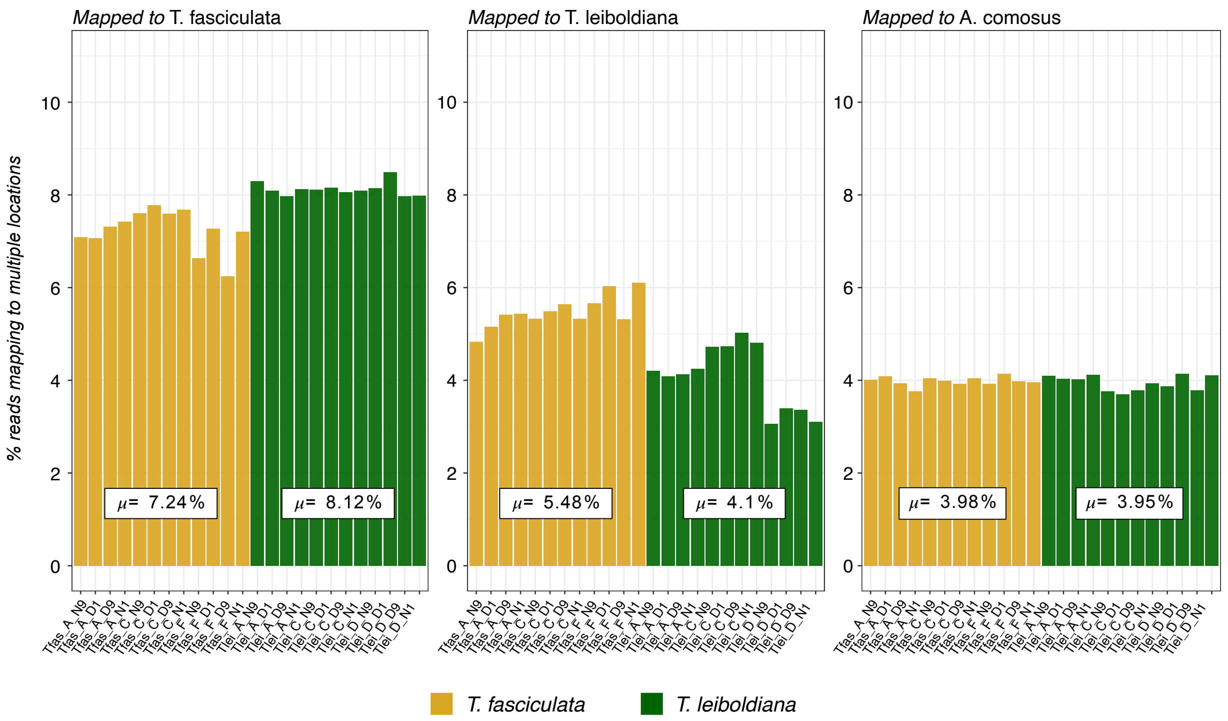

Supplementary Figure S18: Proportion of RNA-seq reads mapping in multiple genomic locations for 24 samples mapped to the genome assemblies of *T. fasciculata*, *T. leiboldiana* and *A. comosus* (Supports Figure 5). The mean mapping rate for each species and genome are highlighted in white squares.

2. Supplementary tables

Supplementary Table S1: List of accessions used in this study. Abbreviations used in this table: WU = Institutional Code (Wien Universität), HBV = Hortus Botanicus Vindobonensis (Botanical Garden of the University of Vienna), DBG = Deutsche Bromelien-Gesellschaft (German Bromeliad Society), s.n. = sin nomero (without number), s.d. = sin datos (without data), s.l. = sensu lato

Source	Accession code	Herbarium accession	DNA №	Collector	Species	Country	Locality	Comment
Genome Assembly								
Botanical Garden of the Univiersity of Vienna	HBV 0024657 (B179/91)	WU 0013642	MHJB-B1840	W. & S. Till 7116	Tillandsia fasciculata s.l.	Costa Rica	Prov. Puntarenas, SW declivities of Cordillera de Tilaran, along the road from Sta. Elena to Rancho Grande.	
Botanical Garden of the University of Vienna	HBV 0024715 (B82/91)	WU 0003058	MHJB-B1842	W. & S. Till 7112	Tillandsia leiboldiana s.l.	Costa Rica	Prov. Alajuela, 2 km N San Ranion	
RNA-SEQ for Gene Annotation								
Botanical Garden of the University of Vienna	HBV 0026950 (B103/94)	WU 0006321	MHJB-B1839	G. Noller 9106	Tillandsia fasciculata	Guatemala	Depto. Sololá, Lago de Atitlán	Originally sequenced for De La Harpe et. al. (2020)
Botanical Garden of the Univiersity of Vienna	HBV 0024656 (B108/94)	WU 0002124	MHJB-B360	K.-D. & R. Ehlers EM890701	Tillandsia fasciculata	Mexico	Estdo. Chiapas, Sumidero Cañon bei Tuxtla Gutierrez	Originally sequenced for De La Harpe et. al. (2020)
Botanical Garden of the Univiersity of Vienna	HBV 0025322 (B100/91)	WU 0013632	MHJB-B2295	W. & S. Till 7005	Tillandsia fasciculata	Costa Rica	Prov. Heredia, Barva north of Heredia	Originally sequenced for De La Harpe et. al. (2020)
Botanical Garden of the University of Vienna	HBV 0000663 (B84/91, BRO000613)	WU 0001725, WU 0003008	MHJB-B323	W. & S. Till 7043	Tillandsia leiboldiana	Costa Rica	Prov. Cartago, Turrialba, Centro Agronomico Tropical de Investigacion y Enseñanza (CATIE)	
RNA-SEQ for Time Course Experiment								
Botanical Garden of the University of Vienna	HBV 0025322 (B100/91)	s.d.	MHJB-B2295	W. & S. Till 7005	Tillandsia fasciculata	Costa Rica	Prov. Heredia, Barva north of Heredia	Tfas_E in RNA-seq analysis
Botanical Garden of the Univiersity of Vienna	HBV 0025194 (B293/96)	s.d.	MHJB-B2296	S. Schatzl 77/59	Tillandsia fasciculata	Mexico	Estdo. Jalisco, ca. 20 km S of Porto Vall	Tfas_B in RNA-seq analysis
Botanical Garden of the Univiersity of Vienna	HBV 0025334 (B99B53-1)	WU 0008562, WU 0013708	MHJB-B1838	E. Kamm s.n.	Tillandsia fasciculata	Honduras	s.d.	Tfas_C in RNA-seq analysis
Botanical Garden of the Univiersity of Vienna	HBV 0024655 (B99/91) (90/91)	WU 0013632, WU 0013754	MHJB-B1841	W. & S. Till 7004	Tillandsia fasciculata	Costa Rica	Prov. Heredia, Barva north of Heredia	Tfas_D in RNA-seq analysis
Botanical Garden of the Univiersity of Vienna	HBV 0025326 (B90/91)	s.d.	MHJB-B2297	W. & S. Till 7006 (7004)	Tillandsia fasciculata	Costa Rica	Prov. Heredia, Barva north of Heredia	Tfas_A in RNA-seq analysis
Botanical Garden of the Univiersity of Vienna	HBV 0024657 (B179/91)	WU 0013642	MHJB-B1840	W. & S. Till 7116	Tillandsia fasciculata s.l.	Costa Rica	Prov. Puntarenas, SW declivities of Cordillera de Tilaran, along the road from Sta. Elena to Rancho Grande.	Tfas_F in RNA-seq analysis
Botanical Garden of the University of Vienna (Corn.Bak BV)	HBV 0032437 (Bak 126)	s.d.	MHJB-B1960	s.d. (DBG Sept. 2011)	Tillandsia leiboldiana	Costa Rica	above Carteso	Tlei_A in RNA-seq analysis
Botanical Garden of the University of Vienna (Corn.Bak BV)	HBV 0032433 (Bak 27)	s.d.	MHJB-B1957	P. Bak s.n.	Tillandsia leiboldiana	Costa Rica	s.d.	Tlei_C in RNA-seq analysis
Botanical Garden of the Univiersity of Vienna	HBV 0024715 (B82/91)	WU 0003058	MHJB-B1842	W. & S. Till 7112	Tillandsia leiboldiana s.l.	Costa Rica	Prov. Alajuela, 2 km N San Ranion	Tlei_D in RNA-seq analysis
Botanical Garden of the University of Vienna (Corn.Bak BV)	HBV 0032436 (Bak 119)	s.d.	MHJB-B1959	s.d. (DBG Sept. 2011)	Tillandsia leiboldiana	Mexico	Estdo. Puebla, Amixtlán	Tlei_E in RNA-seq analysis
Botanical Garden of the University of Vienna (Corn.Bak BV)	HBV 0032434 (Bak 37)	s.d.	MHJB-B1958	P. Bak s.n.	Tillandsia leiboldiana	Costa Rica	s.d.	Tlei_F in RNA-seq analysis
Botanical Garden of the University of Vienna (Corn.Bak BV)	HBV 0032435 (Bak 45)	s.d.	MHJB-B1956	P. Bak s.n.	Tillandsia leiboldiana	Honduras	s.d.	Tlei_G in RNA-seq analysis

Supplementary Table S2: Summary statistics of the *T. fasciculata* and *T. leiboldiana* assembly and gene annotation

Assembly statistics	*T. fasciculata*	*T. leiboldiana*
Total length (bp)	837,577,910	1,198,225,148
Total scaffold count (> 1 kb)	2,321	10,433
Total contig count	8,625	20,447
N50 (Mb)	23,642	43,365
N90 (Kb)	145,438	27,985
L50	16	12
L90	565	2,898
GC content	42.81 %	44.73 %
Uniquely mapping RNA-seq reads	69.13 %	92.37 %
Complete BUSCO genes	91.8 %	88.1 %
Duplicated BUSCO genes	6.2 %	1.9 %
Fragmented BUSCO genes	5.2 %	5.4 %
**Gene model statistics**	*T. fasciculata*	*T. leiboldiana*
Gene model count	34,886	38,180
Average length (bp)	4,090	4,225
Complete BUSCO genes	89.7 %	85.3 %
Duplicated BUSCO genes	11.6 %	6.5 %
Fragmented BUSCO genes	5.2 %	7.9 %
Uniquely mapping RNA-seq reads	64.61 %	84.76 %
Gene models with AED-score > 0.5	93 %	89.9 %
Scaffolds containing gene models	1,191	2,621
**Statistics of functional annotation**	*T. fasciculata*	*T. leiboldiana*
Gene models with BLAST	31,883	33,971
Gene models with GO terms	26,505	27,148
Gene models with Blast2Go annotation	24,319	24,633

Supplementary Table S3: Abundances of LTR, TIR and Helitron classes in main contigs of *T. fasciculata* and *T. leiboldiana*

		Tillandsia fasciculata			*Tillandsia leiboldiana*		
Class	Type	Element count	Total length (bp)	Proportion of genome	Element count	Total length (bp)	Proportion of genome
	Total	692,254	392,992,866	65.52 %	1,268,380	697,969,745	77.07 %
LTR							
	Copia	85,077	72,241,681	12.04 %	226,304	117,239,901	12.95 %
	Gypsy	136,750	130,003,938	21.67 %	243,366	215,847,337	23.84 %
	Unknown	179,371	110,637,301	18.45 %	357,701	222,072,889	24.52 %
	Total	401,198	312,882,920	52.16 %	827,371	555,160,127	61.31 %
TIR							
	CACTA	26,027	6,822,973	1.14 %	22,788	6,785,096	0.75 %
	Mutator	78,405	20,912,187	3.49 %	227,557	87,286,075	9.64 %
	PIF_Harbinger	13,059	3,335,951	0.56 %	14,124	3,580,678	0.4 %
	Tc1_Mariner	3,435	648,896	0.11 %	1,589	372,253	0.04 %
	hAT	31,871	8,365,061	1.39 %	21,951	6,587,992	0.73 %
	Total	152,797	40,085,068	6.69 %	288,009	104,612,094	11.56 %
Helitron							
	Helitron	118,069	35,165,625	5.86 %	130,790	33,461,880	3.7 %

Supplementary Table S4: Orthology statistics before and after curation (removal of plastid genes and size correction). Size correction was not applied to *A. comosus,* however, unique genes to this species were removed from the dataset.

	Before curation			After curation		
General and single-copy statistics	*A. comosus*	*T. fasciculata*	*T. leiboldiana*	*A. comosus*	*T. fasciculata*	*T. leiboldiana*
Number of genes assigned to orthogroups	21,045	26,325	23,584	20,416	24,397	22,968
Proportion of input sequences assigned	78 %	87.5 %	75 %	-	-	-
Number of single-copy genes in a given species	12,794	14,311	15,537	12,602	15,236	15,479
Proportion	52.5 %	54.36 %	65.88 %	61.73 %	62.45 %	67.39 %
Number of single-copy orthologues (1:1:1)	10,012	10,012	10,012	10,707	10,707	10,707
Proportion	47.57 %	38.03 %	42.45 %	52.44 %	43.89 %	46.62 %
Number of single-copy orthologues in *Tillandsia*	-	13,128	13,128	-	14,086	14,086
Proportion	-	49.87 %	55.66 %	-	57.74 %	61.33 %
Multi-copy statistics						
Number of multi-copy genes in a given species	8,260	12,014	8,011	7,809	9,161	7,489
Proportion	39.25 %	45.64 %	33.97	38.25 %	37.55 %	32.6 %
Number of genes in orthogroups with family size *T. fas* > *T. lei*	2,594	6,976	2,709	1,178	4,170	1,356
Proportion	12.33 %	26.50 %	11.49 %	5.77 %	17.09 %	5.9 %
Number of genes in orthogroups with family size T. fas < T. lei	817	905	2,222	725	833	2,079
Proportion	3.88 %	3.44 %	9.42 %	35.51 %	34.14 %	9.05 %
Number of genes in orthogroups with family size T. fas = T. lei	1,244	1,265	1,265	1,291	1,258	1,258
Proportion	5.91 %	4.81 %	5,36 %	6.32 %	4.6 %	4.6 %
Unique gene statistics						
Number of unique genes	-	3,101	3,192	-	3,051	3,154
Proportion	-	14.74 %	13.53 %	-	12.51 %	13.73 %

Supplementary Table S5: List of CAM-related expanded orthogroups in either *T. fasciculata* or *T. leiboldiana*. Orthogroups marked by an asterisk (*) contain genes recovered in DGE analyses.

Orthogroup	Conformation of gene family size	Function	Description
Expanded in *T. fasciculata*			
OG0000267*	6 : 5 :4	Pyrophosphate-energized vacuolar membrane	Proton pump located on the tonoplast that energizes the night-time accumulation of malate in
OG0000469	0 : 15 : 1	Cytosolic enolase 3	Catalyses the conversion of 2-phosphoglycerate (2-PG) to phosphoenolpyruvate (PEP) in the
OG0000539*, OG0004427	2 : 8 : 3, 1 : 2 :1	Vacuolar-type proton ATPase subunit H	Subunit of a V-ATPase proton pump, which energizes the night-time accumulation of malate
OG0000555	0 : 7 : 1	Enolase	Catalyses the conversion of 2-phosphoglycerate (2-PG) to phosphoenolpyruvate (PEP) in the
OG0000601*	3 : 3 : 2	V-type proton ATPase 16 kDa proteolipid	Subunit of a V-ATPase proton pump, which energizes the night-time accumulation of malate
OG0001440	2 : 2 : 1	Succinate dehydrogenase [ubiquinone]	Subunit of the Succinate-ubiquinone oxidoreductase complex (complex II), which is
OG0001710	1 : 2 : 1	Acidic beta-fructofuranosidase 1	Vacuolar acid invertase involved in the accumulation of hexoses in the vacuole during the
OG0002059	2 : 2 : 1	Malate dehydrogenase (MDH)	Converts oxaloacetate to malate as part of the carbon fixation module of CAM.
OG0002712, OG0003619	2 : 2 : 1, 1 : 2 : 1	Late embryogenesis abundant (LEA) protein	Involved in drought stress response [4].
OG0003207	1 : 3 : 1	Beta carbonic anhydrase 5	Involved in carbon fixation by catalyzing the conversion of CO2 to bicarbonate, which is the
OG0003437*	1 : 2 : 1	succinate dehydrogenase subunit 6,	Plant-specific [6] subunit of the succinate dehydrogenase complex involved in anchoring the
OG0005044*	1 : 2 : 1	Protein XAP5 CIRCADIAN TIMEKEEPER	Involved in the regulation of light response [8], the circadian clock [9], and disease resistance
OG0005172*	1 : 2 : 1	mitochondrial NAD-dependent isocitrate	Catalyzes the third step of the tricarboxylic acid cycle and is a putative alternative carbon
OG0000580*	4 : 3 : 2	Pyrophosphate--fructose 6-phosphate 1-	Catalyzes the second step of the glycolysis in the cytoplasm, and can generally mostly access
OG0001599*	3 : 2 : 1	granule-bound starch synthase	Involved in starch synthesis by catalyzing the production of amylose and the structure of
Expanded in *T. leiboldiana*			
OG0000288	2 : 1 : 13	Histidine kinase 3	Regulator of stomatal opening in *Arabidopsis thaliana* in response to light and stress [13]
OG0000320	4 : 4 : 5	glyceraldehyde-3-phosphate dehydrogenase 2,	Catalyzes the sixth step of the glycolysis.
OG0000323	6 : 2 : 4	Aquaporin NIP1-1	Membrane transporter of water and small solutes, which shows diel expression in pineapple
OG0000507	7 : 1 : 2	NADP-dependent isocitrate dehydrogenase	Catalyzes the third step of the tricarboxylic acid cycle.
OG0001563	3 : 1 : 2	Enolase	Catalyses the conversion of 2-phosphoglycerate (2-PG) to phosphoenolpyruvate (PEP) in the
OG0001933	2 : 1 : 2	Phosphoglycerate kinase	Catalyses the first energy-producing step of the glycolysis, and also the reversed step in
OG0004307	1 : 1 : 2	Late embryogenesis abundant (LEA) protein	Involved in drought stress response [4].
OG0005047*	1 : 1 : 2	Aquaporin PIP2-6	Membrane transporter of water and small solutes, which shows diel expression in pineapple

Citations

[1]	Wai, C.M., VanBuren, R., Zhang, J., Huang, L., Miao, W., Edger, P.P., Yim, W.C., Priest, H.D., Meyers, B.C., Mockler, T., Smith, J.A.C., Cushman, J.C. and Ming, R. (2017), Temporal and spatial transcriptomic and microRNA dynamics of CAM photosynthesis in pineapple. Plant J, 92: 19-30. https://doi.org/10.1111/tpj.13630
[2]	Araújo WL, Nunes-Nesi A, Osorio S, et al. Antisense inhibition of the iron-sulphur subunit of succinate dehydrogenase enhances photosynthesis and growth in tomato via an organic acid-mediated effect on stomatal aperture. Plant Cell. 2011;23(2):600-627. doi:10.1105/tpc.110.081224
[3]	J. T. Christopher , JAM. Holtum, Patterns of Carbon Partitioning in Leaves of Crassulacean Acid Metabolism Species during Deacidification, Plant Physiology, Volume 112, Issue 1, September 1996, Pages 393–399, https://doi.org/10.1104/pp.112.1.393
[4]	Xiao B, Huang Y, Tang N, Xiong L. Over-expression of a LEA gene in rice improves drought resistance under the field conditions. Theor Appl Genet. 2007;115(1):35-46. doi:10.1007/s00122-007-0538-9
[5]	Ming, R., VanBuren, R., Wai, C. et al. The pineapple genome and the evolution of CAM photosynthesis. Nat Genet 47, 1435–1442 (2015). https://doi.org/10.1038/ng.3435
[6]	Millar AH, Eubel H, Jänsch L, Kruft V, Heazlewood JL, Braun HP. Mitochondrial cytochrome c oxidase and succinate dehydrogenase complexes contain plant specific subunits. Plant Mol Biol. 2004;56(1):77-90. doi:10.1007/s11103-004-2316-2
[7]	Christine Schikowsky, Jennifer Senkler, Hans-Peter Braun, SDH6 and SDH7 Contribute to Anchoring Succinate Dehydrogenase to the Inner Mitochondrial Membrane in Arabidopsis thaliana , Plant Physiology, Volume 173, Issue 2, February 2017, Pages 1094–1108, https://doi.org/10.1104/pp.16.01675
[8]	Ellen L. Martin-Tryon, Stacey L. Harmer, XAP5 CIRCADIAN TIMEKEEPER Coordinates Light Signals for Proper Timing of Photomorphogenesis and the Circadian Clock in Arabidopsis , The Plant Cell, Volume 20, Issue 5, May 2008, Pages 1244–1259, https://doi.org/10.1105/tpc.107.056655
[9]	Liu, Lei, Xiaoyun Li, Li Yuan, Guofang Zhang, Hui Gao, Xiaodong Xu, and Hongtao Zhao. "XAP5 CIRCADIAN TIMEKEEPER specifically modulates 3'splice site recognition and is important for circadian clock regulation partly by alternative splicing of LHY and TIC." Plant Physiology and Biochemistry 172 (2022): 151-157.
[10]	Xu, Yong-Ju, Yang Lei, Ran Li, Ling-Li Zhang, Zhi-Xue Zhao, Jing-Hao Zhao, Jing Fan et al. "XAP5 CIRCADIAN TIMEKEEPER Positively Regulates RESISTANCE TO POWDERY MILDEW8. 1–Mediated Immunity in Arabidopsis." Frontiers in Plant Science 8 (2017): 2044.
[11]	Nadine Töpfer and others, Alternative Crassulacean Acid Metabolism Modes Provide Environment-Specific Water-Saving Benefits in a Leaf Metabolic Model, The Plant Cell, Volume 32, Issue 12, December 2020, Pages 3689–3705, https://doi.org/10.1105/tpc.20.00132
[12]	Nancy Wieland Carnal , Clanton C. Black, Soluble Sugars as the Carbohydrate Reserve for CAM in Pineapple Leaves : Implications for the Role of Pyrophosphate:6-Phosphofructokinase in Glycolysis, Plant Physiology, Volume 90, Issue 1, May 1989, Pages 91–100, https://doi.org/10.1104/pp.90.1.91
[13]	Marchadier E, Hetherington AM. Involvement of two-component signalling systems in the regulation of stomatal aperture by light in Arabidopsis thaliana. New Phytol. 2014;203(2):462-468. doi:10.1111/nph.12813
[14]	Zhu, F., Ming, R. Global identification and expression analysis of pineapple aquaporins revealed their roles in CAM photosynthesis, boron uptake and fruit domestication. Euphytica 215, 132 (2019). https://doi.org/10.1007/s10681-019-2451-0

Supplementary Table S6: Full list of candidate genes for adaptive sequence evolution.

Orthogroup	Genes	dN/dS	dN	dS	adj p-value	Function
One-to-one orthologues						
OG0002972	Tfasc_v1.01130-RA, Tlei_v1.02341-RA	Inf	0,0416	0,0004	0,00162	jacalin-related lectin 3-like
OG0005000	Tfasc_v1.24851-RA, Tlei_v1.26186-RA	Inf	0,0247	0,0002	0,00628	cucumber peeling cupredoxin-like
OG0006253	Tfasc_v1.06027-RA, Tlei_v1.01410-RA	Inf	0,0221	0,0002	0,00066	mitochondrial prohibitin-3
OG0009278	Tfasc_v1.29917-RA, Tlei_v1.20972-RA	Inf	0,0214	0,0002	0,00155	chloroplastic Peroxiredoxin-2E-2
OG0012770	Tfasc_v1.15761-RA, Tlei_v1.22494-RA	Inf	0,0318	0,0003	0,01374	metallo-hydrolase/oxidoreductase superfamily protein
OG0011786	Tfasc_v1.12421-RA, Tlei_v1.10008-RA	9,2985	0,0189	0,002	0,00008	U-box_domain-containing_protein
OG0008528	Tfasc_v1.04577-RA, Tlei_v1.08412-RA	8,17	0,0322	0,0039	0,01292	hypothetical protein ACMD2_08159
OG0015603	Tfasc_v1.14595-RA, Tlei_v1.21364-RA	8,0697	0,0228	0,0028	0,01575	anaphase-promoting complex subunit CDC27
OG0008124	Tfasc_v1.15280-RB, Tlei_v1.22017-RA	3,1536	0,0238	0,0075	0,01368	uncharacterized protein LOC109710130 isoform X1
OG0009004	Tfasc_v1.16390-RA, Tlei_v1.06962-RA	3,1379	0,0271	0,0087	0,01148	Hydroquinone glycosyltransferase
OG0019176	Tfasc_v1.13382-RA, Tlei_v1.18537-RA	2,8534	0,0466	0,0163	0,00561	uncharacterized protein LOC109718296
OG0008977	Tfasc_v1.16327-RA, Tlei_v1.06894-RA	2,6204	0,0281	0,0107	0,00512	glutamate receptor 2.8-like
OG0010014	Tfasc_v1.26649-RA, Tlei_v1.28282-RA	2,0042	0,0701	0,035	0,00603	Glycerophosphodiester phosphodiesterase GDPDL7
1:1:2 orthologues						
OG0003849	Tfasc_v1.03397-RA, Tlei_v1.09369-RA	Inf	0,0093	0,0001	0,00697	kinesin-like protein KIN-10C
	Tfasc_v1.03397-RA, Tlei_v1.09368-RA	4,1684	0,0093	0,0022	0,09607	
1:2:1 orthologues						
OG0003795	Tfasc_v1.16138-RA, Tlei_v1.06694-RA	6,2573	0,0648	0,0104	0,02611	Ubiquitin-conjugating enzyme E2
	Tfasc_v1.16140-RA, Tlei_v1.06694-RA	0,2438	0,0334	0,1368	0,03386	
OG0004404	Tfasc_v1.08653-RA, Tlei_v1.17093-RA	3,9913	0,0945	0,0237	0,02787	protein ECERIFERUM 1-like
	Tfasc_v1.21655-RA, Tlei_v1.17093-RA	0,1733	0,0051	0,0297	0,00059	

Supplementary Table S7: Description of gene clusters inferred in co-expression analyses from maSigPro

Cluster	Number of genes	Genes of interest	GO terms of interest
1	87	Tfasc_v1.23066: protein LNK1-like;<br>Tfasc_v1.19779: Dicarboxylate transporter 1, chloroplastic;<br>Tfasc_v1.08739: mitochondrial uncoupling protein 5-like;<br>Tfasc_v1.00158: ABC transporter G family member 5-like;<br>Tfasc_v1.01823: Protein REVEILLE 1 [1];<br>Tfasc_v1.03880: Transcription factor PCF2 [2];<br>Tfasc_v1.06881*: protein LHY-like isoform X1 [2];<br>Tfasc_v1.09028: aluminum-activated malate transporter 9-like isoform X1 [1]	GO:1902356: oxaloacetate(2-) transmembrane transport;<br>GO:0071423: malate transmembrane transport;<br>GO:1902074: response to salt;<br>GO:0071472: cellular response to salt stress;<br>GO:1902584: positive regulation of response to water deprivation;<br>GO:1901002: positive regulation of response to salt stress;<br>GO:0015131: oxaloacetate transmembrane transporter activity;<br>GO:0015140: malate transmembrane transporter activity
2	134	Tfasc_v1.25154: V-type proton ATPase catalytic subunit A [3];<br>Tfasc_v1.11797: protein XAP5 CIRCADIAN TIMEKEEPER [3];<br>Tfasc_v1.21051: V-type proton ATPase subunit e1;<br>Tfasc_v1.15469*: Acyl-coenzyme A thioesterase [2];<br>Tfasc_v1.09221: Pyrophosphate--fructose 6-phosphate 1-phosphotransferase subunit alpha;<br>Tfasc_v1.12690*: pentatricopeptide repeat-containing protein At1g09900-like [2];	GO:0033179: proton-transporting V-type ATPase, V0 domain;<br>GO:0047334: diphosphate-fructose-6-phosphate 1-phosphotransferase activity
3	38	Tfasc_v1.09150: protein HOMOLOG OF MAMMALIAN LYST-INTERACTING PROTEIN 5;<br>Tfasc_v1.03774: probable aquaporin PIP2-6 [4];<br>Tfasc_v1.14176*: long chain acyl-CoA synthetase 4-like [2];<br>Tfasc_v1.24696: V-type proton ATPase subunit H-like [5];<br>Tfasc_v1.25341: pyrophosphate-energized vacuolar membrane proton pump [3];<br>Tfasc_v1.28097: F-box/kelch-repeat protein SKIP25 [2];<br>Tfasc_v1.14652-RA: protein GIGANTEA [3]	GO:0007623: circadian rhythm;<br>GO:0010378: temperature compensation of the circadian clock;<br>GO:0046323: glucose import;<br>GO:0009637: response to blue light;<br>GO:0048578: positive regulation of long-day photoperiodism, flowering;<br>GO:0071482: cellular response to light stimulus
4	99	Tfasc_v1.09150: protein HOMOLOG OF MAMMALIAN LYST-INTERACTING PROTEIN 5;<br>Tfasc_v1.03774: probable aquaporin PIP2-6 [4];<br>Tfasc_v1.14176: long chain acyl-CoA synthetase 4-like [2];<br>Tfasc_v1.24696: V-type proton ATPase subunit H-like [5];<br>Tfasc_v1.25341: pyrophosphate-energized vacuolar membrane proton pump [3];<br>Tfasc_v1.28097: F-box/kelch-repeat protein SKIP25 [2]	GO:1903335: regulation of vacuolar transport;<br>GO:0000221: vacuolar proton-transporting V-type ATPase, V1 domain
5	209	Tfasc_v1.01733: V-type proton ATPase 16 kDa proteolipid subunit [2];<br>Tfasc_v1.16595: soluble starch synthase I;<br>Tfasc_v1.27353: beta-amylase 3 [10]<br>Tfasc_v1.26941: glucose-6-phosphate/phosphate translocator [6];<br>Tfasc_v1.17464: fructose-bisphosphate aldolase 1, cytoplasmic [6]<br>Tfasc_v1.21461: Pyruvate kinase, cytosolic isozyme [2][6];<br>Tfasc_v1.16311: Phosphoenolpyruvate carboxylase (PEPC) [7];<br>Tfasc_v1.03126: malate dehydrogenase [7];<br>Tfasc_v1.03128: PEPC kinase [7];<br>Tfasc_v1.06749: phosphoglucan phosphatase DSP4, amyloplastic isoform X1;<br>Tfasc_v1.09378: V-type proton ATPase subunit d2 [2][5];<br>Tfasc_v1.20370: ATP-dependent 6-phosphofructokinase 5, chloroplastic;<br>Tfasc_v1.24086: ATP-dependent 6-phosphofructokinase 3;<br>Tfasc_v1.17156: aconitate hydratase;<br>Tfasc_v1.04514: glucose-6-phosphate isomerase, cytosolic;<br>Tfasc_v1.07899: phosphoenolpyruvate carboxykinase (ATP);<br>Tfasc_v1.26280: protein MAEA homolog;<br>Tfasc_v1.27863: pyruvate decarboxylase 1;<br>Tfasc_v1.03307: V-type proton ATPase subunit a3 [5];<br>Tfasc_v1.00598: nuclear pore complex protein NUP50A-like [2];<br>Tfasc_v1.01511: late embryogenesis abundant protein Lea5-like [8];<br>Tfasc_v1.03613*: probable pyruvate, phosphate dikinase regulatory protein, chloroplastic [2][7];<br>Tfasc_v1.14299: late embryogenesis abundant protein group 8 protein [8];<br>Tfasc_v1.14724: V-type proton ATPase subunit B 2 [3];<br>Tfasc_v1.15525: protein YLS7-like [2];<br>Tfasc_v1.16080: V-type proton ATPase subunit c''2 [2][5];<br>Tfasc_v1.17712: V-type proton ATPase subunit C [3];<br>Tfasc_v1.18209: V-type proton ATPase subunit G 1-like [3];<br>Tfasc_v1.19718: F-box protein At1g55000 [2];<br>Tfasc_v1.19742: V-type proton ATPase subunit H [5]<br>Tfasc_v1.26860: gamma-aminobutyrate transaminase 1, mitochondrial [2];<br>Tfasc_v1.28537: protein FD [9];<br>Tfasc_v1.28610: uncharacterized protein LOC109727440 [9]	GO:0005983: starch catabolic process;<br>GO:0061615: glycolytic process through fructose-6-phosphate;<br>GO:0006099: tricarboxylic acid cycle;<br>GO:0006094: gluconeogenesis;<br>GO:0006002: fructose 6-phosphate metabolic process;<br>GO:0045721: negative regulation of gluconeogenesis;<br>GO:0007035: vacuolar acidification;<br>GO:0006107: oxaloacetate metabolic process;<br>GO:0004737: pyruvate decarboxylase activity;<br>GO:0004612: phosphoenolpyruvate carboxykinase (ATP) activity;<br>GO:0008964: phosphoenolpyruvate carboxylase activity;<br>GO:0046961: proton-transporting ATPase activity, rotational mechanism;<br>GO:0047780: citrate dehydratase activity;<br>GO:0004347: glucose-6-phosphate isomerase activity
6	144	Tfasc_v1.20354: sucrose transport protein SUT2-like [3];<br>Tfasc_v1.25107: VMA21-like domain-containing protein;<br>Tfasc_v1.06152*: Protein EDS1L [2];<br>Tfasc_v1.08627: WAT1-related protein [2];<br>Tfasc_v1.16433: arabinogalactan peptide 16-like [2];<br>Tfasc_v1.24231*: replication stress response regulator SDE2 [2];<br>Tfasc_v1.30364*: protein FLX-like 4 [2];<br>Tfasc_v1.25107: VMA21-like domain-containing protein;<br>Tfasc_v1.26899: obg-like ATPase 1;<br>Tfasc_v1.25528: granule-bound starch synthase	GO:0015770: sucrose transport;<br>GO:0015768: maltose transport;<br>GO:0070072: vacuolar proton-transporting V-type ATPase complex assembly;<br>GO:1901001: negative regulation of response to salt stress;<br>GO:0005364: maltose:proton symporter activity;<br>GO:0003985: acetyl-CoA C-acetyltransferase activity;<br>GO:0004373: glycogen (starch) synthase activity
7	196	Tfasc_v1.30467: isocitrate dehydrogenase [NAD] catalytic subunit 5, mitochondrial;<br>Tfasc_v1.21339: catalase isozyme 1;<br>Tfasc_v1.12645: flavonoid 3',5'-hydroxylase 2-like [2];<br>Tfasc_v1.25055*: berberine bridge enzyme-like 18 [2];<br>Tfasc_v1.29220: ABC transporter C family member 4-like [3]	GO:1902074: response to salt;<br>GO:1900034: regulation of cellular response to heat;<br>GO:0004449: isocitrate dehydrogenase (NAD+) activity
		* Candidate gene for adaptive sequence evolution in CAM/C3 shifts reported in [2]	

Citations

[1]	Wai, Ching M. et al. 2017. "Temporal and Spatial Transcriptomic and MicroRNA Dynamics of CAM Photosynthesis in Pineapple." The Plant Journal 92(1): 19–30.
[2]	De La Harpe, M. et al. 2020. "Genomic footprints of repeated evolution of CAM photosynthesis in a Neotropical species radiation." Plant, Cell and Environment 43(12): 2987-3001.
[3]	McClung, C. Robertson. 2006. "Plant Circadian Rhythms." The Plant Cell 18(4): 792–803.
[4]	Vera-Estrella, R. et al. 2012. "Day/Night Regulation of Aquaporins during the CAM Cycle in Mesembryanthemum Crystallinum." Plant, Cell & Environment 35(3): 485–501.
[5]	Cosentino, Cristian et al. 2013. "Proteomic Analysis of Mesembryanthemum Crystallinum Leaf Microsomal Fractions Finds an Imbalance in V-ATPase Stoichiometry during the Salt-Induced Transition from C3 to CAM." Biochemical Journal 450(2): 407–15.
[6]	Cushman, John C. et al. 2008. "Large-Scale MRNA Expression Profiling in the Common Ice Plant, Mesembryanthemum Crystallinum, Performing C3 Photosynthesis and Crassulacean Acid Metabolism (CAM)." Journal of Experimental Botany 59(7): 1875–94.
[7]	Ming, Ray et al. 2015. "The Pineapple Genome and the Evolution of CAM Photosynthesis." Nature Genetics 47(12): 1435–42.
[8]	Xiao, B., et al. 2007. "Over-Expression of a LEA Gene in Rice Improves Drought Resistance under the Field Conditions." Theoretical and Applied Genetics 115(1): 35–46.
[9]	Christin, Pascal-Antoine et al. 2014. "Shared Origins of a Key Enzyme during the Evolution of C4 and CAM Metabolism." Journal of Experimental Botany 65(13): 3609–21.
[10]	Sean E. Weise and others, The role of transitory starch in C3, CAM, and C4 metabolism and opportunities for engineering leaf starch accumulation, Journal of Experimental Botany, Volume 62, Issue 9, May 2011, Pages 3109–3118, https://doi.org/10.1093/jxb/err035

Supplementary Table S8: Per-kb frequency of four promotor motifs associated with transcription factors of the circadian clock: The Morning Element (MOE), the Evening Element (EE), the CCA1-Binding Site (CBS) and the G-box motif. The difference in frequency reflects the change in the per-kb frequency of a motif in the target group compared to the background per-kb frequency (in percentage). Negative values reflect a decreased frequency of a motive compared to the background, where as positive values show an increase. Motifs with > 10 % frequency increase in a target group are shown in bold. Significance of per-gene motif frequency difference was tested with the Mann-Whitney U Test: *p-value < 0.05, **p-value < 0.01, ***p-value < 1E-03. Exact p-values and other details on the statistical testing can be found in Supplementary Data Set 2.

Background per-kb frequency of circadian motifs					
Group	Species	MOE	EE	CBS	G-box
Non-DE genes	*T. leiboldiana*	0,172479	0,117555	0,105135	0,193086
	T. fasciculata	0,161376	0,093558	0,105319	0,184369
**Difference in per-kb frequency of circadian motifs in percentage (%)**					
	Species	MOE	EE	CBS	G-box
All DE genes	*T. leiboldiana*	-4,18	**15,78**	9,65	-3,25
	T. fasciculata	-2,93	**18,54**	**18,39**	0,93
cluster 1	*T. fasciculata*	-13,72	8,24	14,18	**81,94****
cluster 2	*T. fasciculata*	0,49	5,92	-18,74	-26,70
cluster 3	*T. fasciculata*	-15,50	**207,70****	**72,64**	-1,38
cluster 4	*T. fasciculata*	**25,98**	-0,16	**35,64**	-19,54
cluster 5	*T. fasciculata*	-10,10	**49,53**	**45,13**	-4,45
cluster 6	*T. fasciculata*	1,59	0,75	-49,41	-17,75
cluster 7	*T. fasciculata*	-8,33	-17,78	**43,27***	12,33

Supplementary Table S9: Occurrence of circadian TF-binding motifs in 2-kb (* and 3-kb) upstream regions of core CAM genes and their related homologs in *T. fasciculata* and *T. leiboldiana*. Genes marked with an ✦ were differentially expressed.

Gene_ID	MOE	EE	CBS	G-Box	Orthogroup	Function
Tfasc_v1.29951-RA	0	2	0	2	OG0009306	α-Carbonic anhydrase
Tlei_v1.20941-RA	0	1	0	1		
Tfasc_v1.09713-RA	0	1	0	0	OG0008211	β-Carbonic anhydrase 2
Tlei_v1.24345-RA	0	1	0	0		
Tfasc_v1.04038-RA	0	1*	0	0	OG0003207	β-Carbonic anhydrase 5
Tfasc_v1.08735-RA	0	0	0	1		
Tlei_v1.13295-RA	0	0	0	1		
Tfasc_v1.07635-RA	1	0	1*	0	OG0001548	Malate dehydrogenase
Tfasc_v1.03126-RA ✦	1	0	0	0		
Tlei_v1.15644-RA	1*	0	1*	0		
Tlei_v1.03837-RA ✦	1	0	0	0		
Tfasc_v1.16311-RA ✦	0	0	0	0	OG0001504	PEPC 1
Tlei_v1.06877-RA ✦	0	0	0	0		
Tfasc_v1.09872-RA	0	0	0	0		
Tlei_v1.24540-RA	1	0	0	0		
Tfasc_v1.17028-RA	0	1*	0	1	OG0014303	PEPC 2
Tlei_v1.07896-RA	0	1	0	1		
Tfasc_v1.07899-RA ✦	3	0	0	0	OG0019125	PEPCK
Tlei_v1.15895-RA ✦	1*	0	0	0		
Tfasc_v1.21696-RA	1	0	0	0	OG0004399	PPCK 1
Tlei_v1.17058-RA	1	1	0	0		
Tfasc_v1.03128-RA ✦	0	0	1*	0	OG0013607	PPCK 2
Tlei_v1.03835-RA ✦	0	0	1	2		

Supplementary Table S10: Composition of transcription factor families (identified with three different appraches) by relative size between *T. fasciculata* (CAM) and *T. leiboldiana* (C3). A chi-square test of independence was applied to test the significance of composition changes in 2x2 contingency tables for each category between TF and Non-TF families. Non-TF values here are shown relative to the TF families (Merged) column. Significant p-values of both tests are reported as: *0.05-0.01, **0.01-0.0001, ***0.0001-0. Below, the compisition of differentially expressed TF families based on DGE analyses performed on the *T. fasciculata* (F) and *T. leiboldiana* (L) genomes. Fisher's Exact Test on 2x2 contingency tables resulted in non-significant p-values (p-value > 0.05). Exact p-values and other details on the statistical testing can be found in Supplementary Data Set 2.

All identified Transcription Factor orthogroups versus all non-TF orthogroups

	Non-TF families	TF families (Merged)	TF families (homologous to *A. comosus* TFs)	TF families (identified with IPR domains)
Total orthogroup count	12,925	1,359	1,057	1,168
% of 1:1 OGs	75.33	85.43 ***	88.10 ***	84.67 ***
% of F:L OGs (F > L)	4.89	2.87 **	1.61 ***	3.33 *
% of F:L OGs (F < L)	3.12	2.21	1.80 *	2.31 - ns
% of F:L OGs (F = L)	3.00	1.77 **	1.61 **	1.88 *
% of F:0 OGs	6.33	3.24 ***	3.22 ***	3.25 ***
% of 0:L Ogs	7.30	4.49 ***	3.69 ***	4.53 **

DE Transcription Factor orthogroups versus non-DE orthogroups

	Non-DE families	DE TF families (F)	DE TF families (L)
Total orthogroup count	16,147	37	37
% of 1:1 OGs	87.27	83.78	78.38
% of F:L OGs (F > L)	5.67	8.11	8.11
% of F:L OGs (F < L)	3.61	2.70	8.11
% of F:L OGs (F = L)	3.48	5.41	5.41

Supplementary Table S11: List of DE genes related to CAM, starch metabolism and gluconeogenesis with a TE insertion rate higher than twice the genome-wide average in *T. leiboldiana* and *T. fasciculata.* Genes of the two species belonging to the same orthogroup are marked with the same symbol in the first column. When only one orthologous copy of the gene exists in the other species, we report the difference in the number of TE insertions between species as (# insertion in *T. fasciculata* - # insertion in *T. leiboldiana*). Therefore, a positive value reflects a gene with more TE insertions in *T. fasciculata* than *T. leiboldiana*, while a negative value shows the opposite.

Gene ID	TE insertion count (intronic / intronic + 3 kb upstream)	Orthogroup	# copies in *A. comosus*	# copies in *T.fasciculata*	# copies in *T.leiboldiana*	ΔTE insertions (F - L)	Description
In T. fasciculata							
Tfasc_v1.24696-RA	9 / 19	OG0000539	2	8	3	NA	V-type proton ATPase subunit H-like (proton pump, vacuolar transport)
Tfasc_v1.29220-RA	6 / 6	OG0001699	2	2	2	NA	ABC transporter C family member 4-like (stomatal movement, circadian rhythm) [1][2]
Tfasc_v1.22969-RA ✦	10 / 10	OG0011563	1	1	1	3	glucose-1-phosphate adenylyltransferase large subunit 3, chloroplastic/amyloplastic (starch synthesis)
Tfasc_v1.30467-RA	7 / 9	OG0005172	1	2	1	4	isocitrate dehydrogenase [NAD] catalytic subunit 5, mitochondrial (alternative CO_2 fixator in CAM) [3]
Tfasc_v1.08627-RA	19 / 24	OG0007038	1	1	1	19	WAT1-related protein (secondary cell wall growth) [4]
Tfasc_v1.09028-RA	10 / 11	OG0007222	1	1	1	4	aluminum-activated malate transporter 9-like isoform X1 (malate transporter)
Tfasc_v1.17712-RA ✢	7 / 19	OG0004832	2	1	1	-1	V-type proton ATPase subunit C (proton pump, vacuolar transport)
Tfasc_v1.12645-RA ╳	7 / 10	OG0016000	1	1	1	0	flavonoid 3',5'-hydroxylase 2-like (anthocyanin pathway) [5]
Tfasc_v1.14652-RA ✯	12 / 16	OG0014957	1	1	1	-2	protein GIGANTEA (circadian rhythm, stomatal opening) [7][8]
In T. leiboldiana							
Tlei_v1.10209-RA ╳	7 / 19	OG0016000	1	1	1	0	flavonoid 3',5'-hydroxylase 2-like (anthocyanin pathway) [5]
Tlei_v1.21413-RA ✯	14 / 16	OG0014957	1	1	1	-2	protein GIGANTEA (circadian rhythm, stomatal opening) [7][8]
Tlei_v1.16158-RA	23 / 36	OG0015868	1	1	1	-14	Beta-fructofuranosidase, insoluble isoenzyme 3 (sugar metabolism)
Tlei_v1.04787-RA ✢	8 / 18	OG0004832	2	1	1	-1	V-type proton ATPase subunit C (proton pump, vacuolar transport)
Tlei_v1.02834-RA	15 / 25	OG0002339	2	1	1	1	sugar transporter ERD6-like
Tlei_v1.23795-RA ✦	7 / 7	OG0011563	1	1	1	3	glucose-1-phosphate adenylyltransferase large subunit 3, chloroplastic/amyloplastic (starch synthesis)

[1] Wai, C.M., VanBuren, R., Zhang, J., Huang, L., Miao, W., Edger, P.P., Yim, W.C., Priest, H.D., Meyers, B.C., Mockler, T., Smith, J.A.C., Cushman, J.C. and Ming, R. (2017), Temporal and spatial transcriptomic and microRNA dynamics of CAM photosynthesis in pineapple. Plant J, 92: 19-30. https://doi.org/10.1111/tpj.13630

[2] Klein, M., Geisler, M., Suh, S.J., Kolukisaoglu, H.Ü., Azevedo, L., Plaza, S., Curtis, M.D., Richter, A., Weder, B., Schulz, B. and Martinoia, E. (2004), Disruption of AtMRP4, a guard cell plasma membrane ABCC-type ABC transporter, leads to deregulation of stomatal opening and increased drought susceptibility. The Plant Journal, 39: 219-236. https://doi.org/10.1111/j.1365-

[3] Nadine Töpfer, Thomas Braam, Sanu Shameer, R. George Ratcliffe, Lee J. Sweetlove, Alternative Crassulacean Acid Metabolism Modes Provide Environment-Specific Water-Saving Benefits in a Leaf Metabolic Model, The Plant Cell, Volume 32, Issue 12, December 2020, Pages 3689–3705, https://doi.org/10.1105/tpc.20.00132

[4] Ranocha P, Dima O, Nagy R, et al. Arabidopsis WAT1 is a vacuolar auxin transport facilitator required for auxin homoeostasis. Nat Commun. 2013;4:2625. doi:10.1038/ncomms3625

[5] Tanaka, Y., & Brugliera, F. (2013). Flower colour and cytochromes P450. Philosophical Transactions of the Royal Society B: Biological Sciences, 368(1612), 20120432.

[6] Cushman, John C. et al. 2008. "Large-Scale MRNA Expression Profiling in the Common Ice Plant, Mesembryanthemum Crystallinum, Performing C3 Photosynthesis and Crassulacean Acid Metabolism (CAM)." Journal of Experimental Botany 59(7): 1875–94.

[7] Ando E, Ohnishi M, Wang Y, et al. TWIN SISTER OF FT, GIGANTEA, and CONSTANS have a positive but indirect effect on blue light-induced stomatal opening in Arabidopsis. Plant Physiol. 2013;162(3):1529-1538. doi:10.1104/pp.113.217984

[8] Fornara, F., Panigrahi, K. C., Gissot, L., Sauerbrunn, N., Rühl, M., Jarillo, J. A., & Coupland, G. (2009). Arabidopsis DOF transcription factors act redundantly to reduce CONSTANS expression and are essential for a photoperiodic flowering response. Developmental cell, 17(1), 75-86.

Supplementary Table S12: InterPro codes used to identify transcription factor families in *T. fasciculata* and *T. leiboldiana*.

IPR Code	IPR Domain name	TF family
IPR001471	AP2/ERF domain	AP2, ERF, RAV
IPR010525	Auxin Response Factor domain	ARF
IPR003340	B3 DNA Binding domain	B3, RAV
IPR011598	Myc-type, basic helix-loop-helix (bHLH) domain	bHLH
IPR004827	Basic-leucine zipper domain	bZIP
IPR013087	Zinc finger C2H2-type	C2H2
IPR000571	Zinc finger, CCCH-type	C3H
IPR003851	Zinc finger, Dof-type	Dof
IPR004330	FAR1 DNA binding domain	FAR1
IPR025756	MYB-CC type transcription factor, LHEQLE-containing domain	G2-like
IPR005202	Transcription factor GRAS	GRAS
IPR001356	Homeobox domain	HB-PHD, HD-ZIP, TALE, WOX
IPR003106	Leucine zipper, homeobox-associated	HD-ZIP
IPR000232	Heat shock factor (HSF)-type, DNA-binding	HSF
IPR004883	Lateral organ boundaries, LOB	LBD
IPR002100	Transcription factor, MADS-box	M-type MADS
IPR002487	Transcription factor, K-box	MIKC MADS
IPR001005	SANT/Myb domain	MYB, MYB-Related, ARR-B
IPR003441	NAC domain	NAC
IPR003657	WRKY domain	WRKY
IPR006455	Homeodomain, ZF-HD class	ZF-HD

3. Supplementary Notes

Note 1: Genome size and karyotype of *T. fasciculata* and *T. leiboldiana*

The genome size of both specimens used for *de novo* assembly was measured with flow cytometry (See Methods) estimating genome sizes of approximately 790 and 1,130 Mb for *T. fasciculata* and *T. leiboldiana* respectively (Fig. S2). These estimates are slightly higher than those obtained computationally with a kmer-based approach implemented in findGSE (Sun et al., 2018) (k = 21 ,701 and 1,125 Mb respectively, Fig. S3); but deviations of genome size in computational approaches have been reported frequently (Al-Qurainy et al., 2021; Pflug et al., 2020; Elliott and Gregory, 2015). We also obtained a karyotype for both species using root material (See Methods). We observe a change in karyotype between the two species resulting in a reduction by six chromosome pairs in *T. leiboldiana* compared to *T. fasciculata*, which carries the base karyotype of 2n = 50 encountered in *Tillandsioideae* (Brown and Gilmartin, 1989) (Fig. S5). This is in accordance with chromosome counts reported by Brown and Gilmartin (1989).

Note 2: Pre-assembly estimation of per-accession heterozygosity

Heterozygosity estimates of the chosen accessions along with several other candidate accessions from the Botanical Gardens of the University of Vienna were obtained with short-read Illumina data before *de novo* assembly, with the aim to select accessions with lowest heterozygosity and to make adjustments during *de novo* assembly to account for potentially elevated rates of heterozygosity. This short-read data was later used for polishing purposes and the sequencing details can be found in the Material and Methods section.

Heterozygosity estimates were obtained by two approaches: a k-mer based approach and a reference-based approach. For the k-mer based approach, findGSE (Sun et al., 2018) was used with a k = 21 to obtain k-mer peaks of heterozygosity. For the reference-based approach, reads were trimmed with TrimmOmatic (Bolger et al., 2014) and mapped with GSNAP (Wu et al., 2016) to the *Tillandsia adpressiflora*

pseudoreference built by De la Harpe and colleagues (2020). After filtering for low mapping quality and marking duplicates, variants were called for all accessions using *freebayes* (Garrison and Marth, 2012). Variants with an individual depth under 5 and above 45 were removed and no missing data was retained. Then, heterozygous sites per 1000 mappable sites were counted with a custom-made python script, filtering for allele balance between 0.25 and 0.75. This yielded between 51,000 and 58,000 windows, translating to roughly a 50 Mb portion of the genome. We expect these windows to be enriched for genic and other conserved regions, therefore resulting in an underestimate, though for relative comparisons, we regard this approach as valid.

Both the k-mer (Fig. S3) and reference-based approach (Fig. S15) showed that heterozygosity is elevated in *T. fasciculata* compared to *T. leiboldiana*. This result is consistent with the nucleotide diversity estimates reported by (Yardeni et al., 2021) based on sequencing of 1776 targeted loci in several *Tillandsia* species, including *T. leiboldiana* (π_S=5.7x10^{-3}) and *T. fasciculata* (π_S=8.1x10^{-3}). The relatively moderate levels of diversity in these species helped us to obtain assemblies with such a remarkable contiguity (Table S2), despite their very high repetitive content (see Supplementary Note 4).

Note 3: Identifying main scaffolds in de novo assembly

After scaffolding with Hi-C data, the resulting *de novo* assemblies contained a total of 2,321 and 10,443 scaffolds (> 1 kb) for *T. fasciculata* and *T. leiboldiana* respectively. However, more than 99 % of one-to-one orthologous gene pairs are located on the 25 and 26 largest scaffolds of both assemblies respectively, while 90.7 % and 87.6 % of all gene models are on these scaffolds. Therefore, the remaining scaffolds mainly consist of repetitive content, virtually corresponding to short, duplicated regions in the assembly. The mean proportion of repetitive content in the remaining scaffolds is indeed much higher than in main scaffolds (94.9 % and 91 % in small scaffolds of *T. fasciculata* and *T. leiboldiana*, versus 65.5 and 77.1% in the “main” scaffolds (see main text)). In addition, these “main” scaffolds contain the vast majority of the assembly (72 % and 75.5 % of the total assembly length). While the sizes of the longest 25 and 24 scaffolds are over 1 Mb, other scaffold sizes steeply decline afterwards (Fig. S4). Given this, we decided to regard

these scaffolds as representative for the respective genomes and excluded all secondary scaffolds from downstream analyses from this point onwards. Though scaffolds 26 and 25 in *T. leiboldiana* are smaller than 1 Mb, they contain a substantial number of orthologous genes (Fig. S4) and were therefore maintained in all analyses.

Note 4: On the spatial distribution of GC and TE content in bromeliad genomes

After finding that GC and genic content were negatively correlated in both *Tillandsia* genomes (See Results), we decided to study the link between GC content and repetitive content in all bromeliad genomes available to us at the time (*A. comosus*, *T. fasciculata* and *T. leiboldiana*) Using softmasked versions for all three genomes (see Materials and Methods), we computed the proportion of soft-masked bases across 100 kb windows. We also computed the overall GC content (considering both softmasked and non-softmasked positions), the GC content for soft-masked bases only, and the GC content for non-softmasked bases in the same windows. Based on this, the average TE content was estimated to be of 36.7 %, 67.9 % and 79.1% for *A. comosus*, *T. fasciculata* and *T. leiboldiana*, respectively. These three genomes therefore represent a gradient regarding the amount of TEs. After having reported the relatively well conserved synteny (Fig. 3c), we were able to estimate the evolution of the repetitive, GC and genic chromosomal landscapes across syntenic chromosomes.

We selected three examples of syntenic triplets considering scaffolds with no main chromosomal rearrangements: triplets A.com 3 / T.fas 4 / T.lei 1 (Fig. 3b), A.com 6 / T.fas 11 / Tlei. 15 and A.com 11 / T.fas 12 / T.lei 5 (Fig. S6). We then considered a relative position of each window on the scaffold (window position/scaffold length) to account for the difference in length of the syntenic chromosomes in the three assemblies. From this visualisation, it became clear that the GC content landscape is largely shaped by TE dynamics in the three genomes, since the GC% at non-repetitive content show no to little variation across the scaffold, except in *T. leiboldiana*. This contributed to building large GC-rich isochores in centromeric regions. Note here that our *de novo* TE libraries are not necessarily exhaustive and therefore a part of the repetitive content may have remained non-softmasked, which could partly or completely explain the pattern observed for GC at

non-softmasked position in *T. leiboldiana*. Since this pattern is observed in all three species, regardless of the difference in repetitive content, this may be a family-wide phenomenon.

Note 5: Identifying large-scale rearrangements between *T. fasciculata* and *T. leiboldiana*

Large-scale rearrangements were first perceived in the synteny analysis of the two assemblies. These were further investigated by whole-genome alignment of the two assemblies to each other, and to *A. comosus*, using nucmer (Delcher et al., 2002) and visualised with Dot (https://github.com/dnanexus/dot).

Large rearrangements that were identified between *T. fasciculata* and *T. leiboldiana* were further investigated by performing LastZ alignments (Harris, 2007) of soft-masked genomes. LastZ was run with the following settings: --notransition, --step=10, --gapped, --chain, --gfextend, --format=maf. Local alignments were filtered by the 95th identity and length quartile as implemented in (Leroy et al., 2021). Additionally, alignments were filtered by uniqueness with a custom-made python script, by removing all alignments with more than a 90 % overlap. Final local alignments were visualised for each scaffold as in Leroy et al (2021). All scripts are available at: https://shorturl.at/xLS15.

The breakpoint area of confirmed rearrangements was defined as the region between the last alignment of a given scaffold and the first alignment of another scaffold. Rearrangements were then finally confirmed by investigating the alignment of long-read PacBio data to the assembly of the same species. Whenever no clear break could be identified in the long-read alignment within the breakpoint area, the rearrangement was considered as confirmed.

With these methods, we described three potential large-scale rearrangements. Scaffold 14 in *T. leiboldiana* could be a fusion of scaffolds 17 and 25 in *T. fasciculata*, or scaffolds 17 and 25 could be the result of a fission in a reversed scenario (Fig. S7a). We also detected two potential translocations (Fig. S7b for Translocation 1 and Fig. S7c for Translocation 2). All breakpoints were confirmed by alignment of raw PacBio reads, except for breakpoint 1 on scaffold 13 of *T. fasciculata* of Translocation 1 (Fig. S7b).

However, breakpoint 2 on this scaffold was confirmed, which led us to maintain the translocation as a candidate rearrangement. For several scaffolds, local alignments were too sparse to determine a clear breakpoint (See Fig. S7). To see the PacBio alignment at each breakpoint, see the supplementary PDF file “Tfas_Tlei_rearrangements.pdf” on our github repository.

We studied the effects of large-scale rearrangement on the genomic distribution of d_N/d_S values and, separately, on DE genes (See SI Note 11) to understand if there is a link between chromosomal and functional evolution in *Tillandsia*. We did this by testing whether the distribution of d_N/d_S values in any of the rearranged chromosomes deviated from that of non-rearranged chromosomes (See Methods). Of the nine scaffolds involved in the three reported rearrangements, only two had a d_N/d_S distribution significantly deviating from that of non-rearranged chromosomes (scaffold 13 in *T. fasciculata* and scaffold 19 in *T. leiboldiana*, see Fig. S8b). The d_N/d_S values in these scaffolds, which are both involved in Translocation 1, were ever so slightly reduced compared to non-rearranged scaffolds (median scaffold 13 = 0.3197, other scaffolds in *T. fasciculata* = 0.3569; median scaffold 19 = 0.3326, other scaffolds in *T. leiboldiana* = 0.3591). An overall reduction in chromosome-wide d_N/d_S values can be expected in rearranged chromosomes due to an increase in linkage disequilibrium resulting from recombination suppression, which in turn increases background selection (Mérot et al., 2020; Cicconardi et al., 2021). This can have important implications for both adaptation and speciation, as functional loci may be under increased selective constraint, and selection against introgression may also become stronger (Cicconardi et al., 2021). However, the significance of this reduction in d_N/d_S is very slight and only visible in two of nine rearranged scaffolds. Combined with our results on DE gene distribution across the genome, which show no signal of rearrangements playing a role in spatial distribution of ecologically relevant genes (SI Note 12), we are cautious in heralding large-scale rearrangement as a key driving force of ecological diversification in *Tillandsia* until additional supporting evidence becomes available (See SI Note 14).

Note 6: Selecting rapidly evolving gene families

To better understand the distribution of gene size differences, the log-ratio was taken of *T. fasciculata* to *T. leiboldiana* gene counts, and the overall mean log-ratio was subtracted to correct for background rates of gene loss or duplication. Orthogroups were ranked by corrected log-ratios and the top and bottom 2 % were then selected for further analysis. Due to the relatively large proportion of one-to-one relationships (79 %) among orthogroups, all orthogroups with a family size change between *T. fasciculata* and *T. leiboldiana* were included in the top 2 % of gene changes and therefore selected for GO term enrichment, which was performed separately for orthogroups with gene count larger in *T. fasciculata* (916 orthogroups) and larger in *T. leiboldiana* (583 orthogroups).

Note 7: Detailed description of candidate genes for positive selection

Our d_N/d_S calculations pointed at 13 single-copy and 3 multi-copy genes exhibiting signatures of divergent selection between *T. fasciculata* and *T. leiboldiana*. The most relevant genes have been described in the main text (fig. 4c) and in Table S6, but here we provide more information on all candidate genes, which could be interesting for future work to investigate speciation genes in *Tillandsia*.

Among single-copy candidates, we found a Jacalin-related lectin (JLR, OG0002972), which are often associated with biotic and abiotic stimuli, though their biological function is largely unknown. In wheat, a mannose-specific JLR has been identified as a component of the defence system (Xiang et al., 2011). In rice, a JLR has been described as playing a role in salt stress response (Zhang et al., 2000).

Orthogroup OG0005000 codes for a cupredoxin (cupredoxin cucumber peeling-like). Cupredoxins are small proteins containing a copper centre which function as electron transfer shuttles between redox partners, but their more specific biological function is largely unknown (Guss et al., 1996). However, they tend to play a role in respiration, photosynthesis and metabolism (Choi and Davidson, 2011), and therefore represent another interesting candidate for further investigations.

Another candidate for adaptive sequence evolution was a mitochondrial prohibitin-3 (OG0006253), a subunit of the prohibitin complex. While the exact mechanism of prohibitin is unknown, it has been associated with mitochondrial biogenesis in *Nicotiana benthamiana* (Ahn et al., 2006) and more specifically protection against salt stress in *Arabidopsis thaliana* (Wang et al., 2012).

We recovered a hydroquinone glycosyltransferase (OG0009004), a broad-spectrum glycosyltransferase involved in the secondary metabolism of many phenolic compounds and xenobiotics(Hefner et al., 2002).

Orthogroup OG0009278 codes for chloroplastic Peroxiredoxin-2E-2, which is a member of the thiol peroxidase family. These enzymes play an important role in regulating Reactive Oxygen Species (ROS) by reducing hydroperoxides. Peroxiredoxin-2E-2 is present in chloroplasts, especially in reproductive tissues, and its expression is sensitive to light and salt levels(Rouhier and Jacquot, 2005).

The remaining single-copy orthogroups that are candidates for adaptive sequence evolution are involved in cell replication (OG0015603) or members of a broad gene superfamily (OG0012770).

In addition to single copy genes, we tested for adaptive sequence evolution in orthogroups with a 1:1:2 or 1:2:1 relationship, *i.e.* a single gene in *A. comosus* and a duplicated gene either in *T. leiboldiana* (1:1:2 , 108 genes), or *T. fasciculata* (1:2:1, 190). We recovered a gene family in 1:2:1 conformation coding for a protein ECIFERUM 1-like, where one of two copies in *T. fasciculata* had $\omega > 1$. In *A. thaliana,* protein ECIFERUM-1 is involved in the biosynthesis of alkanes, which form hydrophobic cuticular waxes that protect the plant from desiccation. It has been shown that changes in expression of ECIFERUM-1 affect susceptibility to water stress and pathogens, therefore linking the protein with responses to biotic and abiotic stress(Bourdenx et al., 2011).

We also recovered one candidate orthogroup in 1:1:2 conformation coding for a kinesin-like protein KIN-10C. Members of the kinesin superfamily are molecular motors playing important roles in intracellular transport of vesicles and organelles, spindle formation and elongation, chromosome segregation, morphogenesis, and signal transduction (Li et al., 2012). Both *T. leiboldiana* gene copies showed elevated dN/dS

ratios (See Table S6), though only one ratio was significant, suggesting that both gene copies have undergone significant evolution in *T. leiboldiana*.

Another candidate orthogroup in 1:2:1 conformation codes for a Ubiquitin-conjugating (UBC) enzyme E2, which plays an important role in the targeting of proteins by ubiquination for the proteasome. In mung bean, a UBC E2 enhances osmotic stress tolerance (Chung et al., 2013), and in Arabidopsis the overexpression of a soybean (Zhou et al., 2010) and peanut (Wan et al., 2011) UBC E2 protein increases drought and salt tolerance.

Note 8: Choice of reference genome, mapping bias and mapping statistics for RNA-seq analysis

Our RNA-seq dataset of 72 samples (2 species x 6 timepoints x 6 accessions) consists on average of 68,856,922 ± 15,234,149 reads per sample. Prior to conducting DGE analysis on the full set of RNA-seq reads, we tested for a potential mapping bias due to the choice of reference genome by mapping a random subset of reads consisting of 10 % of the original set of reads per sample, for a subset of 24 samples (3 samples per species, 4 time points per sample). Subsets were mapped to the *T. fasciculata*, *T. leiboldiana* and *A. comosus* (ASM154086v1) genome assemblies with STAR as described in Material and Methods. Mapping statistics were collected from STAR's final log file (Log.final.out) and visualised in R with *ggplot2* (Fig. S17 and S18).

Unique mapping rates differ the least between species when mapping to A. comosus, however, a very large proportion (> 60 %) of reads are lost (Fig S17). When mapping to *T. fasciculata* reference genome, the difference in average mapping rate between species is 2,9 %, while being 5.6 % when mapping to *T. leiboldiana*. Also the difference between species in average multimapping rate is smaller for the *T. fasciculata* reference (0.88 % versus 1.38 % for the *T. leiboldiana* reference), despite having overall higher multimapping rates (Fig. S18). Given the small difference in mapping rate differences between species when mapping to the *T. fasciculata* genome, we decided to conduct our DGE analyses on this genome primarily. Interestingly, *T. fasciculata* samples map equally well to both the *T. fasciculata* and *T. leiboldiana* genome in terms of uniquely mapping reads, while the reverse is not true (*T. leiboldiana* unique mapping rates

decrease when mapped to *T. fasciculata*). For multimapping reads, rates are typically lower when samples are mapped to their conspecific reference genome.

Mapping of the full dataset (72 samples) to the *T. fasciculata* genome resulted in an average uniquely mapping rate of 85.7 % ± 5.25 % for *T. fasciculata* samples and 85.19 % ± 0.61 % for *T. leiboldiana* samples. When mapping to the *T. leiboldiana* genome assembly, 85.4 % ± 5.25 % of *T. fasciculata* reads mapped uniquely, while *T. leiboldiana* samples had an average unique mapping rate of 93.4 % ± 1.46 %.

Note 9: Differential gene expression using the *T. leiboldiana* assembly

In addition to the DE analysis using the *T. fasciculata* genome as reference, we performed a second time-dependent DE analysis in maSigPro with the *T. leiboldiana* genome as reference to test whether the one-directional enrichment of multi-copy gene families is the result of a technical bias when using the *T. fasciculata* genome for DE analysis. It is possible that differential gene expression in additional copies of *T. leiboldiana* are missed, as these are not present in the *T. fasciculata* genome and may be too divergent to map onto a different copy.

The DE analysis on the *T. leiboldiana* reference genome recovered 863 genes with differential expression profiles in a 24-hour period between species. These belonged to 714 orthogroups, of which 458 overlapped with the set of DE orthogroups recovered in the DE analysis using *T. fasciculata* as reference genome. This is equivalent to 66 % of non-unique DE orthogroups called with *T. fasciculata* as reference and 70 % called with *T. leiboldiana* as reference. The overlap in DE orthogroups is lowest for expanded gene families in the respective genome, with only 31 % and 33 % of orthogroups larger in *T. fasciculata* and *T. leiboldiana* respectively containing DE genes in both analyses. This highlights the importance of performing timewise DE analyses on both genome assemblies.

We indeed find enrichment for gene families with gene counts higher in *T. leiboldiana*, which occur twice as often in the DE subset compared to the whole genome (Chi-square P = $1.011568e^{-33}$, Fig. 5b). We also find a small increase of multicopy families with higher gene counts in *T. fasciculata* compared to the whole genome when using

mapped reads to *T. leiboldiana* (See Results). 59 known genes related to CAM, gluconeogenesis and starch metabolism were part of the subset of DE genes detected using *T. leiboldiana* as reference genome. 49 out of the 57 orthogroups that these genes belonged to were also recovered as DE orthogroups in the DE analysis using *T. fasciculata* as reference genome. The remaining non-overlapping eight orthogroups consist of seven one-to-one orthogroups and one multi-copy orthogroup with equal counts (3:3) with one DE gene. The 14 CAM-related DE orthogroups that were only called when using *T. fasciculata* as an orthogroup consist of four one-to-one orthogroups, eight orthogroups expanded in *T. fasciculata* and two multi-copy orthogroups of equal size. Fisher's exact test on each category of DE orthogroups related to CAM, starch metabolism and gluconeogenesis found in the *T. leiboldiana* analysis shows that, unlike for all DE orthogroups, there is no significant enrichment of gene families expanded in *T. leiboldiana*. This suggests that gene family expansion in CAM-related genes is specifically found in the CAM lineage.

Note 10: On the differential expression and gene family expansion of CAM-related genes

We summarised our findings on evolutionary processes affecting CAM-related genes in figure 6, by highlighting if a gene was affected by differential gene expression, gene family expansion, high TE insertion rates or adaptive sequence evolution in or between *T. fasciculata* and/or *T. leiboldiana*. Here we give a more detailed explanation of this figure and discuss expression profiles of affected genes, which can be found in a compiled PDF here: https://github.com/cgrootcrego/Tillandsia_Genomes.

Starting with the night-time carbon fixating module of CAM, we find gene family expansion in β-carbonic anhydrase (CA) 5 in *T. fasciculata*, though no significant differential gene expression. The pineapple ortholog of this family showed diel expression and circadian regulation (Wai et al., 2017). CA converts CO_2 to HCO_3^-, which is then added to phosphoenolpyruvate (PEP) by Phosphoenolpyruvate carboxylase (PEPC). PEPC underwent an ancient duplication shared by pineapple and the two *Tillandsia* species, but only one copy (Tfas_v1.16311) shows a diel pattern in *T. fasciculata* and not in *T. leiboldiana* (Fig. 5c). The other copy is lowly expressed in both species.

An important regulator of PEPC is PEPC kinase (PPCK), which reduces PEPC's sensitivity to malate inhibition and renders it more active by phorsphorylation during the night (Nimmo et al., 2001) . PPCK (Tfasc_v1.03128-RA) shows accentuated diel expression in *T. fasciculata* compared to *T. leiboldiana* (Fig. S10). PPCK also shows an increase in night-time expression in *T. leiboldiana*, which has been reported previously in other C3 *Tillandsia* (De La Harpe et al., 2020) and in *Yucca* (Heyduk et al., 2022), suggesting a conserved time-structured expression of PPCK.

The product of PEPC's catalysed reaction is oxaloacetate, which is converted to malate by malate dehydrogenase (MDH). We detect a MDH gene family (OG0001548) with differential expression between *T. fasciculata* and *T. leiboldiana* which also shares a duplication across all three species. Similar as with PEPC, one copy shows a clear diel pattern in *T. fasciculata* while the other copy is lowly expressed (Fig. 5c). Another MDH gene family that is expanded in *T. fasciculata* (OG0002059) shows expression in both copies with a slight phase shift from *T. leiboldiana*, peaking in the late night.

The resulting malate is then transported to the vacuole, putatively by aluminium-activated malate transporters (ALMT) (Kovermann et al., 2007; Wai et al., 2017). The single-copy gene Tfasc_v1.09028-RA, which codes for aluminum-activated malate transporter 9 and is orthologous to the candidate CAM malate transporter in pineapple (Wai et al., 2017), was differentially expressed – though it shows diel expression in both *Tillandsias* and is more highly expressed in *T. leiboldiana*. This gene also shows elevated TE insertion rates in *T. fasciculata*.

Night-time malate transport into the vacuole is energised by two vacuolar pumps: vacuolar H^{+-}ATPase pump (V-ATPase) and H^{+}-PPiase (V-PPiase, AVP1), which help maintain the ionic gradient of the tonoplast (Wai et al., 2017; McRae et al., 2002). Several subunits of V-ATPase are differentially expressed, showing a variety of expression patterns, as they are spread out across four different expression clusters (cluster 2, 3, 4 and 5, Table S7), including elevated expression in *T. fasciculata* and in *T. leiboldiana*, suggesting a complex regulatory structure. Subunit A and B, which show high expression in CAM-performing tissues in pineapple, were highly expressed in *T. fasciculata* compared to *T. leiboldiana* and showed a clear diel pattern with elevated expression at night. Interestingly, a few DE subunits have undergone gene family expansion in *T.*

fasciculata (See Table S5). Subunit H (OG0000539) contains two DE gene copies and eight gene copies in total in *T. fasciculata*, as opposed to three in *T. leiboldiana*. Expression patterns of these two gene copies show elevated and diel expression in one (Tfasc_v1.19742) and reduced, non-diel expression in the other one (Tfasc_v1.24696) compared to *T. leiboldiana*. The latter gene also has an elevated TE insertion count in *T. fasciculata*, suggesting the pseudogenization of a gene that is expressed in the C3 plant, and differential co-opting of gene copies between a C3 and CAM plant. Subunit C (Tfasc_v1.17712-RA) also shows elevated TE insertion rates in both species and has an elevated, diel expression profile in *T. fasciculata*.

We also detect differential expression in a copy of AVP1, a secondary vacuolar pump, which has more gene copies in *T. fasciculata* than in *T. leiboldiana*. Two copies show diel cycling in *T. fasciculata*, peaking during the night, but one *T. leiboldiana* copy is much more highly expressed and shows a phase shift as it peaks in the late day. As found in pineapple, AVP1 is much more lowly expressed than V-ATPase subunits, however its expression profile differs from that of pineapple (peaking at midday) (Wai et al., 2017).

During daytime, malate is transported out of the vacuole and reconverted to PEP. There are two possible metabolic routes known in CAM plants, either by converting malate to oxaloacetate by MDH and decarboxylation to PEP by PEP carboxykinase (PEPCK), or by decarboxylating malate into pyruvate by malic enzyme (ME) and converting pyruvate into PEP with pyruvate orthophosphate dikinase (PPDK). In pineapple, the main carboxylase is PEPCK (Dittrich et al., 1973), and indeed we do not recover differential temporal expression in ME or PPDK, while PEPCK shows a strong diel pattern peaking during the day and dropping at night. This indicates that the daytime CAM metabolism is conserved between pineapple and *Tillandsia*.

During the day, CAM plants also accumulate transitory starch in the chloroplast and/or soluble sugars in the vacuole, which become a source of PEP at night. In Bromeliaceae, the relative importance of starch versus soluble sugars as sources of PEP is variable across species, with some species accumulating only starch, others, only soluble sugars, and others both (Christopher and Holtum, 1998). We find differentially expressed genes involved in both pathways: vacuolar transporters which bring soluble

sugars into the vacuole (SUT2 and ERD6) on the one hand, and starch synthase, α- and β-amylase and glucose-6-phosphate/phosphate translocator (GPT) for starch accumulation and phosphorylitic degradation on the other hand. Two families of vacuolar invertases, which convert sucrose to hexoses in the vacuole, show gene family expansion in *T. fasciculata* (OG0001710) and elevated TE insertion rates in *T. leiboldiana* (OG0015868). A starch synthase family that is expanded in *T. fasciculata* (OG0001599) is also differentially expressed, showing diel expression peaking during the day in *T. fasciculata* while being very lowly expressed in *T. leiboldiana* with no diel cycling.

To maintain the supply of PEP for night-time carbon fixation, vacuolar hexoses and/or transitory starch is broken down through glycolysis (Christopher and Holtum, 1996). Indeed, several glycolytic enzymes are differentially expressed and clustering together with PEPC in cluster 5, which shows diel expression peaking in the late afternoon to the early night in *T. fasciculata*: glucose-6-phosphate isomerase (Tfasc_v1.04514-RA), phosphofructokinase (Tfasc_v1.20370-RA, Tfasc_v1.24086-RA) and aldolase (Tfasc_v1.17464-RA). ATP-dependent phosphofructokinase however peaks in expression during the day, while an isoform of the expanded PPi-dependent phosphofructokinase family OG0000580 shows elevated expression in the early night. This could indicate an important role for soluble sugars stored in the vacuole as a source for night-time PEP, given that PPi-dependent phosphofructokinase cannot access fructose-6-phosphate precedent from starch accumulated in the chloroplast (Carnal and Black, 1989). A hexokinase was also called as differentially expressed in the *T. leiboldiana* genome with elevated expression in *T. fasciculata*. We also find two expanded cytosolic enolase families in *T. fasciculata* (OG0000469 and OG0000555) and one in *T. leiboldiana* (OG0001563), and an expanded glyceraldehyde-3-phosphate dehydrogenase in *T. leiboldiana* (OG0000320). The enolase family OG0000555 contains two copies with elevated night-time expression in *T. fasciculata*, while GAPDH shows one copy in each species with high expression and a phase shift between species, with expression peaking at night.

While exact regulators of CAM are not as well described as the pathway itself, we do encounter some differentially expressed circadian clock regulators which have been described previously in the context of CAM. Three DE circadian genes, GIGANTEA (GI),

REVEILLE 1 (RVE1) and LATE ELONGATED HYPOCOTYL (LHY) show a similar expression pattern with reduced diel oscillation in *T. fasciculata* compared to *T. leiboldiana*. None of these genes showed phase displacement between the two species, which differs from findings between *Agave* and *Arabidopsis* (Yin et al., 2018), particularly for RVE1. While none of these genes show gene family evolution, GI has a high number of TE insertions in both species (Table S11).

Lastly, we also recover differentially expressed regulators of stomatal movement such as ATP-binding cassette C14 (ABCC14) and succinate dehydrogenase (SDH) which exist in a multi-copy gene family. In both enzymes, one gene copy in *T. leiboldiana* shows stronger diel cycling than the copies in *T. fasciculata*. While ABC-C14 peaks during the day, SDH peaks in the late night in *T. leiboldiana*. The highest expressed copy of ABC-C14 in *T. fasciculata*, which peaks in activity in early night, also has an increased number of TE insertions.

Note 11: On the success of *de novo* assembly of highly repetitive genomes

The highly repetitive content observed in many plant genomes often causes fragmented genome assemblies. Despite considerable progress thanks to long-read sequencing technologies, little to no genomic resources are available yet for some plant clades, particularly for species with the remaining challenge of a highly repetitive content (Marks et al., 2021). The availability of long-read sequencing and chromatin conformation capture technologies have now enabled the assembly of particularly complex genomes with little fragmentation, in the best case at the chromosome-level. Our project was therefore launched and made possible because of these recent technological advances.

The kmer-based approach implemented in findGSE (k = 21, see also SI Note 1 and 2) estimates the TE content directly from raw reads, *i.e.,* prior to generating *de novo* assemblies. We estimated that the repetitive content of *T. fasciculata* and *T. leiboldiana* was around 66 % and 75 %, respectively. Based on *de novo* generated assemblies and TE annotations performed with EDTA (Ou et al., 2019), we observed remarkably consistent estimates (65.5 % and 77.1 % in *T. fasciculata* and *T. leiboldiana*, Table S3)

on the main scaffolds (SI Note 3). Such values are one of the most elevated among plant genomes assembled at chromosome scale (Pedro et al., 2021).

Additionally, our analyses of spatial distribution of TE content highlight the extreme local levels of repetitive content in these genomes, especially in *T. leiboldiana*. In non-telomeric regions, the observed repetitive content most often reaches values above 80% for *T. fasciculata* and nearly 100% for *T. leiboldiana* (Fig. 3b, Fig. S6).

Considering the extremely high repetitive content in centromeric regions, especially for *T. leiboldiana*, the limited fragmentation of our assembly can be considered as a success and therefore represent empirical evidence of the progress made in plant genomics, only twenty years after the release of the first plant genome. At the age of long-read sequencing and chromatin conformation capture technologies, the *de novo* sequencing of plant species associated with a highly repeated genomic content is becoming more and more feasible.

4. References

Ahn, C.S., Lee, J.H., Reum Hwang, A., Kim, W.T., and Pai, H.-S. (2006). Prohibitin is involved in mitochondrial biogenesis in plants. Plant J. **46**: 658–667.

Al-Qurainy, F., Gaafar, A.-R.Z., Khan, S., Nadeem, M., Alshameri, A.M., Tarroum, M., Alansi, S., Almarri, N.B., and Alfarraj, N.S. (2021). Estimation of Genome Size in the Endemic Species Reseda pentagyna and the Locally Rare Species Reseda lutea Using comparative Analyses of Flow Cytometry and K-Mer Approaches. Plants **10**.

Bolger, A.M., Lohse, M., and Usadel, B. (2014). Trimmomatic: A flexible trimmer for Illumina sequence data. Bioinformatics **30**: 2114–2120.

Bourdenx, B., Bernard, A., Domergue, F., Pascal, S., Léger, A., Roby, D., Pervent, M., Vile, D., Haslam, R.P., Napier, J.A., Lessire, R., and Joubès, J. (2011). Overexpression of Arabidopsis ECERIFERUM1 promotes wax very-long-chain alkane biosynthesis and influences plant response to biotic and abiotic stresses. Plant Physiol. **156**: 29–45.

Brown, G.K. and Gilmartin, A.J. (1989). Chromosome Numbers in Bromeliaceae. Am. J. Bot. **76**: 657–665.

Carnal, N.W. and Black, C.C. (1989). Soluble Sugars as the Carbohydrate Reserve for CAM in Pineapple Leaves : Implications for the Role of Pyrophosphate:6-Phosphofructokinase in Glycolysis. Plant Physiol. **90**: 91–100.

Choi, M. and Davidson, V.L. (2011). Cupredoxins--a study of how proteins may evolve to use metals for bioenergetic processes. Metallomics **3**: 140–151.

Christopher, J.T. and Holtum, J. (1996). Patterns of Carbon Partitioning in Leaves of Crassulacean Acid Metabolism Species during Deacidification. Plant Physiol. **112**: 393–399.

Christopher, J.T. and Holtum, J.A.M. (1998). Carbohydrate partitioning in the leaves of Bromeliaceae performing C3 photosynthesis or Crassulacean acid metabolism. Funct. Plant Biol. **25**: 371–376.

Chung, E., Cho, C.-W., So, H.-A., Kang, J.-S., Chung, Y.S., and Lee, J.-H. (2013). Overexpression of VrUBC1, a Mung Bean E2 Ubiquitin-Conjugating Enzyme, Enhances Osmotic Stress Tolerance in Arabidopsis. PLoS One **8**: e66056.

Cicconardi, F., Lewis, J.J., Martin, S.H., Reed, R.D., Danko, C.G., and Montgomery, S.H. (2021). Chromosome Fusion Affects Genetic Diversity and Evolutionary Turnover of Functional Loci but Consistently Depends on Chromosome Size. Mol. Biol. Evol. **38**: 4449–4462.

De La Harpe, M. et al. (2020). Genomic footprints of repeated evolution of CAM photosynthesis in a Neotropical species radiation. Plant Cell Environ. **43**: 2987–3001.

Delcher, A.L., Phillippy, A., Carlton, J., and Salzberg, S.L. (2002). Fast algorithms for large-scale genome alignment and comparison. Nucleic Acids Res. **30**: 2478–2483.

Dittrich, P., Campbell, W.H., and Black, C.C. (1973). Phosphoenolpyruvate carboxykinase in

plants exhibiting crassulacean Acid metabolism. Plant Physiol. **52**: 357–361.

Elliott, T.A. and Gregory, T.R. (2015). What's in a genome? The C-value enigma and the evolution of eukaryotic genome content. Philos. Trans. R. Soc. Lond. B Biol. Sci. **370**: 20140331.

Garrison, E. and Marth, G. (2012). Haplotype-based variant detection from short-read sequencing. arXiv [q-bio.GN].

Guss, J.M., Merritt, E.A., Phizackerley, R.P., and Freeman, H.C. (1996). The structure of a phytocyanin, the basic blue protein from cucumber, refined at 1.8 A resolution. J. Mol. Biol. **262**: 686–705.

Harris, R.S. (2007). Improved pairwise alignment of genomic DNA.

Hefner, T., Arend, J., Warzecha, H., Siems, K., and Stöckigt, J. (2002). Arbutin synthase, a novel member of the NRD1beta glycosyltransferase family, is a unique multifunctional enzyme converting various natural products and xenobiotics. Bioorg. Med. Chem. **10**: 1731–1741.

Heyduk, K., McAssey, E.V., and Leebens-Mack, J. (2022). Differential timing of gene expression and recruitment in independent origins of CAM in the Agavoideae (Asparagaceae). New Phytol. **235**: 2111–2126.

Kovermann, P., Meyer, S., Hörtensteiner, S., Picco, C., Scholz-Starke, J., Ravera, S., Lee, Y., and Martinoia, E. (2007). The Arabidopsis vacuolar malate channel is a member of the ALMT family. Plant J. **52**: 1169–1180.

Leroy, T., Anselmetti, Y., Tilak, M.-K., Bérard, S., Csukonyi, L., Gabrielli, M., Scornavacca, C., Milá, B., Thébaud, C., and Nabholz, B. (2021). A bird's white-eye view on avian sex chromosome evolution. Peer Community Journal **1**.

Li, J., Xu, Y., and Chong, K. (2012). The novel functions of kinesin motor proteins in plants. Protoplasma **249 Suppl 2**: S95-100.

Marks, R.A., Hotaling, S., Frandsen, P.B., and VanBuren, R. (2021). Representation and participation across 20 years of plant genome sequencing. Nat Plants **7**: 1571–1578.

McRae, S.R., Christopher, J.T., Smith, J.A.C., and Holtum, J.A.M. (2002). Sucrose transport across the vacuolar membrane of Ananas comosus. Funct. Plant Biol. **29**: 717–724.

Mérot, C., Oomen, R.A., Tigano, A., and Wellenreuther, M. (2020). A Roadmap for Understanding the Evolutionary Significance of Structural Genomic Variation. Trends Ecol. Evol. **35**: 561–572.

Nimmo, H.G., Fontaine, V., Hartwell, J., Jenkins, G.I., Nimmo, G.A., and Wilkins, M.B. (2001). PEP carboxylase kinase is a novel protein kinase controlled at the level of expression. New Phytol. **151**: 91–97.

Ou, S. et al. (2019). Benchmarking transposable element annotation methods for creation of a streamlined, comprehensive pipeline. Genome Biol. **20**: 275.

Pedro, D.L.F., Amorim, T.S., Varani, A., Guyot, R., Domingues, D.S., and Paschoal, A.R.

(2021). An Atlas of Plant Transposable Elements. F1000Res. **10**: 1194.

Pflug, J.M., Holmes, V.R., Burrus, C., Johnston, J.S., and Maddison, D.R. (2020). Measuring Genome Sizes Using Read-Depth, k-mers, and Flow Cytometry: Methodological Comparisons in Beetles (Coleoptera). G3 **10**: 3047–3060.

Rouhier, N. and Jacquot, J.-P. (2005). The plant multigenic family of thiol peroxidases. Free Radic. Biol. Med. **38**: 1413–1421.

Sun, H., Ding, J., Piednoël, M., and Schneeberger, K. (2018). findGSE: estimating genome size variation within human and Arabidopsis using k-mer frequencies. Bioinformatics **34**: 550–557.

Wai, C.M. et al. (2017). Temporal and spatial transcriptomic and microRNA dynamics of CAM photosynthesis in pineapple. Plant J. **92**: 19–30.

Wan, X., Mo, A., Liu, S., Yang, L., and Li, L. (2011). Constitutive expression of a peanut ubiquitin-conjugating enzyme gene in Arabidopsis confers improved water-stress tolerance through regulation of stress-responsive gene expression. J. Biosci. Bioeng. **111**: 478–484.

Wang, Y., Ries, A., Wu, K., Yang, A., and Crawford, N.M. (2012). The Arabidopsis Prohibitin Gene PHB3 Functions in Nitric Oxide–Mediated Responses and in Hydrogen Peroxide–Induced Nitric Oxide Accumulation. Plant Cell **22**: 249–259.

Wu, T.D., Reeder, J., Lawrence, M., Becker, G., and Brauer, M.J. (2016). GMAP and GSNAP for Genomic Sequence Alignment: Enhancements to Speed, Accuracy, and Functionality. In Statistical Genomics: Methods and Protocols, E. Mathé and S. Davis, eds (Springer New York: New York, NY), pp. 283–334.

Xiang, Y., Song, M., Wei, Z., Tong, J., Zhang, L., Xiao, L., Ma, Z., and Wang, Y. (2011). A jacalin-related lectin-like gene in wheat is a component of the plant defence system. J. Exp. Bot. **62**: 5471–5483.

Yardeni, G. et al. (2021). Taxon-specific or universal? Using target capture to study the evolutionary history of rapid radiations. Mol. Ecol. Resour.

Yin, H. et al. (2018). Diel rewiring and positive selection of ancient plant proteins enabled evolution of CAM photosynthesis in Agave. BMC Genomics **19**: 588.

Zhang, W., Peumans, W.J., Barre, A., Astoul, C.H., Rovira, P., Rougé, P., Proost, P., Truffa-Bachi, P., Jalali, A.A., and Van Damme, E.J. (2000). Isolation and characterization of a jacalin-related mannose-binding lectin from salt-stressed rice (Oryza sativa) plants. Planta **210**: 970–978.

Zhou, G.-A., Chang, R.-Z., and Qiu, L.-J. (2010). Overexpression of soybean ubiquitin-conjugating enzyme gene GmUBC2 confers enhanced drought and salt tolerance through modulating abiotic stress-responsive gene expression in Arabidopsis. Plant Mol. Biol. **72**: 357–367.
