# Supplementary material for: CAM evolution is associated with gene family expansion in an explosive bromeliad radiation
Source: Plant Cell. 2024 Apr 30;36(10):4109–31. doi: 10.1093/plcell/koae130 (PMC11449062; doi:10.1093/plcell/koae130)
Supplement: koae130_Supplementary_Data [file tpc.01080.2023-s06.pdf › tpc.01080.2023-s06.pdf]

THE PLANT CELL

CAM evolution is associated with gene family expansion in an explosive bromeliad radiation

Clara Groot Crego, Jaqueline Hess, Gil Yardeni, Marylaure de la Harpe, Clara Priemer, Francesca Beclin, Sarah Saadain, Luiz Cauz-Santos, Eva Temsch, Hanna Weiss-Schneeweiss, Michael Barfuss, Walter Till, Wolfram Weckwerth, Karolina Heyduk, Ovidiu Paun, and Thibault Leroy

Review Timeline:

Submission Date:	14-Nov-2023
Editorial Decision:	26-Dec-2023
Revision Received:	16-Feb-2024
Editorial Decision:	07-Mar-2024
Revision Received:	04-Apr-2024
Accepted:	07-Apr-2024

Dr. Clara Groot Crego
Universitat Wien
Vienna
Austria

Dear Dr. Groot Crego:

We have received reviews of your manuscript entitled "CAM evolution is associated with gene family expansion in an explosive bromeliad radiation." Thank you for submitting your best work to The Plant Cell. The editorial board agrees that the work you describe is substantive, falls within the scope of the journal, and may become acceptable for publication, pending revision and potential re-review. We ask you to pay attention to the following points in preparing your revision.

Reviewer 1 provides detailed comments on the descriptive statistics associated with the new Tillandsia genomes. In particular, R#1 notes several features in the T. fasciculata genome that could be caused by misassembly of haplotypes. Is there any evidence of co-assembly? Can this concern be addressed or resolved? Please careful consider these comments, especially in the context of subsequent analyses of gene family expansion.

Much of the comparative analyses presented rest on the identification of divergently expressed genes using a time-course RNA-seq experiment. Reviewers had questions about the experimental protocols and analysis tools utilized. Please clarify the source of plant material, sampling schemes, and basic bioinformatics pipelines used. It appears that the analysis using maSigPro centers on genes that show temporally divergent expression patterns (line 335) between T. fasciculata and T. leiboldiana. While these genes are certainly of interest, it seems this criterion would exclude any constitutively differentially expressed genes. Is this justifiable? Are there many genes with little temporal patterns but considerable divergence? A number of reviewer comments suggest additional aspects of RNA-seq analyses or visualization that may help identify important features of CAM-related divergence.

The reviewers make a number of additional suggestions that may help to improve the clarity and presentation of the manuscript. Please consider these suggestions and respond to each point-by-point in your response document.

Please contact us if there are ambiguous comments or if you wish to discuss the revision.

Given the nature of the comments, we are offering you 60 days from when we have issued this decision to complete the revision. If a revision is not returned within this time frame, and if you have not been granted an extension, we will withdraw the manuscript, which will leave you free to submit the work elsewhere. If you need an extension, we encourage you to contact us at any point before the 60 days have passed.

When you are ready to submit the revised version, please upload a highlighted copy that indicates all changes made in response to the editor and reviewer recommendations. Include an itemized list of all changes made in response to each of the reviewer's suggestions in the "Response to Reviewers" section; please note that reviewers do not have access to your cover letter, nor was this decision letter shared with them.

Thank you for the privilege of reviewing your work. We look forward to receiving your revised manuscript.

Sincerely,

The Plant Cell Board of Editors

Please note the following:

-The Plant Cell now requires authors to complete and submit an author revisions checklist upon submission of a revised manuscript. The aim of the checklist is to aid authors in preparing a high-quality manuscript, facilitate the review and assessment of revised manuscripts, and help to ensure that journal standards are maintained across the board. If your manuscript is accepted, the completed checklist will be published as supplemental material attached to the article online. Please download a copy of the checklist (pdf fillable form) at this link, for submission with your revised manuscript: https://tpc.msubmit.net/html/Author_Revisions_Checklist.pdf.

-Supplemental materials should be restricted to large datasets and tables, presentation of replicates, and validation of reagents, methods, or genotypes. Any data that are used to support the major claims must be in the main manuscript.

Supplemental figure legends must indicate what figure in the main manuscript is supported by the supplemental data presented. Please justify how each of the supplemental figures meet the criteria.

-Sampling methods and nature of "biological replicates" should be described precisely (i.e. different plants, parts of plants, pooled tissue, independent pools of tissue, sampled at different times, etc), along with a clear description of and rationale for any statistical analyses conducted. The reader should know exactly what was sampled; what forms the basis of the calculation of any means and statistical parameters reported. This is also necessary to ensure that proper statistical analysis was conducted.

-Want to add this revision deadline to your calendar? Click below!

-- Reviewer comments:

Reviewer #1 (Comments for the Author):

Crego et al. sequenced the genomes of C3 and CAM Tillandsia species and conducted detailed comparative genomics and expression analyses to identify genes underlying CAM photosynthesis. These resources are useful for the community, and there are some interesting findings from genome level analyses in these two Tillandsia species. The authors highlight the role of large-scale genome rearrangements, transposable element (TE) dynamics, and regulatory changes rather than coding sequences as important drivers for the evolution of CAM. I reviewed this manuscript previously at another journal, and I thank the authors for their detailed reflection and revision before resubmitting elsewhere. Briefly, my previous concerns related to the lack of physiology data to support CAM activity, linking CAM to structural variation without sufficient evidence, identification of CAM vs non-CAM genes, and a lack of detailed comparative analyses. Most of my concerns have been addressed in this revision, and I think the manuscript is significantly improved with stronger support for the main findings. I also appreciate the nuanced discussion of T. leiboldiana having some CAM like traits, which could indicate it is either an intermediate, or does CAM under certain conditions (such as drought). I read this paper with interest, and have a few relatively minor additional comments/concerns detailed below.

1. Based on K-mer analyses, it seems like the T. fasciculata genome is highly heterozygous, and the total assembly size is significantly larger than the estimated genome size. T. fasciculata also has a high proportion of duplicated BUSCO genes for a diploid, and relatively low unique read mapping rate for RNAseq data compared to T. leiboldiana. Is it possible more than 1 haplotype is assembled for parts of the genome? Could this factor be contributing to some of the expanded gene family results?

2. The co-expression analyses were only run on the ~900 or so differentially expressed genes between the two Tillandsia species. There are some interesting findings here, but because most genes are not included, important circadian oscillations or co-expressed genes would be missing. Could co-expression analysis be rerun with all syntenic orthologs between the two species? This would allow for more detailed downstream analyses of pathway rewiring and could explain why CAM genes are found in all 7 clusters, and not in a few clusters, like other CAM co-expression analyses have found.

3. Related, the total number of differentially expressed genes seems quite low to me given the striking morphological, physiologic, and general life history differences between these species. Were pairwise differential expression analyses run for each of the timepoints? More analyses of DE would be interesting, but probably not essential.

Reviewer #2 (Comments for the Author):

1. Overall, I think the work is well executed and that the results are novel and will be of interest to researchers within the CAM community or those studying Bromeliaceae in general. However, some specific points should be considered.
2. The authors emphasize the result that even though gene expansion has led individual genes to carry out specific functions within CAM metabolism, this is due to changes in the regulation of expression of the genes rather than functional changes at the amino acid level. This is not reflected in the manuscript title, and I think it is important to include it.
3. Inclusion of at least one other intermediate CAM/C3 species for example T. trauneri, T. punctulata or T. gymnobotrya from the dendrogram in Figure 1C, would have supported some of the conclusions reached, was this considered?
4. The authors base the determination of CAM metabolism on the fluctuations in malate content throughout a 24-hour light/dark cycle it would have been useful to include another species such as pineapple for comparison. The methodology to obtain the samples for malate and RNAseq analysis are not clear to me. Were samples extracted

individually or in batch for both methods? If I understand correctly, 6 different genotypes originally from different geographical regions were used for both species-could this have influenced the wide variation seen in Figure 2 B and C for T. fasciculata. Were they analyzed individually or in bulk? Is it possible that some T. fasciculata genotypes show weaker CAM than others? Were genotypes compared? Perhaps it would have been more consistent to carry out the RNAseq experiment with seedlings or cuttings from the plants used to obtain the genome data.
5. Synteny seems more consistent between A. comosus and T. fasciculata rather than T. fasciculata and T. leiboldiana- this seems to be interesting but is not discussed in detail.
6. If differences in expression are shown to be important why were the promoter regions of genes of interest not analyzed?
Minor points
In Figure 1 it is difficult to appreciate the differences in density of trichomes even in the magnified examples, could this be improved?
Figure S16 is not cited in the text and is a very dense figure which is not easy to interpret. Perhaps just some of the most important differences between the species could be presented and commented in detail in the text, otherwise I think the figure can be omitted.

Reviewer #3 (Comments for the Author):

This paper explores the evolution of Crassulacean Acid Metabolism (CAM) in the subgenus Tillandsia of the Bromeliaceae family, known for its rapid radiation and ecological diversification. By analyzing genome assemblies of two Tillandsia species with different photosynthetic phenotypes, the study identifies key genomic drivers behind the evolution of CAM, a water-conserving trait in plants

The methods section details the genomic analyses undertaken, including genome-wide investigations of synteny, transposable element (TE) dynamics, sequence evolution, gene family evolution, and temporal differential expression. The techniques are well-described and appropriate for the study's objectives.

The results indicate that large-scale genomic rearrangements and a dynamic TE landscape have significantly shaped the genomes of Tillandsia. Interestingly, the study finds that regulatory evolution, rather than coding sequence evolution, is primarily responsible for the rewiring of photosynthetic metabolism in these species.

Additionally, gene orthology analyses reveal accelerated expansion in CAM-related gene families in the constitutive CAM species, underscoring the role of gene family evolution in CAM development.

The authors effectively argue that regulatory changes, rather than changes in gene sequences, play a pivotal role in adapting photosynthetic pathways. The implications of these findings for understanding plant adaptation and ecological diversification are well-articulated.

Overall, this manuscript seems good quality.

Revisions
The authors report the identification of 34,886 and 38,180 gene models in Tillandsia fasciculata and T. leiboldiana, respectively. Significantly, 6,261 genes in T. fasciculata and 4,693 in T. leiboldiana were classified into 273 non-unique gene families, a count notably higher than that found in Ananas comosus (Ming et al., 2015).

Additionally, their analysis of orthologue gene families shows that in A. comosus, T. fasciculata, and T. leiboldiana, 21,045 (78%), 26,325 (87.5%), and 23,584 (75%) gene models were assigned to orthogroups, respectively. Of these, 10,021 are single-copy orthologs across all three species (as shown in Table S4). The authors observe that T. leiboldiana possesses fewer genes than T. fasciculata, leading to some discrepancies in the results. The manuscript could benefit from a clearer explanation of this observation in the discussion section. The authors might also consider adding a syntenic analysis between T. fasciculata and T. leiboldiana to investigate the reasons behind the differences in gene count. For this comparison, the reviewer suggest that using the 'anchor' file from McScan (Python version) with --full option could be more informative than GENESPACE, because they will show the missing genes for each species. The result might need to be manipulating.

Figure S4 showed interesting approach. Reviewer would like to ask the synteny analysis in the break point with McScan micro synteny comparion visualization. It will show clear picture coupled with author's analysis.

Reviewer expresses an interest about TE contents in breakpoint.

The review raises a question about whether the difference in gene count affects the number of transcription factors and

the size of transcription factor families.

The reviewer expresses an interest in number of tandem duplications in both T. fasciculata and T. leiboldiana and infulence to CAM related genes in figure 6.

The authors mention GDPDL-7 and GLR, whether there is significant dn/ds value in CAM pathway genes in Figure 6.

The reviewer requests three way dn/ds value comparison in CAM pathway genes among A. comosus,Kalanchoe, T. fasciculata, and T. leiboldiana for each CAM related gene orthologs. Please check the supp. table in Kalanchoe manuscript.

Regarding Figure 3b, it is unclear whether A. comosus has been reanalyzed with EDTA, and this needs clarification. The A. comosus genome from CoGe or Phytozome repeat might be based of RepeatMasker analysis.

The reviewer requests more detailed information in Figure 6 regarding the pathway of Crassulacean Acid Metabolism (CAM), particularly focusing on genes detected as differentially expressed, with gene family expansion compare to Kalanchoe (outgroup) and A. comosus, signs of adaptive sequence evolution, or elevated TE insertion counts.

Figure S7, CPM is not a recommendable choice to show the expression dynamic in CAM and other circadian genes. And this 6 point time course can get a benefit from DESEQ2 with Likelihood Ratio Test instead of MaSigPro.

The reviewer asked the temporal expression pattern from plot or heatmap (zscore normalize by gene) regarding CAM related genes in figure 6.

Concised verion of 'Note 14: Limitations of this study and future directions' might need to be added to main text discussion part.

The reviewer asks for clarification in Table 1 about TE insertion and suggests including information on TE families, such as helitrons, especially in the context of their role in expression control in Brassica. In addition, the insertion with 3kbp upstream might be more interesting.

Finally, reviewer request in-depth discussion related to CAM pathway genes.

Some minors are below.

For Figure 2, the reviewer suggests modifying the shape of the PCA plot to better distinguish between T. leiboldiana and T. fasciculata.

The reviewer requests a higher resolution for Figure S13. Cannot read estimated genome size.

The reviewer also reminds the authors to italicize all scientific names in the manuscript.

The reviewer notes that the file 'Tfas_Tlei_rearrangements.pdf' mentioned in the manuscript is missing from the provided GitHub repository.

Responses to the Editor:

1. Reviewer 1 provides detailed comments on the descriptive statistics associated with the new Tillandsia genomes. In particular, R#1 notes several features in the T. fasciculata genome that could be caused by misassembly of haplotypes. Is there any evidence of co-assembly? Can this concern be addressed or resolved? Please careful consider these comments, especially in the context of subsequent analyses of gene family expansion.

We thank the editor and reviewer for this comment. A more detailed discussion can be found in the response to reviewer 1's comment, but here follows a brief summary. We were indeed aware of the potential issues that *T. fasciculata*'s relatively high heterozygosity could cause to our analyses. Still, we want to note that based on other estimates of diversity in this species, *T. fasciculata* doesn't exhibit diversity rates beyond what is commonly found among plants and animals. Nonetheless, we inserted multiple steps in our pipelines to account for this as much as possible. We applied strict correction rates during primary assembly and ran the program PurgeHaplotigs on the *T. fasciculata* genome to collapse contigs regarded as co-assembled haplotypes. This already greatly reduced the number of input contigs for scaffolding with Hi-C. Additionally, we corrected the orthogroup sizes based on the average per-gene coverage of each defined gene model. Whenever the average coverage of a set of genes within an orthogroup deviated from the expected coverage based on single-copy gene models, the size was corrected to the number of genes in the family that are expected to be present based on their summed coverage. This step also greatly reduced the number of multi-copy families and their per-family gene counts. We also show that size correction did not greatly affect the multi-copy gene families that were considered DE and CAM-related, meaning that their copy numbers are supported by coverage and unlikely to be an inflation stemming from haplotype co-assembly.

2. Much of the comparative analyses presented rest on the identification of divergently expressed genes using a time-course RNA-seq experiment. Reviewers had questions about the experimental protocols and analysis tools utilized. Please clarify the source of plant material, sampling schemes, and basic bioinformatics pipelines used. It appears that the analysis using maSigPro centers on genes that show temporally divergent expression patterns (line 335) between T. fasciculata and T. leiboldiana. While these genes are certainly of interest, it seems this criterion would exclude any constitutively differentially expressed genes. Is this justifiable? Are there many genes with little temporal patterns but considerable divergence? A number of reviewer comments suggest additional aspects of RNA-seq analyses or visualization that may help identify important features of CAM-related divergence.

Given that our analysis compares gene expression between two species, we expect many genes to have evolved differential expression due to genetic drift occurring independently in each lineage, or due to differential ecological adaptation. However, it is challenging to discern what expression changes are due to drift, due to adaptation in other traits, or due to adaptation of photosynthetic metabolism (the trait of interest in this study) when

considering constitutive changes. We decided to therefore apply an additional criterion: not only must the genes be differentially expressed between the two species, but they must also show a differential expression curve across a diel cycle. This is specifically because CAM photosynthesis utilizes existing C3 enzymes but alters the timing at which these enzymes are active. We therefore assume that diel expression curves of genes that significantly differ between the two species are likely to underlie the diverged photosynthetic metabolisms. While it is true that this is a conservative approach, it is a way of avoiding the noise in our analyses that would be caused by genes that have diverged due to drift or other adaptive traits which were not the focus of this study. It also provides us with an additional line of evidence to treat these genes as candidates for CAM photosynthesis - confirmed by the fact that most core CAM genes (Fig. 6) are found in this set of DE genes. A more in-depth discussion of this point can be found in the response to the reviewers, in which we include an example of DE analysis considering constitutive changes between species, leading to a much noisier but less precise analysis, as multiple known CAM-related genes that were considered DE with our original approach were not found with the constitutive approach.

Reponses to the Reviewers

Reviewer #1 (Comments for the Author):

Crego et al. sequenced the genomes of C3 and CAM Tillandsia species and conducted detailed comparative genomics and expression analyses to identify genes underlying CAM photosynthesis. These resources are useful for the community, and there are some interesting findings from genome level analyses in these two Tillandsia species. The authors highlight the role of large-scale genome rearrangements, transposable element (TE) dynamics, and regulatory changes rather than coding sequences as important drivers for the evolution of CAM. I reviewed this manuscript previously at another journal, and I thank the authors for their detailed reflection and revision before resubmitting elsewhere. Briefly, my previous concerns related to the lack of physiology data to support CAM activity, linking CAM to structural variation without sufficient evidence, identification of CAM vs non-CAM genes, and a lack of detailed comparative analyses. Most of my concerns have been addressed in this revision, and I think the manuscript is significantly improved with stronger support for the main findings. I also appreciate the nuanced discussion of T. leiboldiana having some CAM like traits, which could indicate it is either an intermediate, or does CAM under certain conditions (such as drought). I read this paper with interest, and have a few relatively minor additional comments/concerns detailed below.

We thank the reviewer for taking their valuable time to review this manuscript twice, and for their constructive and helpful feedback in both rounds of review.

1. Based on K-mer analyses, it seems like the T. fasciculata genome is highly heterozygous, and the total assembly size is significantly larger than the estimated genome size. T. fasciculata also has a high proportion of duplicated BUSCO genes for a diploid, and relatively low unique read mapping rate for RNAseq data compared to T. leiboldiana. Is it possible more than 1 haplotype is assembled for parts of the genome? Could this factor be contributing to some of the expanded gene family results?

We thank the reviewer for bringing up this point and agree that this is an important aspect to consider in our analyses. We first want to note that the levels of nucleotide diversity as estimated with the Kmer strategy (*i.e.* Fig. S17) only represent raw estimates that should be consolidated. We estimated the levels of genetic diversity of a few *Tillandsia* species, including the focal species of this study, in a recent paper (Yardeni et al. 2021). By considering the diversity at synonymous sites of a large set of genes, we estimated an average π_S of 5.7 x 10^{-3} and 8.1 x 10^{-3} for *T. leiboldiana* and *T. fasciculata*, respectively. Despite the fact that we could anticipate that the π_S estimates slightly underestimate the genomic π due to the effect of linked selection, it should be noted that such levels of diversity are relatively common in the animal and plant kingdoms (e.g. Plomion et al. 2018; Buffalo 2021).

Nonetheless, we want to point out the steps taken to minimize the effect of heterozygosity in *T. fasciculata* on downstream analyses. During genome assembly with CANU, we applied an increased error correction rate (correctedErrorRate=0.105) and performed two rounds of correction in *T. fasciculata*. Afterwards, the pipeline PurgeHaplotigs was run for this species specifically to mitigate this potential issue. This resulted in the collapse of 7,877 contigs out of 16,247 initially assembled by CANU, significantly reducing the number of haplotigs present in the assembly.

After assembly, we performed further steps to account for this potential issue. We calculated the average per-gene coverage in all gene models to detect whether we still had candidate gene families with inflated sizes. We display the distribution of per-gene average coverage for different categories of genes based on the type of orthogroup (single-copy, multi-copy, etc.) in figure S12 (fig. S15 in previous version). The figure shows an uptick of intermediate coverage values in expanded families in *T. fasciculata* and in multi-copy gene families uniquely called in *T. fasciculata*. SI Note 6 described in detail how we corrected gene family sizes using coverage values (note: this is now included in the main text in the last version). Briefly, we calculated the sum of per-gene average coverage across each orthogroup and species. We then divided this sum by the expected per-gene coverage, which we defined based on the average single-copy gene coverage across all single-copy orthologs (with a homolog in other species). This resulted in a corrected gene count more accurately reflecting the true number of gene copies in each species and orthogroup. These corrected gene sizes were used for all downstream analyses (including enrichment of multi-copy gene families in gene families with DE genes) and are also reported in the manuscript (Main text and Table S4). We attach a small schematic drawing of the entire pipeline for clarity.

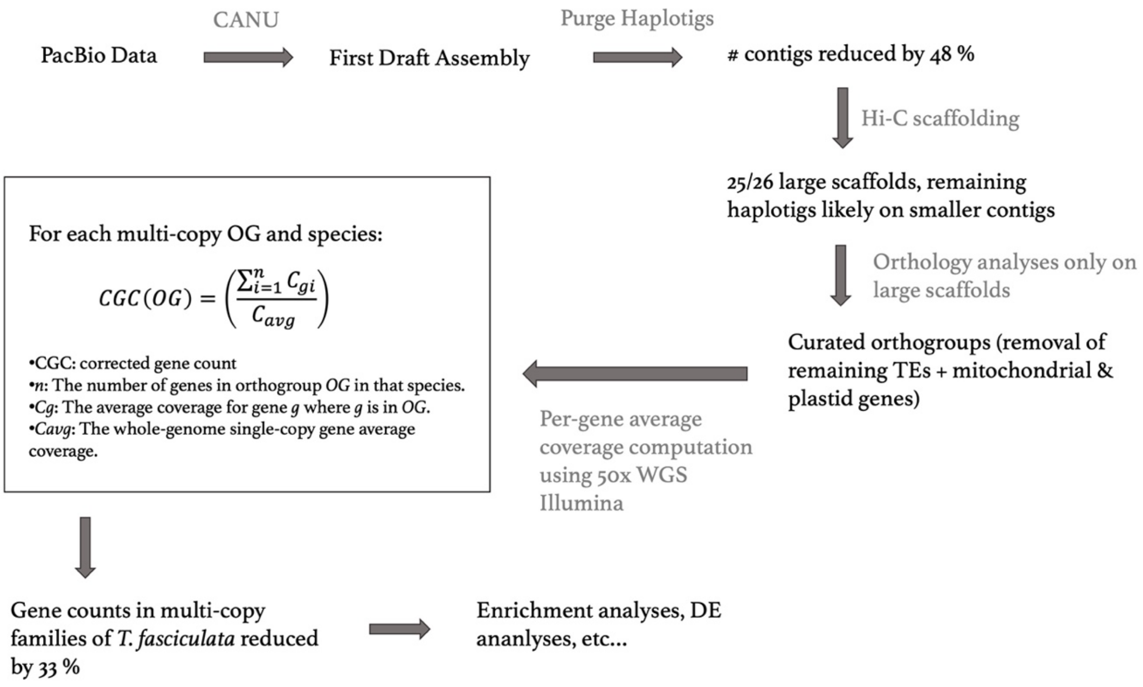

We also want to note that gene families called uniquely in one species (no orthology with any other species) were not considered in any downstream analysis, as we considered them likely unreliable annotations and preferred to stay conservative in the study of gene family sizes. Similarly, any genes considered plastid or mitochondrial were also removed from these analyses, due to the difficulty of reliably correcting gene family size using coverage. Originally, 9,210 genes in *T. fasciculata* and 6,257 genes in *T. leiboldiana* were assigned to orthogroups with multiple gene copies in at least one species. After correcting orthogroup sizes by coverage, we retained 6,261 and 4,693 gene counts in total (Table S4).

As our analyses are limited to genes located on the larger, contiguous scaffolds in both assemblies, we expect a higher likelihood of haplotype co-assembly within adjacent copies than among copies which are distant or on different contigs of the assembly. As explained in the response to comment 5 of Reviewer 3, there are only few orthogroups included in DE families with copies adjacent to each other, and none among orthogroups with CAM-related genes. Indeed, of the 10 expanded CAM-related orthogroups, only two groups had their gene counts reduced after correction, pointing at the robustness of the estimated family sizes. We also want to point out that the signature of multi-copy enrichment in DE gene families was not limited to expanded gene families in *T. fasciculata*, but was also significant in ancient gene duplications shared between *T. leiboldiana* and *T. fasciculata*, something that most likely wouldn't be affected by co-assembly of haplotypes in independent assemblies.

We therefore argue that our assembly strategy and downstream analyses account and correct for potential existence of co-assembly of haplotype and mitigate this issue significantly. While we cannot guarantee complete removal of haplotype co-assembly in the genomes, we have checked and corrected the final set of CAM-related expanded gene families to the best of our ability.

For better clarity of this part of our methodology, we have transferred the entirety of SI Note 6 to the Methods section.

2. The co-expression analyses were only run on the ~900 or so differentially expressed genes between the two Tillandsia species. There are some interesting findings here, but because most genes are not included, important circadian oscillations or co-expressed genes would be missing. Could co-expression analysis be rerun with all syntenic orthologs between the two species? This would allow for more detailed downstream analyses of pathway rewiring and could explain why CAM genes are found in all 7 clusters, and not in a few clusters, like other CAM co-expression analyses have found.

We thank the reviewer for this comment. Our idea behind our fine-grained co-expression analyses was to illustrate in detail the diversity of expression behavior among time-wise DE genes, rather than to further broaden the range of genes that are potential candidates underlying CAM evolution.

We like to stress that our experiment was specifically designed to depict temporally different patterns between species with different CAM/C3 phenotypes, and hence is expected to indicate most genes with a temporally distinct pattern. If a co-expression analysis is run on all genes of a species, regardless of whether they are DE or not, we would expect a higher number of modules of which many would consist of non-cycling and constitutively expressed genes. In that context, CAM genes would indeed be restricted to a subset of co-expression modules, but we expect the information value of such coarse analyses will be lower.

A co-expression analysis on all genes will forcefully lump genes with similar expression profiles into the same module, but we have little evidence that these genes are for that reason linked to CAM evolution - it could be by chance that they show similar expression profiles, or they could be linked to other day-night fluctuating activities. On the other hand, the DE genes we study in the manuscript show significant differential time-wise expression between two species that show divergence in photosynthetic metabolism, giving an extra line of evidence that they may be related to CAM. In fact, we find numerous genes previously described as CAM-related or part of pathways that are linked to CAM with this approach, including circadian clock regulators.

We therefore argue that while we agree that our approach may be conservative by limiting our study of expression behaviour to those genes that behave differently across time between both species, we consider that we keep a focused approach on our aim and avoid

inflating our findings by including genes that show correlated but constitutive expression profiles between the species.

3. Related, the total number of differentially expressed genes seems quite low to me given the striking morphological, physiologic, and general life history differences between these species. Were pairwise differential expression analyses run for each of the timepoints? More analyses of DE would be interesting, but probably not essential.

We agree with the reviewer that our RNA-seq dataset contains potential for additional analyses. Given the large array of analyses already incorporated in this manuscript, and our particular focus on CAM photosynthesis, we restricted our DE analysis to finding genes that can be considered likely related to CAM photosynthesis because their expression profile has been rewired within a diel cycle - given that CAM restructures the photosynthetic process throughout the daily cycle. The added requirement for a gene not only to be generally DE between species, but also DE across a diel cycle, greatly reduces the number of called DE genes. This is to our advantage, since *T. fasciculata* and *T. leiboldiana* are related, but not sister species. At this level of divergence, we expect them to have accumulated many differences in expression due to their independent evolutionary history and drift. For that reason, we expect that studying constitutive expression changes will result in much larger numbers of called genes, underlying processes not necessarily related to the specific ecological differentiation related to photosynthetic metabolism.

To explore this, we have performed pairwise DE analyses in DESEQ2 of each timepoint, calling DE genes between species with a p-adj value < 0.01 and a log-fold change of 2. As expected, this resulted in much larger numbers of DE genes, between 1,737 DE genes at N+9 and 2,304 genes at D+9. Many of the DE genes called in this approach are DE between species at all timepoints (853 genes, 21 %) and therefore represent constitutive expression changes that occurred during the divergence of the two species. GO term enrichment of each timepoint results in 403 enriched GO terms at the "Biological Process" level across all timepoints, compared to 226 terms across clusters called by maSigPro. Nonetheless, terms that were enriched in the maSigPro set that are related to CAM are missing in the larger set from DESEQ2, such as "vacuolar acidification" and "phosphoenolpyruvate carboxylase activity".

MaSigPro's approach results in a less noisy dataset (fewer genes) yet a more sensitive approach (we recover many key CAM genes). Therefore, for the purposes of our study, we believe that our existing approach is more appropriate than a pairwise approach - however, future studies with different questions or traits of interest will be able to use this dataset as a resource.

Reviewer #2 (Comments for the Author):

1. Overall, I think the work is well executed and that the results are novel and will be of interest to researchers within the CAM community or those studying Bromeliaceae in general. However, some specific points should be considered.

We thank the reviewer for the supportive comment and constructive feedback below.

2. The authors emphasize the result that even though gene expansion has led individual genes to carry out specific functions within CAM metabolism, this is due to changes in the regulation of expression of the genes rather than functional changes at the amino acid level. This is not reflected in the manuscript title, and I think it is important to include it.

We thank the reviewer for the suggestion. While amino acid changes indeed do not seem a major driver of divergence in the photosynthetic metabolism of these two species, we don't exclude these changes to possibly occur and become established at later stages of divergence, as written in the discussion. Given that the dN/dS test is a relatively stringent test of selection requiring a fairly large number of substitutions, we believe that additional work using population data should ascertain this finding further. Therefore, we feel that including it in our title would be a too definite way of communicating this finding.

3. Inclusion of at least one other intermediate CAM/C3 species for example T. trauneri, T. punctulata or T. gymnobotrya from the dendrogram in Figure 1C, would have supported some of the conclusions reached, was this considered?

Given budget limitations and other constraints, the decision was made to generate genome assemblies of two species, and the species that represented the extreme forms of the CAM/C3 phenotype based on information available at that time (carbon isotope measurements) were picked.

4. The authors base the determination of CAM metabolism on the fluctuations in malate content throughout a 24-hour light/dark cycle it would have been useful to include another species such as pineapple for comparison. The methodology to obtain the samples for malate and RNAseq analysis are not clear to me. Were samples extracted individually or in batch for both methods? If I understand correctly, 6 different genotypes originally from different geographical regions were used for both species-could this have influenced the wide variation seen in Figure 2 B and C for T. fasciculata. Were they analyzed individually or in bulk? Is it possible that some T. fasciculata genotypes show weaker CAM than others? Were genotypes compared? Perhaps it would have been more consistent to carry out the RNAseq experiment with seedlings or cuttings from the plants used to obtain the genome data.

Samples for RNA-seq and metabolomics were obtained from the same leaf cuttings that were snap frozen and stored in a -80°C freezer. For each plant, an entire leaf was sampled at each time point. We therefore have 72 leaf cuttings, which were used both for obtaining RNA-seq data and for the metabolomic analyses. In both procedures, samples were extracted in randomized batches to avoid batch effects in the analyses. The metabolomics

extraction was performed in three randomized batches, while RNA extractions were done in randomized groups of 4-6 samples at a time. This information has now been explicitly stated in the Methods section.

The reviewer is right in pointing out the diverse origins of the used genotypes in relation to the observed variance both at the metabolomic and expression level. We believe that including genotypes across the distribution range of both species will result in a better representation of the species than using clonal accessions. Indeed, clonal accessions of the genome assembly accessions would reduce the observed variance in the analyses but introduce noise in the significance determination and make it more difficult to draw conclusions on a broader species level, and especially to make comparisons between two species.

Like with any other complex trait, variation within a population or species is expected, especially in cases like *T. fasciculata,* which is a widely distributed and genetically diverse species. However, we believe that the summarizing statistics that we employ both in the metabolomic and RNA-seq analyses account for within group variation and still clearly show, despite the existing variation, distinct phenotypes between species.

As for using pineapple in the phenotypic analyses - our study was conceived with the key idea to study CAM evolution between closely related species to avoid inflation of the observed changes needed to obtain CAM (see Heyduk et al. 2019). Pineapple being approximately 15 my diverged from *Tillandsia*, it may represent an independent evolutionary path to CAM evolution. At the same time, the phenotypic and genetic basis of CAM in pineapple has already been well described in other studies (Ching M. Wai et al. 2017; Ching Man Wai and VanBuren 2018; J. Chen, Zeng, and Zhang 2021; Ming et al. 2015), so we are not certain that including pineapple in our metabolomic analyses would have benefitted the study and the community significantly.

5. Synteny seems more consistent between A. comosus and T. fasciculata rather than T. fasciculata and T. leiboldiana-this seems to be interesting but is not discussed in detail.

Thank you for this comment. We agree that we didn't give enough attention to this finding. We have added a few sentences (lines 262-265) to the main text to point this out more clearly.

6. If differences in expression are shown to be important why were the promoter regions of genes of interest not analyzed?

We understand the motivation of the reviewer's comment and have included a new analysis to identify 4 known circadian promoter motifs in the 2-kb upstream region of DE genes. We have compared the per-kb frequency of each of these motifs in DE genes with the frequency in non-DE genes, loosely following the approach of (L.-Y. Chen et al. 2020), to see if these motifs are enriched in DE genes in both species. Additionally, for a set of

core CAM enzymes (PEPC, PPCK, MDH, etc.) we have characterized the motifs for all gene copies in their respective orthogroups (DE and non-DE copies). This has resulted in interesting findings which emphasize the circadian aspect of CAM evolution and support some of the findings already discussed in the manuscript. The Methods, Results and Discussion sections have been amended to include these analyses.

Minor points
In Figure 1 it is difficult to appreciate the differences in density of trichomes even in the magnified examples, could this be improved?

Thank you for this comment. We have taken new photos using a microscope that give a much better idea of the difference in trichome density.

Figure S16 is not cited in the text and is a very dense figure which is not easy to interpret. Perhaps just some of the most important differences between the species could be presented and commented in detail in the text, otherwise I think the figure can be omitted.

We apologize for the oversight - The figure is discussed in SI note 12 but was not referred to properly. We have included a small section in the results section (lines 416-424) and given a few additional descriptive statistics of the distribution of DE genes across the genome. SI Note 12 has been removed, since the information is now in the main text. We opted to maintain figure S16, as it helps visualize the differences between species in the genomic distribution of DE genes.

Reviewer #3 (Comments for the Author):

This paper explores the evolution of Crassulacean Acid Metabolism (CAM) in the subgenus Tillandsia of the Bromeliaceae family, known for its rapid radiation and ecological diversification. By analyzing genome assemblies of two Tillandsia species with different photosynthetic phenotypes, the study identifies key genomic drivers behind the evolution of CAM, a water-conserving trait in plants

The methods section details the genomic analyses undertaken, including genome-wide investigations of synteny, transposable element (TE) dynamics, sequence evolution, gene family evolution, and temporal differential expression. The techniques are well-described and appropriate for the study's objectives.

The results indicate that large-scale genomic rearrangements and a dynamic TE landscape have significantly shaped the genomes of Tillandsia. Interestingly, the study finds that regulatory evolution, rather than coding sequence evolution, is primarily responsible for the rewiring of photosynthetic metabolism in these species.

Additionally, gene orthology analyses reveal accelerated expansion in CAM-related gene families in the constitutive CAM species, underscoring the role of gene family evolution in CAM development.

The authors effectively argue that regulatory changes, rather than changes in gene sequences, play a pivotal role in adapting photosynthetic pathways. The implications of these findings for understanding plant adaptation and ecological diversification are well-articulated.

Overall, this manuscript seems good quality.

We thank the reviewer for their time to review this manuscript and for their helpful and constructive feedback.

Revisions
1. The authors report the identification of 34,886 and 38,180 gene models in Tillandsia fasciculata and T. leiboldiana, respectively. Significantly, 6,261 genes in T. fasciculata and 4,693 in T. leiboldiana were classified into 273 non-unique gene families, a count notably higher than that found in Ananas comosus (Ming et al., 2015).

Additionally, their analysis of orthologue gene families shows that in A. comosus, T. fasciculata, and T. leiboldiana, 21,045 (78%), 26,325 (87.5%), and 23,584 (75%) gene models were assigned to orthogroups, respectively. Of these, 10,021 are single-copy orthologs across all three species (as shown in Table S4). The authors observe that T. leiboldiana possesses fewer genes than T. fasciculata, leading to some discrepancies in the results. The manuscript could benefit from a clearer explanation of this observation in the discussion section. The authors might also consider adding a syntenic analysis between T. fasciculata and T. leiboldiana to investigate the reasons behind the differences in gene count. For this comparison, the reviewer suggest that using the 'anchor' file from McScan (Python version) with --full option could be more informative than GENESPACE, because they will show the missing genes for each species. The result might need to be manipulating.

While we were unable to find the –full option in the currently available release of McScan (https://github.com/tanghaibao/jcvi/wiki/MCscan-(Python-version) we investigated the possibility of genes missing in our annotation with a different approach. Using tblastn, we blasted protein sequences from orphan genes (orthogroups with orthology 0:1 and 1:0 between *T. fasciculata* and *T. leiboldiana*) to the assembly of the other species, where the homologous gene is missing. We then filtered hits that we considered reliable (best hit e-value < 1e-5) and chose chromosome triplets that seemed highly syntenic based on our GENESPACE analyses, to count the number of orphan genes with reliable hits specifically on the homologous chromosome in the other species. We considered the three triplets already discussed in the manuscript (Fig. 3b, Fig. S2) and three additional triplets, to ensure that our conclusions are robust.

Below a table with results for 6 syntenic chromosome pairs:

Chr Triplets	Triplet shown in:	Focal species	chr focal species	# orphan genes on chr	# genes with hit eval<1e-5	% genes with reliable hits	# genes w best hit on homolog chr + identity>=75%	% genes w best hit on homolog chr + identity>=75%
Acom2/Tfas4/Tlei1	Fig. 3B	Tfas	4	122	16	13.11	7	5.74
		Tlei	1	121	13	10.74	8	6.61
Acom6/Tfas11/Tlei15	Fig. S2	Tfas	11	109	16	14.68	4	3.67
		Tlei	15	120	13	10.83	7	5.83
Acom11/Tfas12/Tlei5	Fig. S2	Tfas	12	66	8	12.12	5	7.58
		Tlei	5	117	10	8.55	4	3.42
Acom5/Tfas1/Tlei2	additional	Tfas	1	117	23	19.66	6	5.13
		Tlei	2	150	22	14.67	6	4.00
Acom9/Tfas5/Tlei10	additional	Tfas	5	82	16	19.51	7	8.54
		Tlei	10	117	16	13.68	8	6.84
Acom10/Tfas8/Tlei8	additional	Tfas	8	85	15	17.65	7	8.24
		Tlei	8	149	11	7.38	4	2.68
**Total/Average 6 triplets**		**Tfas**	**1,4,5,8,11,12**	**581**	**94**	**16.18**	**36**	**6.20**
		**Tlei**	**1,2,5,8,10,15**	**774**	**85**	**10.98**	**37**	**4.78**

A low proportion of genes exhibit reliable best hits in homologous chromosomes, in such a way that the genes are probably truly absent in the other assembly. For those few genes exhibiting reliable hits, it is important to note that homology alone is not sufficient to predict a gene, and that softwares like MAKER and BRAKER consider additional criteria to predict a gene. Even if we assume that these "missing" genes are true, their number is very limited and their occurrence is as rare in one assembly as the other. Therefore, we don't think that missing genes in *T. leiboldiana* explain the discrepancy in gene counts between species. These results emphasize the robustness of the gene model prediction and orthology analysis.

More broadly, we would like to place greater emphasis on table S4, which shows that the largest difference in gene counts between *T. fasciculata* and *T. leiboldiana* can be found in multi-copy orthogroups which are shared between the two species, but where the family is larger in *T. fasciculata* than in *T. leiboldiana*. Because of high heterozygosity values in *T. fasciculata*, we applied a coverage-based approach to correct gene family sizes (see also the response to the first comment of Reviewer 1). Even after this correction, gene family sizes of multi-copy genes are larger in *T. fasciculata* than in *T. leiboldiana*. This finding corresponds to the gene family evolution analysis of De La Harpe et al. (2020), where rates of gene duplication and loss were generally elevated in the subgenus *Tillandsia* compared to other related clades. More specifically, duplication rates were higher in *T. fasciculata* than in *T. leiboldiana*, while loss rates were comparatively higher in *T. leiboldiana* than in *T. fasciculata*. Our results presented here and in the manuscript align with the existing literature and indicate that the higher gene count in *T. fasciculata* is related to a net increased gene duplication rate in this branch, and not due to technically missing gene copies in *T. leiboldiana*.

The reviewer points out that our orthology analysis finds a larger number of non-unique orthogroups than in Ming et al. 2015. We believe this is because orthology in Ming et al. 2015 was tested between more distantly related taxa than in our study. While Ming et al. ran orthology of pineapple with genomes outside Bromeliaceae, we limited our orthology to bromeliad species. This likely explains the difference, since the shorter divergence times between the species used in our study makes it more likely to identify homologs.

We have added a few sentences in the discussion (lines 601-604) to clarify this finding.

2. Figure S4 showed interesting approach. Reviewer would like to ask the synteny analysis in the break point with McScan micro synteny comparion visualization. It will show clear picture coupled with author's analysis.

We considered a more fine-grained analysis, but in most cases our defined breakpoint regions are too imprecise to allow for more detailed analyses with the data at hand. We stress that we strive for a balance between the level of uncertainty and gain from a particular analysis. Given that we find little association between DE or CAM-related genes and rearrangements, with no clear signal of CAM genes being around breakpoint regions, we have reservations about the contribution of a microsynteny analysis to our study's core objectives, as it may introduce imprecision in our results without a significant contribution to the study's goals.

3. Reviewer expresses an interest about TE contents in breakpoint.

Below a table reporting the percentage of masked bases in the breakpoints that we were able to define based on LastZ alignments:

scaffold	species	start	end	length	type	% masked
scf14	Tlei	28588630	28625508	36878	FUSION	46.81 %
scf13	Tfas	1142870	1286057	143187	TRANSLOCATION1	32.99 %
scf13	Tfas	4146882	4155429	8547	TRANSLOCATION1	75.11 %
scf19	Tlei	2748905	2760927	12022	TRANSLOCATION1	77.91 %
scf3	Tlei	15668132	16509731	841599	TRANSLOCATION1	95.96 %
scf10	Tfas	22956800	22963481	6681	TRANSLOCATION2	25.45 %
scf13	Tlei	37784284	37870718	86434	TRANSLOCATION2	85.46 %
scf23	Tlei	3341483	3382884	41402	TRANSLOCATION2	78.96 %

Note that the precision with which each breakpoint is defined varies greatly, and that further study is necessary to fully ascertain these rearrangements and the location of the breakpoint. The length of the region in which we believe a breakpoint may lie varies strongly, ranging from a span of 6.6 kb (scf10 in *T. fasciculata*) to 842 kb (scf3 in *T. leiboldiana*), which also influences the calculated TE contents.

The TE content inside candidate breakpoint regions is very high in *T. leiboldiana* except for the fused scaffold scf14 (see Table above). TE contents are much lower in breakpoints defined in *T. fasciculata*. Given *T. fasciculata*'s more conserved synteny and karyotype, it may be that the increased TE content in *T. leiboldiana* played a role in the rearrangement of chromosomes, however we think that a more detailed study which is not within the scope of this work should investigate this in more depth.

4. The review raises a question about whether the difference in gene count affects the number of transcription factors and the size of transcription factor families.

We did not investigate this previously, but we provide some results here. We selected all orthogroups containing genes with GO-term GO:0003700 (DNA-binding transcription factor activity) and considered this as a raw list of transcription factor families. The list contains 528 orthogroups in total, of which 403 (76 %) are single-copy in both species, 53 (10 %) are expanded in *T. fasciculata*, 18 (3 %) are expanded in *T. leiboldiana*, 7 (1 %) have equal numbers of genes in both species, and 18 (3 %) and 29 (5 %) are unique to *T. fasciculata* and *T. leiboldiana* respectively. Compared to the whole set of orthogroups, these orthogroups are enriched for family expansion in *T. fasciculata* (Fischer's p-value = 5x10-8) and unique families in *T. fasciculata* (6.7x10-3), while being slightly depleted for multi-copy families with equal size in both species (p-value = 0.03).

5. The reviewer expresses an interest in number of tandem duplications in both T. fasciculata and T. leiboldiana and infulence to CAM related genes in figure 6.

We give here a brief overview of tandem duplications in DE orthogroups. We qualified an orthogroup as containing a tandem duplication when two paralogs are adjacent to each other on a scaffold at a maximum distance of 5 kb, with no other genic features between them. In *T. fasciculata*, we discovered 38 DE orthogroups that contained tandem duplications, which is only 5.5 % of all DE orthogroups. Of the multi-copy orthogroups that are expanded in *T. fasciculata*, 27 (22 %) contained a tandem duplication. However, none of the DE orthogroups described as CAM-related contained tandem duplications. In *T. leiboldiana*, there were 13 orthogroups with a tandem duplication, which is only 2 % of all DE orthogroups. Of these, five belong to multicopy families that are expanded in *T. fasciculata* (10 %), and three that are expanded in *T. leiboldiana* (6 %). Also in this species, no CAM-related orthogroups had tandem duplications. From these results, we do not think that tandem duplications are particularly common in DE orthogroups.

6. The authors mention GDPDL-7 and GLR, whether there is significant dn/ds value in CAM pathway genes in Figure 6.

The comment of the reviewer is not entirely clear - was this a question regarding the significant dN/dS value for GDPDL-7 and GL? Figure 6 provides an overview of the CAM pathway and underlying known genes, indicating which evolutionary changes have been detected in these genes throughout all our analyses. Since our dN/dS analysis did identify adaptive sequence evolution in GLR 2.8, the dN/dS symbol is present near this gene in Fig 6 (see also Fig 4c). We couldn't find a clear connection between GDPDL-7 and CAM photosynthesis in the literature, therefore the gene is not displayed in figure 6.

7. The reviewer requests three way dn/ds value comparison in CAM pathway genes among A. comosus,Kalanchoe, T. fasciculata, and T. leiboldiana for each CAM related gene orthologs. Please check the supp. table in Kalanchoe manuscript.

The philosophy of our study was to take advantage of the relatively short divergence times between *T. fasciculata* and *T. leiboldiana* to avoid detecting an inflated number of genomic changes needed to evolve distinct photosynthetic metabolisms. When working with species that are very distantly diverged, such as eudicot *Kalanchoe* would be from monocot *Tillandsia* (i.e. a divergence in the order of 150 MY), a large number of genomic changes will inevitably be detected, and at least some of them will likely have occurred due to drift (see Heyduk et al. 2019). This makes it more difficult to disentangle which changes are linked to CAM evolution and which are not, compared to analyses on species with shorter divergence times such as the *Tillandsia* species of the study (4.89 MY divergence time (Yardeni et al. 2023; Barfuss et al. 2016; Mendoza et al. 2017)). We also did not include *A. comosus* in our original dN/dS analyses for the same reason. The branch leading to pineapple would be disproportionately longer than those between the two *Tillandsia* species, leading to a study of amino acid changes at an evolutionary timeframe far beyond that which the rest of the study focuses on.

8. Regarding Figure 3b, it is unclear whether A. comosus has been reanalyzed with EDTA, and this needs clarification. The A. comosus genome from CoGe or Phytozome repeat might be based of RepeatMasker analysis.

The EDTA analysis was only run on the *Tillandsia* genomes. For Figure 3b, soft-masked fasta-files were used as input to obtain the sliding-window TE content. The *Tillandsia* genomes were soft-masked using Repeatmasker and the TE library obtained from EDTA. We obtained a soft-masked version of A. comosus assembly F153 from NCBI. We have clarified this in our methods section. Fig. 3B highlights the among-species differences in the landscape of within-chromosomal variation of TE content. Regarding this result, our approach is sufficient to support our conclusions.

9. The reviewer requests more detailed information in Figure 6 regarding the pathway of Crassulacean Acid Metabolism (CAM), particularly focusing on genes detected as differentially expressed, with gene family expansion compare to Kalanchoe (outgroup) and A. comosus, signs of adaptive sequence evolution, or elevated TE insertion counts.

Please see our response to comment 7 for an explanation why we are hesitant to perform analyses between such distantly related taxa as Kalanchoe (eudicot) and *Tillandsia* (monocots), even more so with regard to differentially expressed genes.

10. Figure S7, CPM is not a recommendable choice to show the expression dynamic in CAM and other circadian genes. And this 6 point time course can get a benefit from DESEQ2 with Likelihood Ratio Test instead of MaSigPro.

We thank the reviewer for the comment. We have re-plotted figure S7 and the expression curves in Figure 5c using TPM (transcripts per million) instead, taking into account exonic length per gene. For an explanation as to why we believe maSigPro provides a better approach to study our data than pairwise DE analyses in DESEQ2, see the response to comment 3 of Reviewer 1.

11. The reviewer asked the temporal expression pattern from plot or heatmap (zscore normalize by gene) regarding CAM related genes in figure 6.

We have compiled a heatmap for all CAM-related genes described in figure 6a and b. While the heatmap is compact and rich in information, it mainly illustrates the general shifts in expression between the two species, whereas the circadian oscillation and finer differences between species and timepoints can be better appreciated in the expression curves shown in Figure 5c and in the supplementary file accompanying SI Note 11, which discusses the expression curves in detail. We have incorporated the heatmap in the supplementary (Figure S6) but also kept figure 5c and SI Note 11 with the supplementary file.

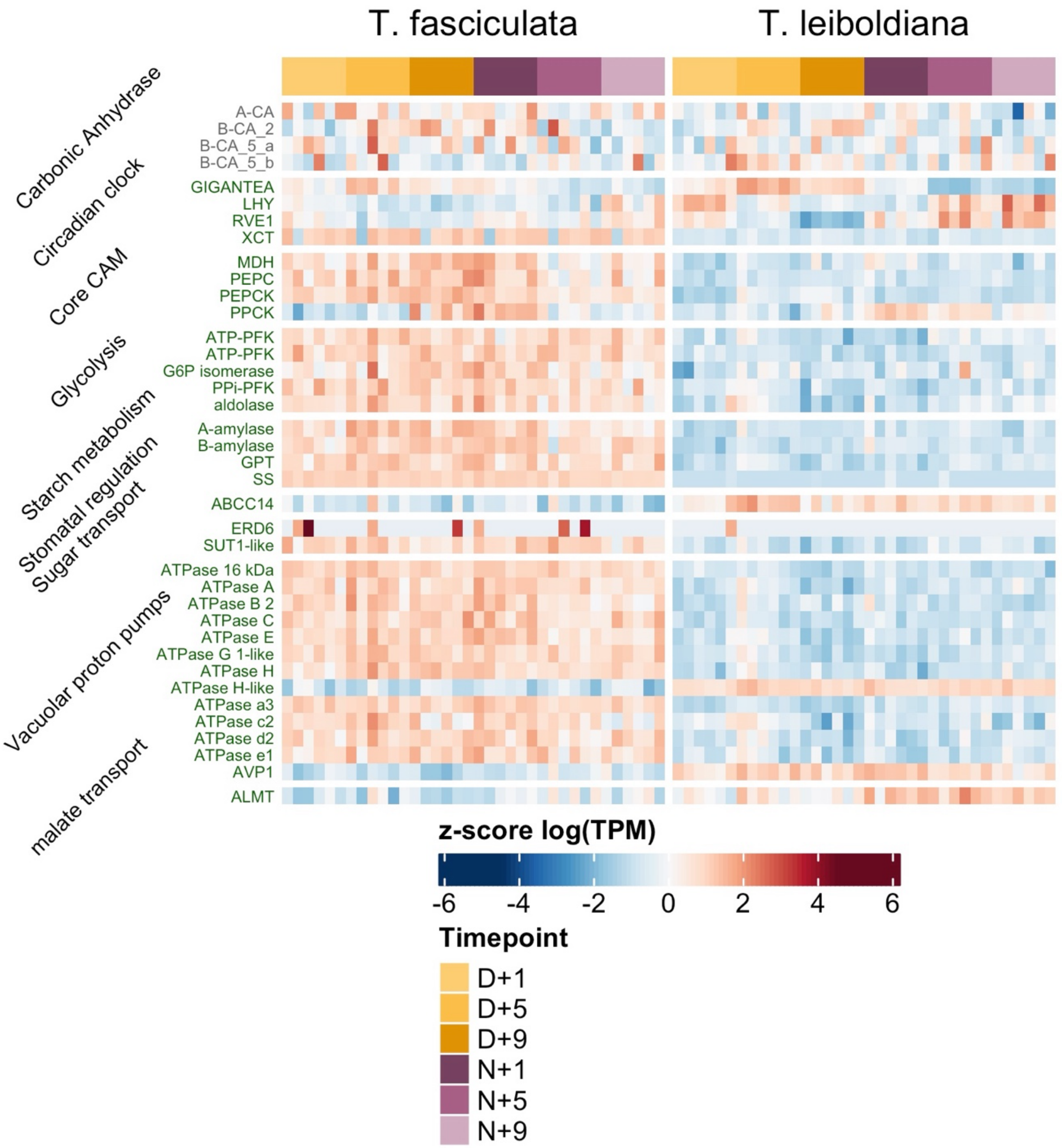

12. Concised verion of 'Note 14: Limitations of this study and future directions' might need to be added to main text discussion part.

Thank you, we have added a few sentences in the discussion.

13. The reviewer asks for clarification in Table 1 about TE insertion and suggests including information on TE families, such as helitrons, especially in the context of their role in expression control in Brassica. In addition, the insertion with 3kbp upstream might be more interesting.

Thank you for these suggestions. We have rerun the same analyses including the 3 kb upstream region of each gene, and on subsets of TE insertions classified as belonging to four different classes: DNA transposons, helitrons, LTR-Copia and LTR-Gypsy. The findings remain largely similar, but we found interesting dynamics for different TE classes. We have amended the Methods and Results section and Table 1 to include these more in-depth analyses.

14. Finally, reviewer request in-depth discussion related to CAM pathway genes.

We provide an extended discussion in SI Note 11, with a more summarized version in the Results section.

Some minors are below.

For Figure 2, the reviewer suggests modifying the shape of the PCA plot to better distinguish between T. leiboldiana and T. fasciculata.

We have modified the shapes of the datapoints in the PCA to distinguish better between species.

The reviewer requests a higher resolution for Figure S13. Cannot read estimated genome size.

Ammended.

The reviewer also reminds the authors to italicize all scientific names in the manuscript.

Ammended.

The reviewer notes that the file 'Tfas_Tlei_rearrangements.pdf' mentioned in the manuscript is missing from the provided GitHub repository.

The PDF should be available in the subsection '4. Synteny' (https://github.com/cgrootcrego/Tillandsia_Genomes/blob/main/4.%20Synteny/Tfas_Tlei_rearrangements.pdf). Do not hesitate to get in touch if the file is still not visible.

References

Barfuss, Michael H. J., Walter Till, Elton M. C. Leme, Juan P. Pinzón, José M. Manzanares, Heidemarie Halbritter, Rosabelle Samuel, and Gregory K. Brown. 2016. "Taxonomic revision of bromeliaceae subfam. Tillandsioideae based on a multi-locus DNA sequence phylogeny and morphology." *Phytotaxa* 279 (1): 1–97.

Buffalo, Vince. 2021. "Quantifying the Relationship between Genetic Diversity and Population Size Suggests Natural Selection Cannot Explain Lewontin's Paradox." *ELife* 10 (August). https://doi.org/10.7554/eLife.67509.

Chen, Jing, Hui Zeng, and Xiumei Zhang. 2021. "Integrative Transcriptomic and Metabolomic Analysis of D-Leaf of Seven Pineapple Varieties Differing in N-P-K% Contents." *BMC Plant Biology* 21 (1): 550.

Chen, Li-Yu, Yinghui Xin, Ching Man Wai, Juan Liu, and Ray Ming. 2020. "The Role of Cis-Elements in the Evolution of Crassulacean Acid Metabolism Photosynthesis." *Horticulture Research* 7 (January): 5.

De La Harpe, Marylaure, Margot Paris, Jaqueline Hess, Michael Harald Johannes Barfuss, Martha Liliana Serrano-Serrano, Arindam Ghatak, Palak Chaturvedi, et al. 2020. "Genomic Footprints of Repeated Evolution of CAM Photosynthesis in a Neotropical Species Radiation." *Plant, Cell & Environment* 43 (12): 2987–3001.

Heyduk, Karolina, Jose J. Moreno-Villena, Ian S. Gilman, Pascal-Antoine Christin, and Erika J. Edwards. 2019. "The genetics of convergent evolution: insights from plant photosynthesis." *Nature Reviews. Genetics* 20 (8): 485–93.

Mendoza, Carolina Granados, Xochitl Granados-Aguilar, Sabina Donadío, Gerardo A. Salazar, María Flores-Cruz, Eric Hágsater, Julian R. Starr, Guillermo Ibarra-Manríquez, Itzi Fragoso-Martínez, and Susana Magallón. 2017. "Geographic structure in two highly diverse lineages of Tillandsia (Bromeliaceae)." *Botany* 95 (7): 641–51.

Ming, Ray, Robert VanBuren, Ching Man Wai, Haibao Tang, Michael C. Schatz, John E. Bowers, Eric Lyons, et al. 2015. "The pineapple genome and the evolution of CAM photosynthesis." *Nature Genetics* 47 (12): 1435–42.

Plomion, Christophe, Jean-Marc Aury, Joëlle Amselem, Thibault Leroy, Florent Murat, Sébastien Duplessis, Sébastien Faye, et al. 2018. "Oak Genome Reveals Facets of Long Lifespan." *Nature Plants* 4 (7): 440–52.

Wai, Ching M., Robert VanBuren, Jisen Zhang, Lixian Huang, Wenjing Miao, Patrick P. Edger, Won C. Yim, et al. 2017. "Temporal and Spatial Transcriptomic and MicroRNA Dynamics of CAM Photosynthesis in Pineapple." *The Plant Journal: For Cell and Molecular Biology* 92 (1): 19–30.

Wai, Ching Man, and Robert VanBuren. 2018. "Circadian Regulation of Pineapple CAM Photosynthesis." In *Genetics and Genomics of Pineapple*, edited by Ray Ming, 247–58. Cham: Springer International Publishing.

Yardeni, Gil, Michael H. J. Barfuss, Walter Till, Matthew R. Thornton, Clara Groot Crego, Christian Lexer, Thibault Leroy, and Ovidiu Paun. 2023. "The Explosive Radiation of the Neotropical Tillandsia Subgenus Tillandsia (Bromeliaceae) Has Been Facilitated by Pervasive Hybridization." *BioRxiv*. https://doi.org/10.1101/2023.11.16.567341.

Yardeni, Gil, Juan Viruel, Margot Paris, Jaqueline Hess, Clara Groot Crego, Marylaure de La Harpe, Norma Rivera, et al. 2021. "Taxon-Specific or Universal? Using Target Capture to Study the Evolutionary History of Rapid Radiations." *Molecular Ecology Resources*, October. https://doi.org/10.1111/1755-0998.13523.

1st Revision - Editorial Decision07-Mar-2024

Dear Dr. Clara Groot Crego:

We have received reviews of your manuscript entitled "CAM evolution is associated with gene family expansion in an explosive bromeliad radiation." On the basis of the advice received, the board of reviewing editors would like to accept your manuscript for publication in The Plant Cell. This acceptance is contingent on revision based on the comments of our reviewers. In particular, please consider the comment by reviewer concerning characterization of transcription factor gene families in your analyses.

Please highlight all changes and include a detailed annotation of changes of the text, with line numbers, and noting your responses to the comments.

To submit your revised manuscript, click:

Link Not Available

If you have any questions about the revision submission procedures, please contact the Editorial Office Staff (tpc-submissions@aspb.org). If you cannot return the revised manuscript within 30 days, please let us know. Otherwise, we will assume that you have elected not to revise the manuscript and withdraw it.

Thank you very much for the privilege of reviewing this work. I look forward to receiving the next version.

On behalf of the editorial board,

Thomas Juenger, Board of Reviewing Editors
Ralph Bock, Senior Editor
Blake Meyers, Editor-in-Chief

The Plant Cell

Reviewer #1 (Comments for the Author):

The authors have addressed my concerns in their detailed revision, and I look forward to seeing this work published.

Reviewer #3 (Comments for the Author):

Overall, the responses are deemed sufficient.
Belows are some addtionational comments.

4. To identify transcription factor, author need to use specific transcription factor domain information such as pfam not Gene Ontology. The method to get Gene Ontology ID is unclear.

7. By including more distantly related species from different evolutionary lineages, such as the comparison between eudicots and monocots, researchers can gain insights into the recurrence and variability of genetic modifications that lead to convergent evolution of CAM. This broader approach could identify shared and unique genetic changes across diverse plant families, providing a more comprehensive understanding of the evolutionary landscape of CAM photosynthesis. Furthermore, while the focus on closely related species reduces the complexity of distinguishing CAM-related changes from those due to drift, it might also overlook the evolutionary experimentation and diversity of CAM-related adaptations that have occurred over longer evolutionary timescales. The evolutionary innovations that confer CAM photosynthesis might involve a combination of ancient shared changes and more recent lineage-specific adaptations, which can only be fully appreciated by examining a broader phylogenetic context.

8. Soft masked A. comosus assembly F153 from NCBI used Windowmasker, which will not consider LTR and plant specific transposon elements.
Aleksandr Morgulis, E. Michael Gertz, Alejandro A. Schäffer, Richa Agarwala, WindowMasker: window-based masker for sequenced genomes, Bioinformatics, Volume 22, Issue 2, January 2006, Pages 134-141, https://doi.org/10.1093/bioinformatics/bti774

Please also note the following:

-The Plant Cell now requires authors to complete and submit an author revisions checklist upon submission of a revised manuscript. The aim of the checklist is to aid authors in preparing a high-quality manuscript, facilitate the review and assessment of revised manuscripts, and help to ensure that journal standards are maintained across the board. If your manuscript is accepted, the completed checklist will be published as supplemental material attached to the article online. Please download a copy of the checklist (pdf fillable form) at this link, for submission with your revised manuscript: https://tpc.msubmit.net/html/Author_Revisions_Checklist.pdf.

-Supplemental materials should be restricted to large datasets and tables, presentation of replicates, and validation of reagents, methods, or genotypes. Any data that are used to support the major claims must be in the main manuscript. Supplemental figure legends must indicate what figure in the main manuscript is supported by the supplemental data presented. Please justify how each of the supplemental figures meet the criteria.

-Sampling methods and the nature of "biological replicates" should be described precisely (i.e. different plants, parts of plants, pooled tissue, independent pools of tissue, sampled at different times, etc.), along with a clear description of and rationale for any statistical analyses conducted. The reader should know exactly what was sampled; what forms the basis of the calculation of any means and statistical parameters reported. This is also necessary to ensure that proper statistical analysis was conducted.

Reviewer #1 (Comments for the Author):

The authors have addressed my concerns in their detailed revision, and I look forward to seeing this work published.

We thank the reviewer for their encouraging comments and careful reading of the revised manuscript.

Reviewer #3 (Comments for the Author):

Overall, the responses are deemed sufficient.
Belows are some addtionational comments.

4. To identify transcription factor, author need to use specific transcription factor domain information such as pfam not Gene Ontology. The method to get Gene Ontology ID is unclear.

We have updated our identification pipeline based on this comment. We compiled lists of transcription factor families in two ways. First, we used the list of identified transcription factor genes in *A. comosus* available on The Plant Transcription Factor Database (PlantTFD: https://planttfdb.gao-lab.org/) to find their homologs in *T. fasciculata* and *T. leiboldiana*. This resulted in 1,057 orthogroups. Because this approach requires existing homology with a pineapple gene present in the database, one could consider it a more conservative method, which may miss newly evolved transcription factors in *Tillandsia*.
In a second approach, we used known InterPro and PFAM domains, which were annotated during functional annotation in Blast2Go, to identify transcription factors of major families. This approach does not require orthology with *A. comosus*, and resulted in 1,168 orthogroups, of which 866 overlapped with the first approach. Because the two separate lists have a large overlap and gave similar results, we merged them into one comprehensive list of 1,359 putative transcription factor families. The results for the merged and the two separate lists are reported in supplementary tables, and overall indicate that TF families have low rates of gene family expansion compared to other gene families. Using the merged list of identified TFs, we searched for TFs in the set if DE genes and studied whether gene family expansion was detectable in this group. We detected 37 TF orthogroups that were DE, of which a few have experienced gene family expansion. While the proportion of multi-copy orthogroups among DE TFs is elevated compared to non-DE gene families, this is not pronounced enough to result in a significant enrichment.
Note that the original results presented as response to this comment were not incorporated into the previous version of the manuscript due to the preliminarily nature of the approach. However, we have confidence in the updated findings and have added them to the main manuscript in its current version.

7. By including more distantly related species from different evolutionary lineages, such as the comparison between eudicots and monocots, researchers can gain insights into the recurrence and variability of genetic modifications that lead to convergent evolution of CAM. This broader approach could identify shared and unique genetic changes across diverse plant families, providing a more comprehensive understanding of the evolutionary landscape of CAM photosynthesis.

Furthermore, while the focus on closely related species reduces the complexity of distinguishing CAM-related changes from those due to drift, it might also overlook the evolutionary experimentation and diversity of CAM-related adaptations that have occurred over longer evolutionary timescales. The evolutionary innovations that confer CAM photosynthesis might involve a combination of ancient shared changes and more recent lineage-specific adaptations, which can only be fully appreciated by examining a broader phylogenetic context.

While we agree with the reviewer on this comment, the broader phylogenetic approach is beyond the scope of this study and has been done before (Brilhaus et al. 2016; Yang et al. 2017). Our study was designed to make use of a different approach which could be considered complementary in understanding CAM evolution as a whole.

8. Soft masked A. comosus assembly F153 from NCBI used Windowmasker, which will not consider LTR and plant specific transposon elements.
Aleksandr Morgulis, E. Michael Gertz, Alejandro A. Schäffer, Richa Agarwala, WindowMasker: window-based masker for sequenced genomes, Bioinformatics, Volume 22, Issue 2, January 2006, Pages 134-141, https://doi.org/10.1093/bioinformatics/bti774

We thank the reviewer for this comment. However, we were unable to find alternative annotations of repetitive content in *A. comosus* that are publicly available. While *A. comosus* does show lower TE content than the two *Tillandsia* species, the TE, GC and gene content across the chromosome is comparable. Nonetheless, we have clarified in the figure caption to not take the absolute difference TE content values literally given distinct masking approaches between genomes.

References

Brilhaus, Dominik, Andrea Bräutigam, Tabea Mettler-Altmann, Klaus Winter, and Andreas P. M. Weber. 2016. "Reversible Burst of Transcriptional Changes during Induction of Crassulacean Acid Metabolism in Talinum Triangulare." *Plant Physiology* 170 (1): 102–22.

Yang, Xiaohan, Rongbin Hu, Hengfu Yin, Jerry Jenkins, Shengqiang Shu, Haibao Tang, Degao Liu, et al. 2017. "The Kalanchoë Genome Provides Insights into Convergent Evolution and Building Blocks of Crassulacean Acid Metabolism." *Nature Communications* 8 (1): 1899.

Dear Dr. Clara Groot Crego:

We are pleased to inform you that your paper entitled "CAM evolution is associated with gene family expansion in an explosive bromeliad radiation" has been accepted for publication in The Plant Cell, pending a final editorial review by a science editor. At this stage, your manuscript will be evaluated by a science editor with respect to its presentation of scientific content, compliance with journal policies, and presentation for a broad readership. The Plant Cell has contracted with Plant Editors (planteditors.com) to provide this service to our authors, and you will soon receive additional information on this process.

Please note that each author needs to link their ORCID identifier to their account in the system before your manuscript can be published. If any authors do not have an ORCID linked to their account, they will receive a message with a link to complete this task. Please ensure that ALL of your coauthors have completed this task as soon as possible.

ASPB offers an OPEN option that allows authors to have their online articles available for free to all users immediately upon publication. For more information about the ASPB OPEN option, refer to the Final Submission Checklist Form.

The Plant Cell and The Arabidopsis Information Resource (TAIR) are collaborating to collect functional annotation data about Arabidopsis genes from authors. This includes information about the gene's molecular function (e.g., kinase activity, ATP synthetase activity), the biological process/es it is involved in (e.g., endosperm development, threonine biosynthesis), its subcellular location (e.g., nucleus, ER), anatomical or developmental expression pattern (e.g., leaf, ovule, flower stage 10, seedling stage), or its partner in a protein-protein interaction (e.g., AT1G01010 interacts with AT1G01020).

If your paper contains results falling into one or more of these categories for Arabidopsis genes, we request that you now submit these data for inclusion in TAIR by filling in the form provided at the following URL: https://www.arabidopsis.org/doc/submit/functional_annotation/123. If you need further clarification on what types of data can be submitted please contact curator@arabidopsis.org.

Finally, we encourage your submission of artwork for the journal cover. Monthly issues will have an online cover image and selected covers will be used for posters, other promotional items, and "wallpaper" for mobile devices. For more information, refer to Cover Submission in the Instructions for Authors https://tpc.msubmit.net/cgi-bin/main.plex?form_type=display_auth_instructions.

We look forward to seeing your paper published.

Sincerely,

The Plant Cell Board of Editors

=====
IMPORTANT REMINDER: PEER REVIEW REPORTS
=====

If you opted to publish a peer review report along with your article during the original submission process, it will be prepared by the editorial staff and publicly posted with your manuscript, inside the zip file that contains any other supplemental material. As a reminder, the peer review report is a public record of all comments from editors and reviewers, as well as your prior responses, as you received them in the decision letters for each draft of your manuscript. If you agreed to publish this report and have changed your mind, or are not sure if you selected this option, please contact the editorial office as soon as possible before signing the license agreement from our publisher.

=====

----FOR ASPB OFFICE USE ONLY (DO NOT EDIT)----
MSID: 36976
Corresponding Author: Dr. Groot Crego
